# Preservation of Underground Microbial Diversity in Ancient Subsurface Deposits (>6 Ma) of the Rio Tinto Basement

**DOI:** 10.3390/microorganisms9081592

**Published:** 2021-07-27

**Authors:** David C. Fernández-Remolar, David Gómez-Ortiz, Per Malmberg, Ting Huang, Yan Shen, Angélica Anglés, Ricardo Amils

**Affiliations:** 1 State Key Laboratory of Lunar and Planetary Sciences, Macau University of Science and Technology, Macau 999078, China; thuang@must.edu.mo (T.H.); yanshen@must.edu.mo (Y.S.); 2 CNSA Macau Center for Space Exploration and Science, Macau 999078, China; aangles@connect.hku.hk; 3 ESCET-Área de Geología, Universidad Rey Juan Carlos, 28933 Móstoles, Spain; david.gomez@urjc.es; 4 Chemistry and Chemical Engineering, Chalmers University of Technology, Kemivägen 10, SE-412 96 Gothenburg, Sweden; malmper@chalmers.se; 5 Centro de Biología Molecular Severo Ochoa (CSIC-UAM), Universidad Autónoma de Madrid, 28049 Madrid, Spain; ramils@cbm.csic.es; 6 Centro de Astrobiología (CSIC-INTA), 28850 Torrejón de Ardoz, Spain

**Keywords:** biomolecules, hyperacidic environments, Iberian pyrite belt, Rio Tinto, underground preservation

## Abstract

The drilling of the Rio Tinto basement has provided evidence of an underground microbial community primarily sustained by the Fe and S metabolism through the biooxidation of pyrite orebodies. Although the gossan is the microbial activity product, which dates back to the Oligocene (25 Ma), no molecular evidence of such activity in the past has been reported yet. A Time of Flight Secondary Ion Mass Spectrometry (ToF-SIMS) molecular analysis of a subsurface sample in the Peña de Hierro basement has provided novel data of the ancient underground microbial community. It shows that the microbial remains are preserved in a mineral matrix composed of laminated Fe-oxysulfates and K- and Na-bearing sulfates alternating with secondary silica. In such a mineral substrate, the biomolecule traces are found in five different microstructure associations, (1) <15 micron-sized nodular microstructures composed of PO_n(2≤n≤4)_^−^, (2) <30 micron-size micronodules containing fatty acids, acylglycerides, and alkanol chains, (3) <20 micro-sized nodules containing NO_n_^−^_(2≤n≤3)_ ions, (4) 40-micron size nodules with NH_4_^+^ and traces of peptides, and (5) >200-micron thick layer with N-bearing adducts, and sphingolipid and/or peptide traces. It suggests the mineralization of at least five microbial preserved entities with different metabolic capabilities, including: (1) *Acidiphilium*/*Tessaracoccus*-like phosphate mineralizers, (2) microbial patches preserving phosphate-free acylglycerides bacteria, (3) nitrogen oxidizing bacteria (e.g., *Acidovorax* sp.), (4) traces of heterotrophic ammonifying bacteria, and (5) sphingolipid bearing bacteria (e.g., Sphingomonadales, and δ-Proteobacteria) and/or mineralized biofilms. The primary biooxidation process acted as a preservation mechanism to release the inorganic ions that ultimately mineralized the microbial structures.

## 1. Introduction

The emergence and evolution of the acidic basin of Rio Tinto have been closely tied to the underground chemolithotrophic communities sustained by the metabolic alteration of sulfide orebodies formed around 350 Ma ago [1]. Such a long-term and large-scale oxidation process have produced two significant impacts in a large area of Southern Spain. The subsurface microbial activity operating since the Oligocene and along the Quaternary [2,3] has induced the formation of several ferruginous deposits in the form of different gossan horizons and terraces [4] which also outcrop other various fluvial basins like the Odiel and Guadalquivir rivers. Furthermore, the release of underground acidic solutions into the surface streams through a diverse and extensive system of low-pH springs has promoted the homeostatic control of the habitat hydrochemistry through ferric ion hydrolysis [5]. As the oldest ferruginous terraces of the Rio Tinto and Odiel river date back to around 2.1 Ma [4], it can be inferred that the acidic communities inhabiting the acidic streams had the same time to occupy, adapt and diversify in the hyperacidic habitat.

The drilling of the basement rocks accomplished by the Mars Analog and Research Technology Experiment (MARTE) and Iberian Pyrite Belt Subsurface Life (IPBSL) projects [6,7,8,9,10,11,12] has provided novel information on the living forms that are involved in the metabolic degradation of the sulfide orebodies. It shows that the Rio Tinto basement is inhabited by a very diverse bacterial community accompanied by Archaea [7], which sustain an intricate cycling network of C, H, N, S and Fe. Inside such a complex frame, the abundance of *Acidovorax* plays an essential role in nitrogen cycling, becoming one of the main agents for the subsurface biooxidation of the orebody [6,7].

Such microbial diversity should have left a molecular imprint in the ancient subsurface deposits precipitated from the sulfide weathering. The record of the ancient microbial subsurface activity has been indirectly recorded in different minerals and microbial structures. They indirectly register ancient metabolic processes that could come from recent times, as the microstructures contain carbon-bearing microbial filaments occurring below the water table [12,13]. The detection of molecular biosignatures has been reported very recently in two different publications that deal with the preservation of biological structures in the Upper Gossan [14], and with the record of microbial structures that have been formed through the metabolic activity of ancient subsurface microbial communities [15]. The Upper Gossan biological structures show a considerable input of eukaryotic life remains. Indeed, the molecular association suggests the preservation of fungal, algal, and plant structures in surface or shallow subsurface environments [14]. On the other hand, the molecular analysis of samples recovered from a 40-meter depth of the stockwork orebody [15] has reported the record of different major organic groups from bacterial origin preserved through the mineralization by abiotic processes and metabolic activity.

In this paper, we carried out an exhaustive study of the molecular composition recorded in the ferruginous deposits occurring in the Rio Tinto subsurface. Such a piece of information has been used to identify the main microbial groups preserved in underground materials of the Cenozoic age. The molecular composition was also referred to similar data reported from the Upper Gossan [14]. Comparing both areas, in turn, will show how changes in the community composition are recorded from shallow to deeper areas under acidic and oxidizing conditions. Furthermore, we completed the metabolic characterization for the microstructures of biological origin that has been reported recently in the same ferruginous materials [15]. It includes different mineral deposits inside the microstructures that have been already identified as the result of nitrogen oxidizers.

## 2. Geological Settings

**Figure 1:**
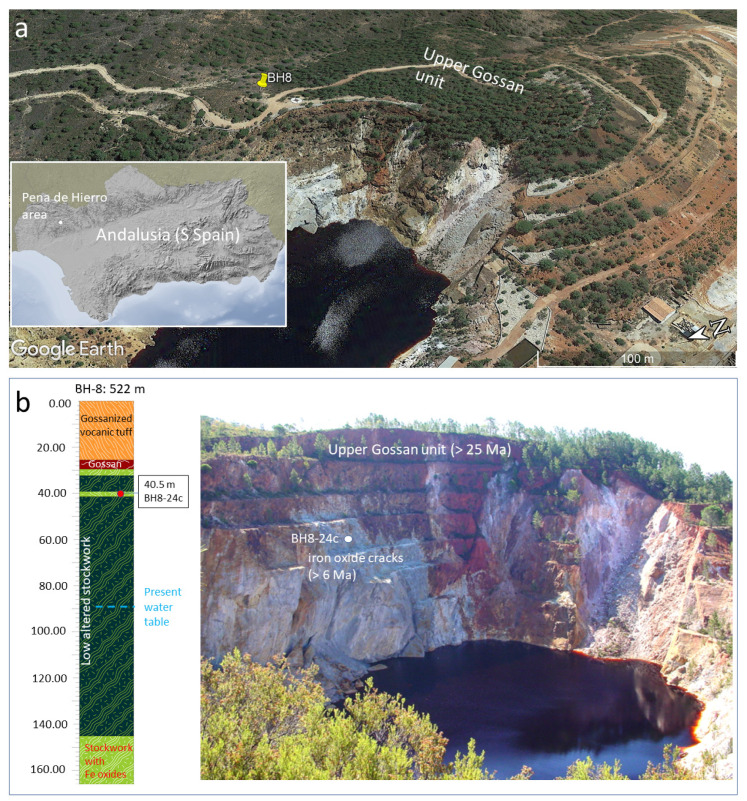
Composition of pictures and figures showing the geological features of the BH8 drilling site of Peña de Hierro. (**a**) Google Earth aerial image displaying the BH8 drilling location below the Upper Gossan (25 Ma) materials topping Peña de Hierro. (**b**) Geological section of the drilled geological units (gossanized volcanic tuff, gossan, stockwork with Fe oxides, and low altered stockwork) where sample BH8-24c was collected. The units can be partially recognized (**b**) at the material outcrops that are exposed in the pit lake of the abandoned mine, where different faults can be recognized affecting the gossan and the Carboniferous basement. The geological section and outcrop picture have been produced and owned by David Fernandez-Remolar.

The samples analyzed come from the Cenozoic gossan deposits that are topping the volcano-sedimentary complex in the Peña de Hierro location (Figure 1a). They were collected through the drilling operations performed under the MARTE (Mars Analog and Technology Experiment) project that collected more than 250 m of cores in three different targets [13]. The basement composition and geology have been thoroughly described in different papers [1,13,16] and will not be detailed here. In short, it consists of a Visean volcano-sedimentary complex that has been exposed to hydrothermal activity, which is followed by alternating greywackes and dark shales [17]. Hydrothermalism has been recorded in form of two different by-products as massive sulfide orebodies and a network of stockwork veins composed of quartz and pyrite. They result from the formation of sulfide orebodies in two different settings corresponding to the precipitation of sulfides on the ocean bottom, and from the hydrothermal alteration of marine bed formed by the volcanoclastic and extrusive igneous materials of Visean age [17].

Since the Oligocene [2,3], Carboniferous sulfides have been the source of the acidic and ferruginous solutions for the gossan formation and for the weathering products in the Rio Tinto area. The basement drilling has shown that, in terms of rock weathering and ferruginous deposit content, the substrate in the Peña de Hierro location [13] is composed of four different units (Figure 1b) that include from the top to the bottom: (1) an upper gossanized volcanic tuff, (2) a massive to brecciated gossan, (3) a weathered stockwork and, (4) an intact stockwork with very few traces of weathering and secondary ferruginous salts and oxides. As the rock host is non-porous, the distribution of the four weathering units in the subsurface is strongly controlled by faults and large fractures (Figure 1b), which has propagated the weathering following an unequal underground flow [1]. In this context, the gossan is a complex geological entity constituted by various ferruginous subunits [3], whose formation has resulted from the climatic evolution and basement structure. At the Peña de Hierro area, there are three different fault systems that are trending ESE-WNW, NNE-SSW and NNW-SSE. The orientation ESE-WNW corresponding to ductile shear zones developed during the Variscan orogeny (310–270 Ma) [18] that defined major boundaries of the different tectonic units. On the other hand, the orientations NNE-SSW and NNW-SSE correspond to a conjugate system of brittle strike-slip faults associated with a Late-Variscan deformation stage during the collapse of the Variscan orogen that occurred about 270 Ma ago. Some faults that were lately reactivated during the NW-SE compressional event (<15–10 Ma) linked to the Alpine orogeny [19]. It played an essential role in forming the main conduits that were used by the meteoric solutions, which propelled the weathering and biooxidation of the sulfide orebodies in the subsurface of Peña de Hierro [1].

In Peña de Hierro, the two units described above as massive and brecciated gossan, and weathered stockwork are the main members of the gossan complex [13]. They were likely formed in different weathering events associated with a couple of thermal climatic episodes corresponding to the Late Oligocene and Late Miocene [2]. The age ranges for such weathering episodes could average <25 Ma and 6 Ma, respectively. While the older Upper Gossan materials are primarily composed by hematite and goethite, the younger and lower gossan by goethite and subordinate jarosite [3]. However, the distribution of the different ferric compounds in the gossan is very heterogeneous. In this regard, the molecular analysis of the Upper Gossan has shown the occurrence of relicts of different sulfates [14], suggesting that the diagenetic maturation follows a very heterogeneous evolution.

The gossan formation halted several millions of years until a drop of the water table associated with the late Messinian saline crisis that reached the stockwork orebody by >6 Ma [20]. In this weathering episode, the fracture infilling by laminar ferric deposits expanded the gossan materials to the basement deeper areas. Finally, a late alteration episode took place after a local neotectonic uplift and/or climatic changes by >1–2 Ma [3]. This last episode likely drove the formation of the ferruginous terrace system of the Rio Tinto headwaters [4]. The gossan has originated from a complex interaction between direct and indirect chemolithotroph attack of the pyrite (FeS_2_) orebody that ended in the formation of acidic sulfates [21]. The main process is led by few microbes such as *Acidithiobacillus ferrooxidans, Ad. thiooxidans*, and *Leptospirillum ferrooxidans* [22] which oxidize sulfur and iron via the following routes:

FeS_2_ (s) + H_2_O (l) + 3.5·O_2_ (aq) → Fe^2+^ (aq) + 2·SO_4_^2−^ (aq) + 2·H^+^ (aq) (direct biooxidation of sulfur) (1)

Fe^2+^ (aq) + 1/4·O_2_ (aq) + H^+^ (aq) → Fe^3+^ (aq) + 1/2·H_2_O (l) (biooxidation of released Fe^2+^) (2)

where O_2_ is used as electron acceptor for the oxidation of FeS_2_ and free Fe^2+^.

The ferric cation resulting from the microbial oxidation of Fe (II) becomes subsequently in an abiotic oxidizing agent of the pyrite sulfide anion where Fe^2+^ is newly formed as shown in the following reaction:

FeS_2_ (s) + 14·Fe^3+^ (aq) + 8·H_2_O (l) → 15·Fe^2+^ (aq) + 2·SO_4_^2−^ (aq) + 16·H^+^ (aq) (3)

The solutions resulting from the sulfide oxidation are eventually exposed to oversaturation mainly producing acidic sulfates like jarosite (e.g., H_3_O^+^Fe_3_(OH)_6_(SO_4_)_2_) and schwertmannite (Fe_8_O_8_(OH)_6_(SO_4_)) as described in the reaction as shown by Fernández-Remolar et al. [4]:

3·Fe^3+^ (aq) + M^+^ (aq) + 2·SO_4_^2+^ (aq)+ 6·H_2_O (l) → MFe_3_(OH)_6_(SO_4_)_2_ (s) + 6·H^+^ (aq) (4)

where M = Na, K or H_3_O, and

8·Fe^3+^ (aq) + SO_4_^2−^ (aq) + 14·H_2_O (l) → Fe_8_O_8_(OH)_6_(SO_4_) (s) + 22·H^+^ (aq) (5)

Interestingly, new data has shown that *Acidovorax* is also involved in the cycling of different compounds through redox metabolic processes using different nitrogen compounds as electron acceptors [6]. The microbial activity of such bacteria has been likely recorded in form of micron-sized microstructures that correspond to sections of filamentous sheaths enriched in nitrogen [15].

The maturation of the gossan materials comprehends a succession of mineral compounds that start as different ferric oxysulfates and oxyhydroxides (e.g., jarosite, schwertmannite, and nanophase iron oxyhydroxides) and end in more crystalline dehydrated phases like hematite [4]. Therefore, there is a mineralogical gradient from younger to older gossan materials, which would become enriched on more anhydrous and more crystalline phases over time. This is observed in samples collected at different depths of the Peña de Hierro gossan, where hematite occurs at a much higher concentration in the Upper Gossan unit when compared with samples collected at deeper areas of the complex [13]. In this context, it is expected that the sample studied in this work that has been collected at 40 m depth should have a significant concentration of sulfates when compared with materials from the upper part of the gossan.

## 3. Materials and Methods

### 3.1. Sample Collection

The sample was collected at the lower part of the gossan (40 m depth) [13] by drilling the Peña de Hierro basement (Figure 1a) during the field operations of the MARTE project [23]. Procedures to control core contamination during the drilling and the sample collection from the core are shown in detail by Fernández-Remolar et al. [13] and will not be discussed here. Sampling was conducted on the basis of the distribution of the ferruginous deposits in the Peña de Hierro basement, which suggests at least two main episodes of alteration [2]. We assume that the materials collected at 40 m depth were likely formed after an unstable dropping water table.

The sample studied in this work comes from the sample BH8-24c that was obtained at a depth ranging between 39 and 40 m (Figure 1b) [13]. It was collected from silica-rich materials with partially or totally dissolved pyrite crystal. Such crystals were affected by cm-sized cracks that are currently infilled by millimeter size laminas of iron oxides, which would eventually have preserved biological structures on its surface (see Figure 7 in Fernández-Remolar et al. [13]). Indeed, sample BH8-24c consists of a < 1 mm thin lamina that contained ferruginous materials (Figure 1b), which precipitated out from an acidic solution sourced in the stockwork pyrite dissolution.

### 3.2. Sample Preparation

Thin section of sample BH8-24c was prepared for mineral and textural analysis and the molecular surface was analyzed by ToF-SIMS [24]. They were extra-polished by using a 0.3 μm alumina paste to reduce surface imperfections that produce interferences during the analysis. To prevent contamination for such analysis, the sample surface was cleaned by sputtering with a 100 nA 500 3 V oxygen ion beam for 3 s.

### 3.3. ToF-SIMS Analysis

The molecular data was obtained through performing a ToF-SIMS that characterize the distribution of positive and negative ions in a sample. For this reason, it can provide essential information of preserved biomolecules to establish a direct association between the mineral matrix and molecular biosignatures in the sample. The main analytic conditions for the sample analysis have been widely described in other papers [24,25] and will not be detailed here. In a few words, the molecular surface analyses were done using a TOF-SIMS IV (ION-TOF, Münster, Germany) under a pressure of 5 × 10^−9^ mbar. The sample was exposed to a pulsed ion beam of Bi_3_^2+^ at 25 keV, which was operated with a 20 ns pulse width, 0.3 pA pulsed ion current for a dosage lower than 5 × 10^11^ ions·cm^−2^. The released secondary ions were detected with a reflector time-of-flight analyzer, a multichannel plate (MCPs). The subsequent signal was accepted by a time-to-digital converter (TDC), which was conducted under a charge neutralization with a low energy (20 eV) electron flood gun. Secondary ion spectra were obtained from a randomly rasterized surface area of a 500 μm square. Secondary ions were extracted with 2 kV accelerating voltage and post-accelerated to 10 keV kinetic energy just before hitting the detector.

Mass spectral acquisition and image analysis were performed within the ION-TOF Ion Spec and Ion image software (version 6.8). The analysis of the molecular fragmentation pattern was completed through the Chemical online tool [26] and the open source mass spectrometry tool Mmass [27]. Chemspider, METLIN and LIPID MAPS Structure databases [28,29,30] were used as a source of information to identify molecular fragments and compounds.

Based on the observation of microfabric and microtextural features, we selected three different target areas (TA1, TA2, and TA3) for the collection of TOF-SIMS ion-induced secondary electron images. Such a characterization was used to understand the association between texture, mineralization, and the distribution of preserved biological compounds. Once the molecular data was collected in the different TAs, a two-way data analysis was done in TA1 and TA2. It was accomplished through: (1) the identification of the main morphological groups that are characterized by the compound distribution, and (2) by the identification of the main biomolecular categories was performed by the sample spectral analysis in such three different TAs. The data integration between the imaging and spectral analysis was also assisted by performing a principal component analysis (PCA) [31]. In this study, PCA has revealed the association between molecular fragments and morphological groups that have not been observed by direct image analysis. Such an analysis has provided additional information for identifying tentatively compositional variations in the same group of microstructures or their microbial origin. The PCA considered a variable number of masses corresponding with three different groups of ions including the negative ions of major groups (8 peaks for TA1, and 9 peaks for TA2), cations of N-bearing compounds like amino acids (32 peaks for TA1, and 30 peaks for TA2), and fatty acids (19 peaks for TA1, and 25 peaks for TA2). Meanwhile, our ToF-SIMS analysis was also supported by SEM-EDAX data (Scanning Electron Microscope-Energy Dispersive X-Ray Analysis) that can provide essential information of both major elemental composition and microstructure (Supplementary Figure S1). The equipment description, analytic conditions and sample preparation have been discussed widely in other works [12,13] and will not be described here.

## 4. Results

### 4.1. ToF-SIMS Image Analysis

#### 4.1.1. Sample Microstructure

**Figure 2:**
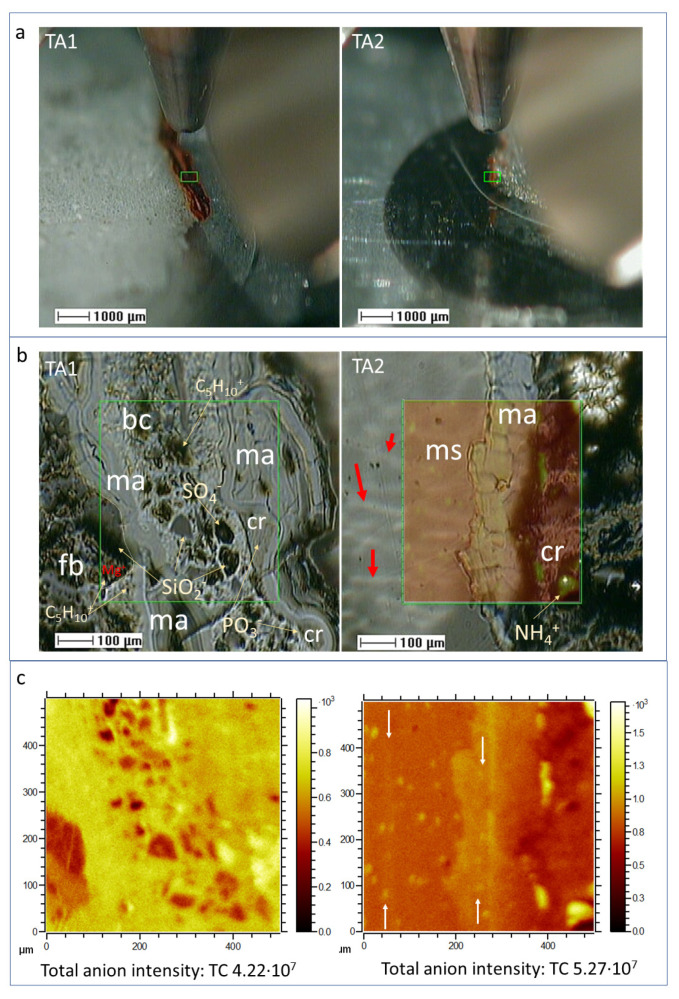
Different images of the target areas 1 and 2 (TA1, and TA2) in the sample BH8-24c showing the fabric and microstructure of the mineral deposits infilling the hydrothermal host rock. (**a**) Images taken by the ToF-SIMS visible camera where it is possible to appreciate the different ferruginous and sulfatic materials filling a crack in the Carboniferous basement. (**b**) Microimage obtained by the ToF-SIMS SEM probe showing the internal fabric and structure of sample BH8-24c in TA1 and TA2. They are composed by four different layered units including fibrous and cryptocrystalline (fb), microlaminated (ma), microbrecciated (mb), and glassy (ms), as well as a crenulated patchy unit (cr). Interestingly the glassy layer (ms) shows internally crossed by some filamentous microstructures (red arrows). (**c**) ToF-SIMS image displaying the total intensity counts that provide additional information of the sample microstructure in form of different sets of micronodular elements, fine laminas, and microstructure alignments (see white arrows).

The ToF-SIMS SEM images show that the laminated ferruginous deposits infilling the ancient hydrothermal materials are complex. SEM images obtained in TA1 and TA2 by the ToF-SIMS reveal at least four different units (Figure 2; Supplementary Figure S1 and Table S1) characterized by the internal microstructure. The most abundant microfabric unit corresponds with sets of <80-micron thick layers internally formed by fine laminas with a thickness of <1 micron. It is followed in extension by a dark cryptocrystalline fabric with fibrous patches (Figure 2). In TA1, it has been found a breccia-like layer with a thickness of a 300-micron cryptocrystalline matrix enclosing diverse clastic elements, which exhibit a subangular morphology with a size of 50 microns. TA2 is greatly composed by a thick glassy layer (>400 microns) including a stack of fine tenuous laminations (1 < micron thickness). The glassy layer has a sharp contact with the laminated part. It is crossed by several > 100 micron-long sinuous alignments with an average thickness < 10 microns showing a parallel orientation. Furthermore, in localized TA1 areas, some crenulated structures are found filling depressions left by the sinuous topography of the laminated structures (Figure 2). The crenulated fabric is composed by the accretion and stacking of fine sheets which show thickness lower than 5 microns.

The observation of the different microstructures is supported by the SEM-EDAX analysis of sample BH8-24c (Supplementary Figure S1a,b), which show layers with distinct structure. The finely laminated microstructure consists of sets of 50 micron-sized layers that result from stacking laminas with an average thickness of 1 micron. The EDAX analysis of the laminations shows the main composition (>10 Atomic %) with Fe, O, and Si with a secondary concentration (<7 % Atomic) from C, N, and S. The SEM images also show a flaky layer containing phyllosilicates that might correspond with the fibrous microstructure. The EDAX analysis of this layer shows a very diverse elemental composition including (>25 Atomic %) Si, O, Al, and Fe, as well as (<2 Atomic %) Mg, Na, and K (Supplementary Figure S1a,b), which agrees with the composition of Fe- and Mg-bearing phyllosilicates found in the host rock [16].

#### 4.1.2. Distribution of Inorganic Ions

**Figure 3:**
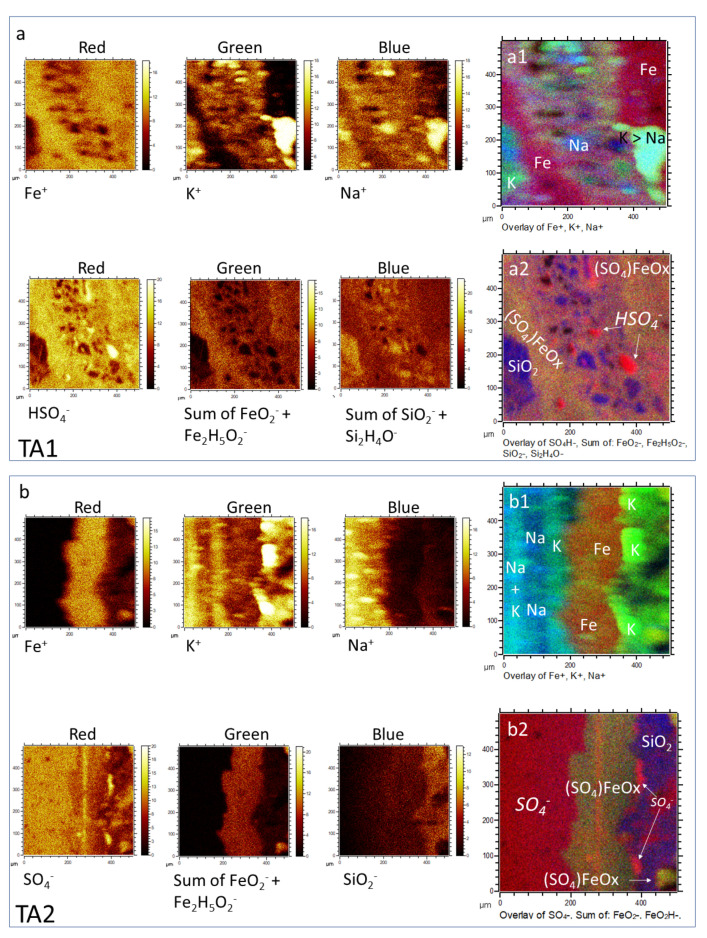
Characterization of the layer mineral composition through the distribution of inorganic cations (Fe^+^, Na^+^, and K^+^) and anions (SO_4_^−^, FeO_2_^−^ + Fe_2_H_5_O_2_^−^, and SiO_2_^−^ + Si_2_H_4_O^−^) through a red, green blue graph (RGB) merging different ion images. Inorganic composition in TA1 (**a**) and TA2 (**b**) showing five distinctive mineral areas like ferruginous and sulfatic-rich ((SO_4_)FeOx), silica-rich (SiO_2_), sulfur enriched (SO_4_^−^), and heterogeneous, which reflects a greater compositional variability of the microbrecciated layer. They follow the occurrence of the different layered units described in Figure 2b; where (1) ferruginous and sulfatic-rich composition meet the microlaminated layer (ma) and the matrix of the microbrecciated layer (mb), (2) the silica-rich area follows the fibrous and cryptocrystalline layer (fb), and (3) and the sulfate area depleted in Fe corresponds with the glassy layer (ms). Furthermore, the compositionally heterogeneous area with SiO_2_ and SO_4_^−^ correspond with different microclasts that are embedded in the microbrecciated layer (mb). The cation distribution provides some additional information regarding the geochemical composition of the different units. It is observed an association between K^+^ and Na^+^ with the silica and sulfate-rich and iron-depleted areas. Indeed, K^+^ is highly associated with the crenulated unit (cr in Figure 2b) that is enriched in phosphate (Figure 4a,b). K^+^ and Na^+^ also enhance the internal microstructure (b2) of the glassy layer (ms) where they occur forming thin laminas and enlarged nodular-like appearance that meets the filamentous structures shown in Figure 2b. The ToF-SIMS images were obtained from a rasterized surface area of a 500 μm square.

The spectral capabilities of the ToF-SIMS have detected different peaks that fit various inorganic ions, which provide essential information on the sample mineral matrix. In TA1 and TA2, high intensity peaks of positive masses (I > 1200 cps) found at 22.99, 23.99, 26.98, 38.96, 39.96, 55.94, 71.93, and 143.86 fit well with the cations Na^+^, Mg^+^, Al^+^, K^+^, Ca^+^, Fe^+^, FeO^+^, and Fe_2_O_2_^+^ (Figure 3; Supplementary Table S1).

Negative masses of more complex ions have been found at 46.00, 59.98, 62.00, 62.97, 63.96, 71.93, 77.97, 78.97, 79.97, 87.93, 95.96, 98.96, 118.95, 120.95, 148.91, and 168.89 corresponding to NO_2_^−^, SiO_2_^−^, NO_3_^−^, PO_2_^−^, SO_2_^−^, FeO^−^, AlH_3_SO^−^, PO_3_^−^, SO_3_^−^, FeO_2_^−^, SO_4_^−^, H_3_S_2_O_2_^−^, NaPO_4_H^−^, Si_2_O_4_H^−^, Fe_2_H_5_O_2_^−^, and FeSO_5_H^−^. The occurrence of these positive and negative ions suggests the presence of at least five groups of inorganic compounds that include nitrates, phosphates, silicates, and iron oxyhydroxides/oxysulfates.

**Figure 4:**
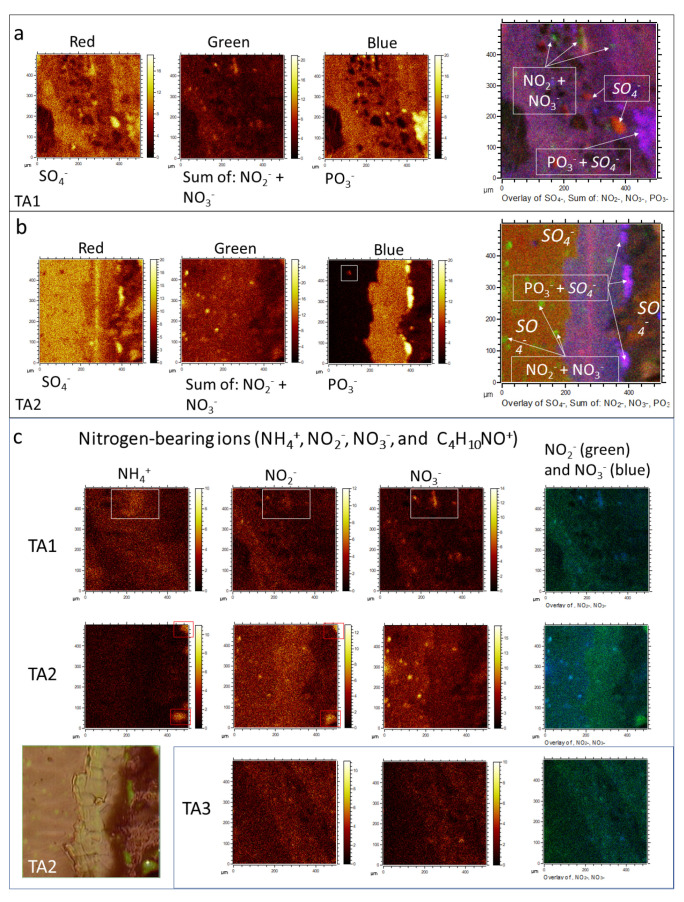
Distribution of main anions like SO_4_^−^, NO_2_^−^ + NO_3_^−^, and PO_3_^−^ with potential biological origin in TA1 (**a**) and TA2 (**b**). The NO^−^_n(2≤n≤3)_ ions occur forming <20 micron-sized nodular structures inside the ferric microlaminated (**a**) and the glassy (**b**) layers. N-bearing anions are also found forming a few larger (<40 microns) nodular structures (**b**) inside the silica-rich unit. Furthermore, PO^−^_n(2≤n≤4)_ appear as patches (**a**) or discontinuous laminas (**b**) limiting two different layer units. Phosphate also occurs forming micronodular structures (<20 microns) inside the microlaminar layer. An isolated phosphate-rich micronodule (**b**) is also found in the glassy layer (white square). (**c**) Shows a composition of ToF-SIMS images displaying the distribution of the different N-bearing ions like NH_4_^+^, NO_2_^−^, and NO_3_^−^ in target areas TA1, TA2 and TA3. The ToF-SIMS images were obtained from a rasterized surface area of a 500 μm square.

The combination of the spectral information with image distribution of the inorganic ions shows a close association with the sample microstructure in the three different TAs (Figure 3 and Figure 4; Supplementary Figure S2 and Table S1). Particularly, the appearance of S- and Fe-bearing ions (FeO^−^, FeO_2_^−^, Fe_2_H_5_O_2_^−^, and FeSO_5_H^−^) fall in the areas with finely laminated microstructures (Figure 3 and Figure 4). Some other microstructures show a maximum concentration in S-bearing anions but depleted in Fe-bearing ions (Figure 3a1,a2,b1,b2). Both cations K^+^ and Na^+^ occur in the PO_3_-bearing patches (Figure 3a,b and Figure 4a,b; Supplementary Figure S2).

In addition, K^+^ also show a higher affinity with the S-bearing anion microstructures, while Na^+^ distribution is more akin to microstructures of Si-bearing anions (Figure 3a,b; Supplementary Figure S2). The NO_n_^−^_(2≤n≤3)_ distribution (Figure 4c) appears as tinny (<10 microns) ovoidal micronodules scattered throughout the analyzed TAs with no clear association with other ions (Figure 4a–c). Instead, they show a dissimilar arrangement with the microstructures defined by the occurrence of the Si-bearing ions like SiO_2_^−^, and Si_2_H_4_O^−^. Although the appearance of NO_2_^−^ and NO_3_^−^ is very similar, it is slightly disparate in intensity and distribution (Figure 4c). Moreover, NO_2_^−^ partly meet NH_4_^+^ in patches or large nodular structures (>50 microns), while NO_3_^−^ is entirely dissimilar with the NH_4_^+^. Therefore, the distribution of NH_4_^+^ and NO_2_^−^ is similar to the occurrence of some N-bearing organic cations (Figure 4c).

#### 4.1.3. Morphological Groups

**Figure 5:**
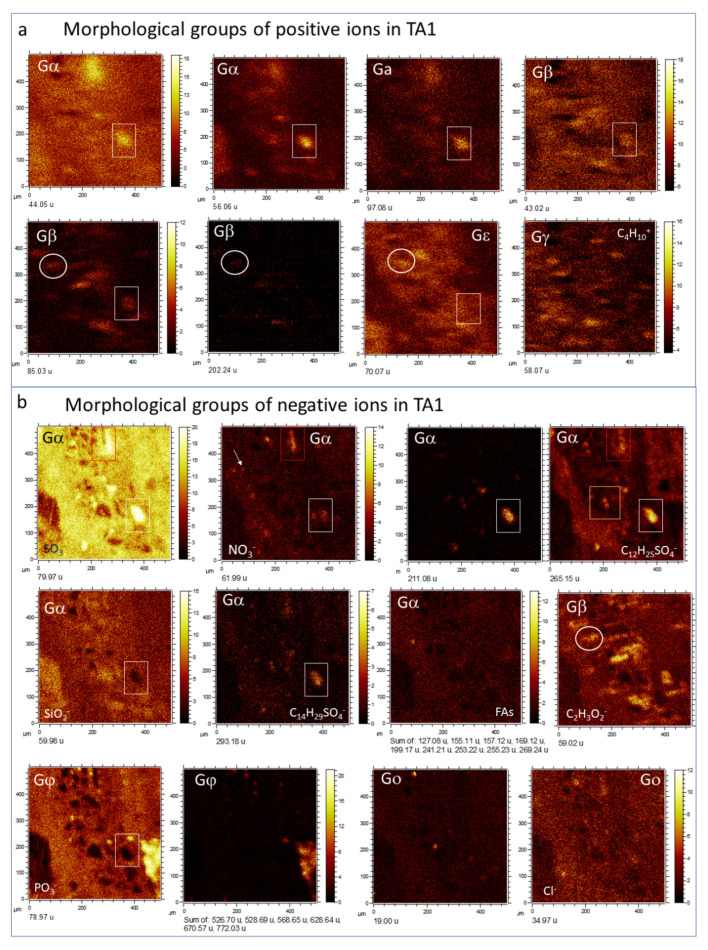
Characterization of the different morphological groups through the ToF-SIMS images in TA1. (**a**) Identification of four morphological groups (Gα, Gβ, Gε, and Gγ) defined by the cation distribution. (**b**) Same as in (**a**) but using the anion distribution which allowed to recognize two additional groups (Gφ, and Gο). The white square points to the location of the SO_4_-rich clasts of the microbrecciated layer as a reference, while the white circular frame traces some straight microstructures that are not been previously identified. The ToF-SIMS images were obtained from a rasterized surface area of a 500 μm square.

**Figure 6:**
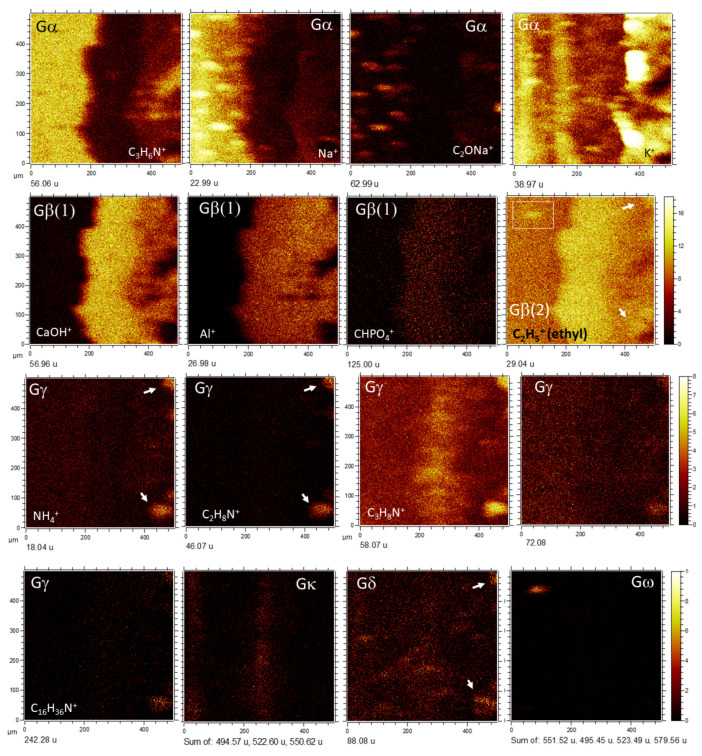
Characterization of the morphological groups by using the cation ToF-SIMS images in TA2. It has led to the identification of six different groups including Gα, Gβ, Gγ, Gκ, Gδ, and Gω. Gα shows two variations that have been named Gα(1) and Gα (2) which are defined by the absence/presence of the nodular structures enriched in NH_4_^+^. The white arrows point to the nodular microstructures associated with NH_4_^+^ and different N-bearing cations characterized as amino acids. K^+^ and Na^+^ classified as Gα show a somewhat different distribution as they follow the occurrence of the filamentous-like microstructures crossing the glassy layer (ms in Figure 2b). Furthermore, K^+^ also reveals the presence of two ~50 micron-sized lamina inside the glassy layer as well. It also has a high intensity in the phosphate-bearing patches. The white square points to an intensity increase in a very localized area that has been assigned to Gω. The ToF-SIMS images were obtained from a rasterized surface area of a 500 μm square.

**Figure 7:**
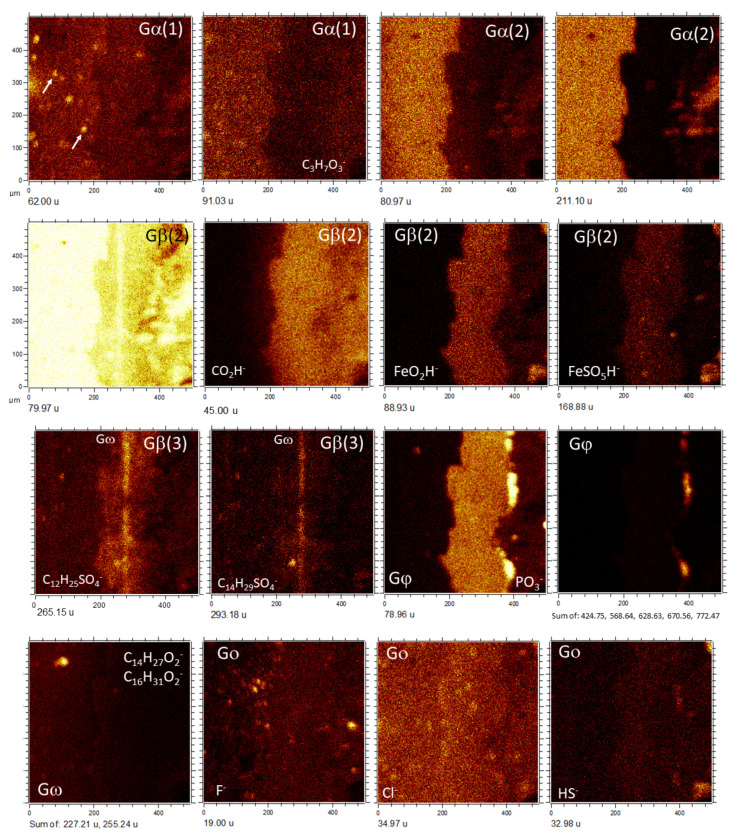
Identification of the morphological groups through the anion ToF-SIMS images in TA2. As for cations, it has been characterized Gα, Gβ, Gγ, and Gφ, plus a new group Gο that includes the distribution of different anions with unrelated patterns. Minor pattern changes inside Gα and Gβ have suggested to separate them into several subgroups as it has also observed for the TA2 cation groups. The HS^−^ distribution classified into Gο follows the phosphatic discontinuous laminas and the NH_4_^+^-bearing nodules, which are associated with the presence of amino acids. The ToF-SIMS images were obtained from a rasterized surface area of a 500 μm square.

By combining molecular and secondary electron ToF-SIMS imaging, it has been possible to identify the following morphological groups based on the molecular distribution of positive and negative ions (Figure 5, Figure 6 and Figure 7; Supplementary Figure S2, Tables S1 and S2) in the three different TAs:

*Group α(Gα).* Defined by the presence of the positive organic ions like 42.04 (C_2_H_4_N^+^), 44.05 (C_2_H_6_N^+^), 54.04 (C_3_H_4_N^+^), 68.03 (C_2_H_2_N_3_^+^), 70.04 (C_2_H_4_N_3_^+^), 71.06 (C_3_H_7_N_2_^+^), 72.05 (C_2_H_6_N_3_^+^), 83.06 (C_4_H_7_N_2_^+^), 107.06 (C_6_H_7_N_2_^+^/C_7_H_7_O^+^), 109.08 (C_6_H_9_N_2_^+^), 113.08 (C_6_H_11_NO^+^), 122.08 (C_6_H_8_N_3_^+^), 123.10 (C_8_H_13_N^+^), 124.10 (C_6_H_10_N_3_^+^/C_7_H_12_N_2_^+^), 126.11 (C_7_H_12_N_2_^+^), and 138.11 (C_7_H_12_N_3_^+^/C_8_H_14_N_2_^+^). In TA1, such a set of positive fragments outlines part of the 20 to 80-micron size clasts occurring inside the matrix of the microbrecciated layer. This group of clasts is oriented along the main structure (Figure 2 and Figure 3a). Some of the micronodules correspond with the subangular elements observed by the secondary electron ToF-SIMS images (Figure 2, Figure 3a, Figure 4a and Figure 5a,b). They are defined by an intensity found in SO^−^_(2≤n≤4)_ ions (*m/z* 62.97/79.97/95.95); as well as in SO^−^_(3≤n≤5)_-bearing negative adducts like C_5_H_9_SO_4_^−^ (167.02), C_12_H_23_SO_4_^−^ (263.13), C_12_H_25_SO_4_^−^ (265.15), C_12_H_26_SO_4_^−^ (266.15), C_12_H_27_SO_4_^−^ (267.15), C_13_H_27_SO_4_^−^ (279.17), C_13_H_28_SO_4_^−^ (280.17), C_14_H_29_SO_4_^−^ (293.18), C_14_H_30_SO_4_^−^ (294.18), C_14_H_31_SO_4_^−^ (295.16), C_14_H_29_SO_5_^−^ (309.17), C_16_H_33_SO_5_^−^ (337.20), C_20_H_33_SO_3_^−^ (353.22) and C_17_H_35_S_2_O_4_^−^ (367.22), while are highly depleted in Fe^+^ (Figure 3a). Some other anions like 211.09, 225.11, 227.13, and 239.11 could correspond with C_14_H_13_NO^−^, C_15_H_15_NO^−^, C_15_H_17_NO^−^, and C_16_H_17_NO^−^. The ions 227.20 and 323.20 are assigned to C_14_H_27_O_2_^−^ and C_22_H_27_O_2_^−^, which have carboxylic groups also following the Gα pattern.

The same set of Gα cations and anions listed above (Supplementary Table S2) shows a high concentration in the TA2 glassy layer and different fibrous areas, where they appear discontinuously and their patches showing 40 micron-size nodular microstructures (Figure 2 and Figure 3b). These nodular structures are crossed by Na^+^ and K^+^-rich linear units that concur with the alignments observed in the SEM ToF-SIMS image of TA2 (Figure 2 and Figure 3b). Various ions (<20-micron) circularly scattered by negative fragments showing a similar distribution like Na^+^ and K^+^ (Figure 6 and Figure 7). These negative fragments include *m/z* 42.00, 49.02, 62.00, 90.01, and 91.03, corresponding to NO_2_^−^, NO_2_H_3_^−^, NO_3_^−^, C_2_H_4_NO_3_^−^, and C_2_H_5_NO_3_^−^, respectively.

The occurrence of two different groups of TA2 with circular microstructures defined by negative fragments following two different patterns described as Gα(1) and Gα(2) (see Figure 7). The Gα(1) pattern corresponds to a maximum concentration of negative ions in the glassy layer (Figure 2) that eventually determine the NO_n_^−^_(2≤n≤3)_-bearing microspheroidal elements. They include a combination of inorganic and organic negative ions. Inoganic ions refer to79.97 (PSNH_3_^−^), 80.97 (CSNNa^−^), 81.96 (CHSNNa^−^), 95.97 (SNO_3_H_3_^−^), 107.99 (C_5_H_2_SN^−^), and 105.98 (C_2_H_4_S_2_N^−^); while organic negative ions contain 26.01 (CN^−^), 41.01 (CHN_2_^−^), 44.02 (CH_2_NO^−^), 50.01 (C_4_H_2_^−^), 51.03 (C_4_H_3_^−^), 58.04 (CH_4_N_3_^−^/C_2_H_4_NO^−^), 59.01 (C_2_H_3_O_2_^−^), 60.01 (C_2_H_4_O_2_^−^/CH_2_NO_2_^−^), 61.01 (CH_3_NO_2_^−^), 62.02 (CH_4_NO_2_^−^), 64.03 (C_5_H_4_^−^), 65.01 (C_3_HN_2_^−^), 65.05 (C_5_H_5_^−^), 66.01 (C_2_N_3_^−^/C_4_H_2_O^−^), 83.03 (C_4_H_5_NO^−^), 83.06 (C_4_H_7_N_2_^−^), 84.02 (C_2_H_2_N_3_O^−^/C_4_H_4_O_2_^−^), 84.06 (C_5_H_8_O^−^/C_3_H_6_N_3_^−^), 86.03 (C_3_H_4_NO_2_^−^), 88.03 (C_7_H_4_^−^), 89.05 (C_4_H_9_O_2_^−^), 99.03 (C_3_H_3_N_2_O_2_^−^),100.02 (C_3_H_4_N_2_O_2_^−^), 104.04 (C_3_H_6_NO_3_^−^), 105.04 (C_3_H_7_NO_3_^−^), 107.07 (C_3_H_9_NO_3_^−^), 108.04 (C_5_H_4_N_2_O^−^/C_2_H_6_NO_4_^−^), 130.99 (C_4_H_3_O_5_^−^), and 141.10 (C_8_H_13_O_2_^−^).

The Gα(2) pattern in TA2 (Figure 7) is characterized by displaying a high intensity in both the glassy and fibrous layers (Figure 2), where it appears as a cluster of nodular structures (Figure 7). They exhibit a diverse set of masses like *m/z* 92.03 (C_2_H_6_NO_3_^−^), 93.05 (C_2_H_7_NO_3_^−^), 99.02 (C_3_H_3_N_2_O_2_^−^), 111.03 (C_5_H_5_NO_2_^−^), 117.04 (C_4_H_7_NO_3_^−^), 119.05 (C_4_H_9_NO_3_^−^), 133.08 (C_5_H_11_NO_3_^−^), 135.06 (C_5_H_11_O_4_^−^), 143.06 (C_9_H_7_N_2_^−^/C_6_H_9_NO_3_^−^), 163.08 (C_10_H_11_O_2_^−^), 165.05 (C_9_H_9_O_3_^−^), 183.02 (C_7_H_14_PO_4_^−^), 211.10 (C_14_H_13_NO^−^), 212.10 (C_14_H_14_NO^−^), 221.09 (C_12_H_13_O_4_^−^), 223.04 (C_14_H_7_O_3_^−^), 227.12 (C_15_H_17_NO^−^), 237.10 (C_16_H_13_O_2_^−^/C_13_H_17_O_4_^−^), 238.10 (C_16_H_14_O_2_^−^), 239.10 (C_16_H_17_NO^−^), 272.11 (C_16_H_21_NO_2_S^−^/C_14_H_14_N_3_O_3_^−^), 297.07 (C_13_H_13_O_8_^−^), 298.07 (C_13_H_14_O_8_^−^), 349.25 (C_21_H_33_O_4_^−^), and 377.28 (C_23_H_37_O_4_^−^).

*Group β(Gβ).* It is defined by a higher intensity in both microlaminated and brecciated layers, where it shows in the subangular clast matrix (Figure 2 and Figure 3; Supplementary Table S2). Gβ comes with a set of inorganic cations like 55.93 (Fe^+^), 56.96 (CaOH^+^), 64.97 (SO_2_H^+^), 99.94 (CaCO_3_^+^), 144.86 (Fe_2_O_2_H^+^), 258.76 (H_3_O_4_Ti_4_^+^), 402.61 (S-Fe-unknown positive fragment), and 431.55 (Fe_6_O_6_^+^/Fe_6_SO_4_^+^), which strongly support that the main mineralization process occurring in the microlaminar layers of TA1 and TA2.

In TA1, Gβ is featured as the cations at *m/z* 43.02, 45.04, 47.01, 57.04, 59.05, 60.02, 61.03, 69.04, 71.01, 73.03, 81.04, 85.03, 87.05, 97.03, 101.03, 150.12, and 202.24 (Figure 5a), corresponding to C_2_H_3_O^+^, C_2_H_5_O^+^, CH_3_O_2_^+^, C_3_H_5_O^+^, C_3_H_7_O^+^, C_2_H_4_O_2_^+^, C_2_H_5_O_2_^+^, C_4_H_5_O^+^, C_3_H_3_O_2_^+^, C_3_H_5_O_2_^+^, C_5_H_5_O^+^, C_4_H_5_O_2_^+^, C_4_H_5_O_2_^+^, C_4_H_7_O_2_^+^, C_5_H_5_O_2_^+^, C_3_H_5_N_2_O_2_^+^/C_8_H_5_^+^, C_10_H_14_O^+^/C_10_H_16_N^+^, and C_12_H_30_N_2_^+^/C_13_H_32_N^+^. Negative fragments are found at 45.00, 53.01, 55.02, 58.01, 59.02, 69.00, 69.04, 83.02, 84.02, 85.04, 86.01, 87.01, 99.02, 101.03, 111.02, 113.04, 116.03, and 125.04, which are assigned to CHO_2_^−^, C_3_HO^−^, C_3_H_3_O^−^, C_2_H_2_O_2_^−^, C_2_H_3_O_2_^−^, C_3_HO_2_^−^, C_4_H_5_O^−^, C_4_H_3_O_2_^−^, C_4_H_4_O_2_^−^, C_4_H_5_O_2_^−^, C_3_H_2_O_3_^−^, C_3_H_3_O_3_^−^, C_4_H_3_O_3_^−^, C_4_H_5_O_3_^−^, C_5_H_3_O_3_^−^, C_5_H_5_O_3_^−^, C_8_H_4_O^−^/C_5_H_8_O_3_^−^, and C_6_H_5_O_3_^−^ (Figure 5b). The cation and anion sets suggest several nodular and microstructures (>80 micron-size), whose direction crosses the main crack infilling orientation (Figure 5).

A similar set of cations shows a higher intensity in the TA2 microlaminar layer. They correspond with 29.04 (C_2_H_5_^+^), 31.02 (CH_3_O^+^), 41.00 (C_2_HO^+^), 43.02 (C_2_H_3_O^+^), 45.04 (C_2_H_5_O^+^), 53.04 (C_4_H_5_^+^), and 125.00 (C_2_H_6_PO_4_^+^). In addition, some unknown peaks at 461.58, 474.55, and 492.59 suggest the occurrence of lipidic compounds associated with the mineralized microlaminar layer (Figure 6).

When comparing the cation with the anion TA2 distribution it is possible to recognize three different Gβ pattern variations (Figure 6 and Figure 7), which are mainly characterized through the distribution of the distinct Fe-, S-, and P-bearing negative fragments. Exactly, Gβ(1) is characterized by the occurrence of different SO_n_^−^_(4≤n≤5)_-organic adducts Gβ(2) through the Fe- and S-bearing fragments (e.g., Fe^+^, FeO_2_^−^, FeSO_5_H^−^, etc), and Gβ(3) is defined by a high concentration of P-bearing negative ions. The pattern Gβ(1) shows high concentration in the negative fragments (Figure 7) like 265.15, 279.17, 280.17, 293.18, 309.17, and 337.20, corresponding to C_12_H_25_SO_4_^−^, C_13_H_27_SO_4_^−^, C_13_H_28_SO_4_^−^, C_14_H_29_SO_4_^−^, C_14_H_29_SO_5_^−^, and C_16_H_33_SO_5_^−^. Although SO_2_^−^ and SO_3_^−^ show a high intensity in both glassy and microlaminar TA2 layers (Figure 2 and Figure 3b), the different S-bearing organic anions are found in the intermediate layer, where they display a high intensity with a 10-micron thick straight band. These fragments also determine various small circular microstructures occurring in both glassy and microlaminated layers (Figure 7), some of which show identical circular structures defined by NO_2_^−^. Interestingly, *m*/*z* 131.00 would tentatively correspond to C_4_H_3_O_5_^−^ following similar distribution shown by NO_2_^−^ in TA2.

The pattern Gβ(2) is characterized by negative ions occurring in the microlaminated layer and delimiting a couple of nodular structures found in the fibrous layer (Figure 3b and Figure 7). The most intense negative fragments are 88.93, 168.88, and 250.84, which fit well with the Fe- and S-bearing negative ions like FeO_2_H^−^, FeSO_5_H^−^, and CH_7_Fe_3_O_4_^−^, respectively. Some *m/z* 24.00, 41.00, 50.02, 51.02, 189.00, 203.01, 387.21, 411.24, 421.22, 465.61, 473.28, 479.33, 481.32, and 735.44, also follow the pattern Gβ(2) at a lower intensity. They match negative ions like C_2_^−^, C_2_HO^−^, CH_6_S^−^/C_4_H_2_^−^, C_4_H_3_^−^, C_6_H_5_O_7_^−^, C_7_H_7_O_7_^−^, C_23_H_31_O_5_^−^/C_26_H_27_O_3_^−^, C_23_H_39_O_6_^−^/ C_26_H_35_O_4_^−^, C_23_H_33_O_7_^−^, C_5_Fe_5_O_4_P_2_^−^, C_24_H_41_O_9_^−^/C_31_H_37_O_4_^−^, C_27_H_45_O_7_^−^/C_31_H_45_O_4_^−^, C_31_H_45_O_5_^−^/C_27_H_45_O_8_^−^, and C_51_H_59_O_4_^−^/C_44_H_63_O_9_^−^/C_47_H_59_O_7_^−^.

*Group γ (Gγ).* It is only characterized in TA2 where it is defined by *m*/*z* 18.04, 58.07, 78.08, 161.10, 214.24, and 242.28. The cation distribution traces several 80 micron-long egg-shaped units that are found inside the fibrous layer (Figure 2 and Figure 6). They are tentatively assigned to NH_4_^+^, C_3_H_8_N^+^, C_4_H_10_N^+^, C_6_H_15_N_3_O_2_^+^, C_14_H_32_N^+^, and C_16_H_36_N^+^, where the last two adducts may correspond to the ionization of C_14_H_28_ and C_16_H_32_ by NH_4_^+^.

*Group ε (Gε).* It only occurs in TA1 by a unique positive fragment as 70.07 (C_5_H_10_^+^). It outlines 40 to 50-micron sized elongated units with angular terminations that are obliquely oriented to the main fabric orientation (Figure 5). It overlays some of the clastic particles depleted in sulfate and silica and in the brecciated layer matrix. The G**ε** cation C_5_H_10_^+^ is also found in the fibrous patches occurring in the cryptocrystalline and fibrous layer where it concurs with Mg^+^.

*Group δ (Gδ).* It only found in TA2 (Figure 6) exhibited by the *m*/*z* 88.08 (C_4_H_10_NO^+^). Its distribution appears as different elongated nodular microstructures spreading throughout the different layers, whose orientation is oblique to the principal direction of the TA2 fabric (Figure 2 and Figure 6). The fragment at 88.08 partially occurs in the couple of nodular units that define the Group γ.

*Group φ (Gφ).* It occurs only in TA1 by the positive ions (Figure 3a) at 22.99 (Na^+^), 38.97 (K^+^), 40.97 (H_2_K^+^), and 94.93 (K_2_OH^+^/P_2_HS^+^). It is also traced by negative fragments at 62.97 (PO_2_^−^), 78.97 (PO_3_^−^), 118.95 (NaPO_4_H^−^), 138.94 (CHO_4_P_2_^−^), 139.94 (CH_2_O_4_P_2_^−^), 155.93 (CH_3_O_3_P_3_^−^), 365.25 (C_18_H_38_PO_5_^−^/C_17_H_34_PO_6_^−^), 425.23 (C_19_H_38_PO_8_/C_26_H_33_O_5_^−^), 437.17 (C_16_H_29_N_4_O_8_S^−^/C_24_H_25_N_2_O_6_^−^), 568.65 (C_35_H_76_N_4_O^−^/C_36_H_76_N_2_O_2_^−^), 628.64 (C_42_H_78_NO_2_^−^), 670.57 (C_41_H_77_NO_3_K^−^/C_40_H_80_NO_6_^−^), and 730.56 (C_41_H_81_NO_7_P^−^), which likely correspond with different adducts and fragments of ceramides and glycerophospholipids. This group also come with TA1 unknown negative fragments found at 322.83, 466.71, 467.71, 495.65, 525.65, 526.70, 528.69, 768.16, and 772.03. They might correspond with different adducts of non-organic phosphatic anions, and heterocyclic structures.

In Ta2, Gφ is mainly defined by negative ions occurring in the microlaminated layer followed by a 40-micron thick discontinuous band separated into three different units that limit the fibrous layer (Figure 7). However, as in TA1, there are some cations like K^+^ (Figure 3b) that show a high intensity in Gφ. The negative peaks are found at *m/z* 58.97,62.96, 78.96, 79.96, 93.98, 95.95, 118.94, 138.93, 178.91, and 180.92 matching major P- and S-bearing anions as CPO^−^, PO_2_^−^, SO_2_^−^, PO_3_^−^, SO_3_^−^, CH_3_PO_3_^−^, SO_4_^−^, HNaPO_4_^−^, HAlPO_5_^−^, HAlCaPO_5_^−^, and NaP_2_O_6_^−^. Such pattern is also followed by larger negative fragments as 424.75, 568.65, 628.63, 670.56, and 772.47 that may correspond with K_2_P_5_O_12_^−^, C_37_H_80_N_2_O^−^, C_42_H_78_NO_2_^−^, C_38_H_72_NO_8_^−^ /C_38_H_73_NO_6_P^−^, and C_43_H_67_NO_9_P^−^, respectively.

*Group κ (Gκ).* The occurrence of large positive fragments at 494.57, 522.60, and 550.62 corresponds with N-bearing ions like C_34_H_72_N^+^, C_36_H_76_N^+^, and C_38_H_80_N^+^. They display two thick laminae (50-micron) crossing over the glassy and microlaminated TA2 layers (Figure 6; Supplementary Figure S3). The glassy layer band occurs in the interior of the layer itself; the second band is emplaced along the boundary between the crystalline and microlaminated layers. Interestingly, it directly contacts with a 40-micron thick lamina defined by *m*/*z* 307.31, 309.33, and 360.36, which possibly correspond with C_21_H_39_O^+^, C_21_H_41_O^+^, and C_25_H_44_O^+^. The N-bearing cations might be NH_4_^+^-organic adducts of hydrocarbons like C_34_H_68_ (C_34:1_), C_36_H_72_ (C_36:1_), and C_38_H_76_ (C_38:1_).

*Group ω (Gω).* Different set of molecular fragments that define an isolated nodular structure occur in the TA2 glassy layer (Figure 6 and Figure 7). Gω is traced by a set of positive fragments as 79.02, 495.45, 523.49, 551.52, and 579.56, which correspond with a series of diacylglycerides including C_5_H_3_O^+^, C_31_H_59_O_4_^+^ (DAG_28:0_), C_33_H_63_O_4_^+^ (DAG30:0), C_35_H_67_O_4_^+^ (DAG32:0), and C_37_H_71_O_4_^+^ (DAG34:0). The negative fragments defining Group ω occur at *m/z* 127.09, 155.12, 157.14, 169.13, 197.17, 199.17, 227.21, 241.22, 253.23, 255.24, 269.25, 281.26, and 283.28, corresponding to fatty acid anions like C_7_H_11_O_2_^−^ (C_7:1_), C_9_H_15_O_2_^−^ (C_9:1_), C_9_H_17_O_2_^−^ (C_9:0_), C_10_H_17_O_2_^−^ (C_10:1_), C_12_H_21_O_2_^−^ (C_12:1_), C_12_H_23_O_2_^−^ (C_12:0_), C_14_H_27_O_2_^−^ (C_14:1_), C_15_H_29_O_2_^−^ (C_15:0_), C_16_H_29_O_2_^−^ (C_16:1_), C_16_H_31_O_2_^−^ (C_16:0_), C_17_H_33_O_2_^−^ (C_17:0_), C_18_H_33_O_2_^−^ (C_18:1_), and C_18_H_35_O_2_^−^ (C_18:0_), respectively.

*Group ο (Gο).* Different negative ions like 19.00 (F^−^), 32.98 (HS^−^), and 34.97 (Cl^−^) occur in TA1 and TA2. They follow different patterns that are not observed in the morphological groups described before. The fluoride and chloride anions (Figure 5b and Figure 7) exhibit rounded microstructures (10 to 20 micron-size), whose distribution is nonconcurrent. This suggests that F^−^ and Cl^−^ have likely followed different biological and/or environmental subsurface processes. Although the distribution of HS^−^ is following part of Gβ(3) and Gγ intensity patterns, it is not found in the areas where SO^−^_n(2≤n≤4)_ ions are occurring (Figure 5b and Figure 7). Therefore, the HS^−^ distribution pattern suggests that it may result from biological or non-biological processes, which are different from those involved in the SO^−^_n(2≤n≤4)_ ions.

### 4.2. ToF-SIMS Spectral Identification

#### 4.2.1. Hydrocarbons

Alkane fragments occur as short-chained hydrocarbon ions like C_3_H_7_^+^ (43.05), C_4_H_9_^+^ (57.07), C_5_H_11_^+^ (71.09), and C_6_H_13_^+^ (85.1). They show an intensity ranging between 150 and 70,000 cps, which drop to negligible for *m/z* higher than 99.13 Da (C_7_H_15_^+^). The fragments of linear monounsaturated alkanes show an identical pattern, where the short-chain fragments like C_3_H_5_^+^ (41.04), C_4_H_7_^+^ (55.05), C_5_H_9_^+^ (67.09), C_6_H_11_^+^ (83.09), and C_7_H_13_^+^ (97.11) have strong peaks with an intensity ranging between 2,000 and 100,000 cps. The peak intensity drops drastically for mass higher than 111.12 Da (C_8_H_15_^+^). The TA1 and TA2 occurrences of short-chained fragments suggest that the main hydrocarbon source is the secondary fragmentation of linear hydrocarbon branches normally occurring in large lipidic structures, but not from isolated hydrocarbons that result from lipid degradation. The total intensity of the monounsaturated fragments is higher than that of the saturated positive ions (Supplementary Figure S4a), suggesting that monounsaturated hydrocarbon backbones are more abundant than saturated alkyl structures. Additionally, the observation of two high-intensity peaks (I > 15,000 cps) at *m*/*z* 43.06 (C_3_H_7_^+^) and 57.07 (C_4_H_9_^+^) (Supplementary Figure S4b) with different distribution suggest the occurrence of branched hydrocarbons or isoprenoids that either have ethyl or propyl branching. Interestingly, C_4_H_9_^+^ displays a very low intensity in the microlaminar layer, whereas it is high in the glassy and fibrous-cryptic layers (Supplementary Figure S4b). Such a disparate distribution suggests that some of the hydrocarbon backbones, which have been preserved inside those layers, could be propyl-branched structures.

The occurrence of larger hydrocarbon structures could also be followed by the distribution of NH_4_^+^-bearing adducts (Supplementary Figure S3 and Table S3). Such a set of positive ions are found at *m*/*z* 200.24 (C_13_H_30_N^+^), 214.25 (C_14_H_32_N^+^), 242.28 (C_16_H_36_N^+^), 270.32 (C_18_H_40_N^+^), 298.35 (C_20_H_44_N^+^), 312.36 (C_21_H_46_N^+^), 326.38 (C_22_H_48_N^+^), 368.42 (C_25_H_54_N^+^), 494.56 (C_34_H_72_N^+^), 522.60 (C_36_H_76_N^+^), 536.59 (C_37_H_78_N^+^), and 550.62 (C_38_H_80_N^+^). They correspond to monounsaturated hydrocarbons like C_13:1_, C_14:1_, C_18:1_, C_20:1_, C_21:1_, C_22:1_, C_25:1_, C_34:1_, C_36:1_, C_37:1_, and C_38:1_, which could have been sourced from different lipidic molecules. As the peak intensity in ≤C_25:1_ is higher than in ≥C_34:1_ monounsaturated alkene, which suggests that the main sources of linear hydrocarbons are glycerolipids, with ceramides being a secondary origin.

#### 4.2.2. Polycyclic Aromatic Hydrocarbons (PAHs)

The ToF-SIMS of sample BH8-24c has provided a wide diversity of cation masses that are attributed to the fragmentation of different PAHs (Supplementary Table S4) at 115.05, 128.06, 141.07, 152.06, 165.06, 178.07, 189.06, 202.07, 215.08, 226.07, 239.07. They match well with major positive fragments and molecular masses like C_9_H_7_^+^, C_10_H_8_^+^, C_11_H_9_^+^, C_12_H_8_^+^, C_13_H_9_^+^, C_14_H_10_^+^, C_15_H_9_^+^, C_16_H_10_^+^, C_17_H_11_^+^, C_18_H_10_^+^, and C_19_H_11_^+^; which might come from polyaromatic structures including derivatives of indene (C_9_H_8_), acenaphthylene (C_12_H_8_), fluorene (C_13_H_10_), pyrene (C_16_H_10_), or cyclopentachrysene (C_18_H_10_).

The PAHs show a high distribution and abundance throughout TA1 and TA2. Its occurrence in a deeper area of the basement could result from the combination of different bacterial by-products, the degradation of terpenoid, and the transportation from shallower areas due to their stability. In this regard, fungal and bacterial degradation of plant tissues should be expected in the soil, where organic mineralization is produced by different ways [32,33]. They could be transported by downwelling meteoric fluids that would add to the PAHs in the underground reservoir, where they may be also produced by heterotrophic microbes.

#### 4.2.3. Fatty Acids (FAs)

**Figure 8:**
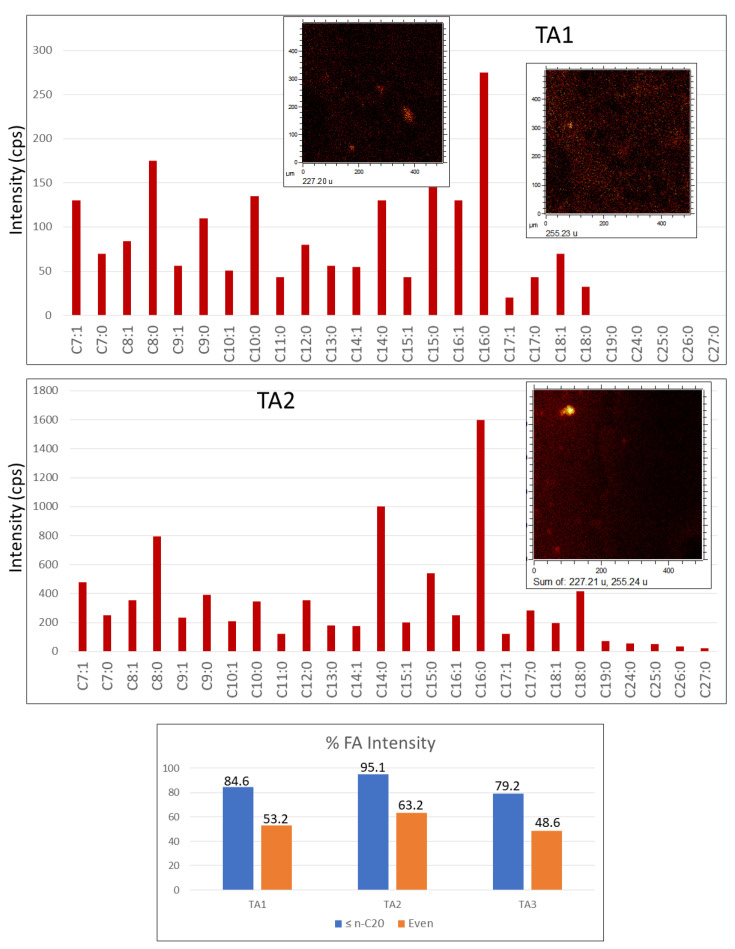
Bar diagrams plotting the mass distribution of saturated and monounsaturated fatty acids in TA1 and TA2. The lower diagram draws a high abundance for ≤n-C20, moderate for even FA chains supporting a microbial origin for the organic compounds. Diagram units are expressed as count per second (cps) intensity unit. The ToF-SIMS images were obtained from a rasterized surface area of a 500 μm square.

The spectral analysis recognizes a diverse set of [M-H]^−^ and [M]^−^ ions from 127.09 to 305.25 Da (Figure 8; Supplementary Figure S5; Supplementary Table S5) with maximum peaks at 227.20, and 255.23 corresponding to myristic (C_14_H_28_O_2_), and palmitic (C_16_H_32_O_2_) acids. Although the fatty acid distribution is similar in the three TA1, TA2, and TA3, the FAs intensity is one order of magnitude higher in TA2 suggesting a greater biomass preservation. The FAs distribution is widely spread throughout the whole surface associated to the Fe- and S-bearing ions (e.g., FeO_2_^−^, Fe_2_H_5_O_2_^−^, and FeSO_5_H^−^) except in some nodular microstructures where the mass intensity is maximal (Supplementary Figure S5a–c). In this case, they co-occur with other ions like NO_3_^−^ and SO^−^_n(2≤n≤4)_. In TA1, FAs show a high concentration in various elements (Supplementary Figure S5a) like spheroidal (*m*/*z* 127.08, 157.12, and 169.12), ovoidal and circular Gα microstructures (*m*/*z* 227.20), and Gα linear elements (*m*/*z* 143.11). The FAs distribution in TA2 (Figure 7; Supplementary Figure S5b) is nearly restricted to a nodular structure that characterizes Gω, meanwhile they also show a secondary intensity in the glassy layer in which the Gα pattern concurs. Furthermore, TA3 FAs show two distinctive distribution patterns corresponding with the massive occurrence in the Fe- and S-bearing ion layers (Supplementary Figure S5c) including most of the [M − H]^−^ peaks. With their spheric microstructures (*m*/*z* 143.11, and 157.12), they are also defined by maximum concentration in PO^−^_n(2≤n≤5)_ ions. The FAs occurrence in TA2 and TA3 correlates well with the distribution of ions, which is tentatively assigned to diacylglycerides and glycerophospholipids.

The [M − H]^−^ anion distribution shows that the saturated and monounsaturated compounds are FAs (≤n-C_20_), and the total intensity of FAs with even carbon chains exceeds their odd counterparts in TA1 and TA2 (Figure 8). On the other hand, the saturated and monounsaturated FAs longer than 19 carbon atoms have a negligible concentration. The FAs mass distribution strongly supports a microbial origin for the organics in Peña de Hierro basement [34].

#### 4.2.4. Acylglycerols

The detections of *m*/*z* 495.45, 523.49, 551.52, and 579.56 correspond with C_31_H_59_O_4_^+^ (DAG(28:0)), C_33_H_63_O_4_^+^ (DAG(30:0)), C_35_H_67_O_4_^+^ (DAG(32:0)), and C_37_H_71_O_4_^+^ (DAG(34:0)) (Supplementary Figure S6, and Table S6). Such cation sets suggest the preservation of [M + H − OH]^+^ ions of diacylglycerides [35]. The TA2 acylglycerol distribution fully correlates with the distribution of FAs (≤C_18:0_) that defines the Group ω (Figure 6 and Figure 7), which indicates that both FAs and acylglycerol fragments originate from the same microbial source. Although the mass distribution agrees with the occurrence of diacylglycerides, it could also correspond with the presence of triacylglycerides. However, it cannot be confirmed as the peak intensity above 800 Da lies under the spectra background.

#### 4.2.5. Sphingolipids and Phospholipids

TA1 and TA2 have a diverse set of even peaks (Supplementary Table S6) at 282.29 (C_18_H_36_NO^+^), 284.30 (C_18_H_38_NO^+^), 308.29 (C_20_H_38_NO^+^), 310.32 (C_20_H_40_NO^+^), 564.56 (C_37_H_74_NO_2_^+^), 594.56 (C_37_H_72_NO_4_^+^), and 668.60 (C_41_H_82_NO_5_^+^) that are found as result of the ceramide fragmentation [36]. They come with the presence of phosphocholine fragments 104.11 (C_5_H_14_NO^+^), and 184.08 (C_5_H_15_PNO_4_^+^) (Supplementary Figures S3 and S7), which are the major ions from different phospholipids and sphingomyelins [37]. They could be the case of several peaks associated with TA1 Gφ, TA2 Gβ(3), and TA3 micronodular structures (Figure 5b and Figure 7; Supplementary Figure S8a–c) like *m/z* 526.69, 568.65, 628.63, 670.56, and 772.47. Although the first three peaks are difficult to characterize, they could correspond either to phosphatic salt, phospholipid or sphingolipid adducts with *m/z* 670.56, and 772.45. Such peaks can be tentatively assigned to [M − H_2_O − H]^−^ like C_38_H_73_NO_6_P^−^ (e.g., PE(O-16:0/17:1), and C_43_H_67_NO_9_P^−^ (e.g., PS(17:2/20:5)), which are the same peaks detected by ToF-SIMS for the Peña de Hierro gossan [37,38]. Furthermore, the TA2 peak at 772.03 has been reported as a [M − 2H]^2−^ in the characterization of large phospholipids [39]. However, SIMS rarely, if ever, produces double or triple charged ions, so it could be an unknown complex lipidic structure (e.g., polyketide) or PO_3_-bearing adduct of some lipid preserved inside the mineral-rich phosphatic matrix.

#### 4.2.6. Peptide Fragments and Amino Acids

Various positive and negative ions corresponding to different N-bearing fragments match the fragmentation of amino acids and peptides reported from previous ToF-SIMS analyses [40,41,42,43]. Such a set of fragments has been mainly found as TA1 and TA2 positive ions (Gα) (Figure 5a and Figure 6; Supplementary Table S7) at *m/z* 28.02, 30.04, 42.04, 44.05, 54.04, 56.06, 68.03, 70.04, 71.06, 72.05, 73.05, 73.06, 83.06, 84.08, 86.10, 100.08, 107.06, 109.08, 113.08, 115.06, 120.09, 122.08, 123.10, 124.10, 125.12, 126.11, 135.09, 136.09, 137.11,138.11, 145.10, 147.08, and 159.12; which are in line with ions CH_2_N^+^, CH_4_N^+^, C_2_H_4_N^+^, C_2_H_6_N^+^, C_3_H_4_N^+^, C_3_H_6_N^+^, C_2_H_2_N_3_^+^, C_2_H_4_N_3_^+^, C_3_H_7_N_2_^+^, C_2_H_6_N_3_^+^, C_3_H_7_NO^+^, C_2_H_7_N_3_^+^/C_4_H_9_O^+^ (73.06), C_4_H_7_N_2_^+^, C_5_H_10_N^+^, C_5_H_12_N^+^, C_4_H_10_N_3_^+^, C_6_H_7_N_2_^+^, C_6_H_9_N_2_^+^, C_6_H_11_NO^+^, C_4_H_7_N_2_O_2_^+^, C_8_H_10_N^+^, C_6_H_8_N_3_^+^, C_8_H_13_N^+^, C_7_H_12_N_2_^+^, C_7_H_13_N_2_^+^, C_8_H_11_N_2_^+^, C_8_H_12_N_2_^+^, C_8_H_13_N_2_^+^, C_7_H_12_N_3_^+^, C_6_H_13_N_2_O_2_^+^, C_8_H_9_N_3_^+^/C_5_H_11_N_2_O_3_^+^ and C_7_H_15_N_2_O_2_^+^ (Supplementary Table S7). They also come together with anion peaks occurring in TA1 Gα and Gβ (Figure 5a), and, in TA2 (Gα(2)) (Figure 7) at 49.01, 65.02, 90.01, 91.03, 92.04, 93.05, 99.02, 111.03, 117.04, 119.06, 133.08, 143.06, 179.05 and 272.11 (Supplementary Table S7), where they show a much higher intensity. Such a set of masses can tentatively be paired with several N-bearing anions like C_4_H^−^, C_3_HN_2_^−^, C_4_N_3_^−^, C_5_H_3_N_2_^−^, C_5_H_4_N_2_^−^, C_5_H_5_N_2_^−^, C_3_H_3_N_2_O_2_^−^, C_4_H_7_NO_3_^−^, C_4_H_9_NO_3_^−^, C_8_H_9_N_2_^−^, C_7_H_7_N_2_O^−^, C_9_H_7_N_2_^−^, C_5_H_11_N_2_O_3_S^−^, and C_13_H_27_N_2_O_3_S^−^. In TA2 Gγ, a set of additional cationic peaks could potentially correspond to additional peptidic fragments (Supplementary Table S7) like 18.04, 58.07, 72.08, and 161.10 matching NH_4_^+^, C_3_H_8_N^+^, C_4_H_10_N^+^, and C_9_H_11_N_3_^+^.

As it has been mentioned, the occurrence of positive and negative ions in TA1 and TA2 suggests the presence of protein fragments (e.g., C_3_H_8_N^+^, C_5_H_12_N^+^, and C_8_H_10_N^+^, see Siljeström et al. [40]) and amino acids like lysine, arginine, cysteine, glycine, or aspargarine [31,41,42]. The distribution of amino acid-derived ions that is alternating among microsites (e.g., Gα and Gβ versus Gγ) suggests the different microbial sources for peptides.

#### 4.2.7. Hopanoids and Steroids

TA1 and TA2 fragments (Supplementary Table S8) including C_35_H_64_NO_3_^+^ (546.48), and C_35_H_64_NO_4_^+^ (562.49) correspond to the formation of [M + H]^+^ adducts for aminobacteriohopanetriol (C_35_H_63_NO_3_) and aminobacteriohopanetetrol (C_35_H_63_NO_4_) [44,45]. In addition, some other cation peaks like 177.16 (C_13_H_21_^+^), 219.21 (C_15_H_23_O^+^), 388.39 (C_27_H_50_N^+^ as an NH_4_^+^ adduct of C_27_H_46_^+^) and 561.48 (C_36_H_65_O_4_^+^) occurring as major to minor peaks (I < 500 to 50 cps) suggest the presence of additional bacteriohopanepolyols that could appear as secondary minor lipidic structures [46]. The hopanol cation distribution follows the arrangement of the Fe-bearing ions that includes Gα in TA1 (Figure 3a and Figure 5a,b; Supplementary Figure S9a) and several TA2 groups like Gγ, Gω, and Gβ(2) (Figure 3b, Figure 6 and Figure 7).

The absence of the peaks assigned to sterol derivatives found in the Upper Gossan deposits (e.g., C_27_H_46_O^+^, and C_27_H_45_SO_4_^−^) [14] suggests that steroids occur either as a minoritarian component or are fully absent in sample BH8-24c. In this regard, some few peaks observed at 386.39 (C_28_H_50_^+^/C_27_H_46_O^+^), and 388.39 (C_27_H_50_N^+^/ C_28_H_52_^+^/C_27_H_48_O^+^) could suggest the presence of C_27_ and/or C_28_ steroids (e.g., cholestanol derivatives) in deeper areas of the Peña de Hierro gossan.

#### 4.2.8. Other Compounds

In multiple cases, the spectral distribution of anion peaks has not been assigned to the molecular formula and/or fragmentation pattern of any compound. This is the case of a Gβ(2) anion set of TA2 like 24.00, 41.00, 50.02, and 51.02 that correspond with C_2_^−^, C_2_HO^−^, CH_6_S^−^/C_4_H_2_^−^, and C_4_H_3_^−^. They concur with a series of *m*/*z* 173.00, 189.00, 203.01, 387.21, 411.24, 421.23, 473.28, 481.32, and 735.44. The mass distribution could be tentatively characterized as one or several compounds bearing heterocyclic structures like the following: (1) saccharides tentatively identified through *m*/*z* 173.00, 189.00 and 203.01, corresponding to C_6_H_5_O_6_^−^, C_6_H_5_O_7_^−^, and C_7_H_7_O_7_^−^; (2) glycerophospholipids characterized by *m*/*z* 735.44 like C_37_H_68_PO_12_^−^, while 189.00 and 203.01 could be fragments of the phospholipid saccharide head; (3) different fragments of unknown heterocyclic structures like *m*/*z* 387.21, 411.24, 421.23, 473.28, 481.32, and 735.44 corresponding with C_27_H_45_O_7_^−^, C_22_H_35_O_7_^−^, C_23_H_33_O_7_^−^/C_20_H_37_O_9_^−^, C_24_H_41_O_9_^−^, C_24_H_49_O_9_^−^, and C_39_H_62_N_2_O_11_^−^; and (4) fragments tentatively assigned to terpenoids (e.g., hopanols or other triterpenes) like *m*/*z* 411.24, 421.23, 473.28, and 481.32 that could correspond with C_26_H_35_O_4_^−^, C_27_H_33_O_4_^−^, C_34_H_33_O_2_^−^ and C_34_H_41_O_2_^−^.

Other anion peaks could also suggest the presence of potential polyketides and terpenoids following different TA1 and TA2 patterns. This is the case of *m/z* 425.23 and *m/z* 437.17 (Supplementary Figure S9b) that tentatively correspond with C_26_H_33_O_5_^−^, and C_16_H_29_N_4_O_8_S^−^/C_24_H_25_N_2_O_6_^−^. Similar compounds have been associated with bacterial quinones (e.g., hydroxylated tridentoquinone) [47], and polyketides or complex terpenoids which already described in the Rio Tinto subsurface [10].

### 4.3. Principal Component Analysis (PCA) of ToF-SIMS Images

The ToF-SIMS data PCA has provided an additional layer of information including the variability of the molecular composition in the interior of some microstructures. Such analysis can provide more information to identify microbial groups that have been potentially preserved in the sample. We have performed the PCA for different molecular classes that have included major anions, amino acids, N-bearing lipid cations, FA anions, acylglyceride cations, and phospholipid anions. However, the ion dataset that has revealed extra information in terms of molecular variability originating from the PCA, which was performed for the major ions, amino acid ion fragments, and FA adducts.

#### 4.3.1. Major Anions

**Figure 9:**
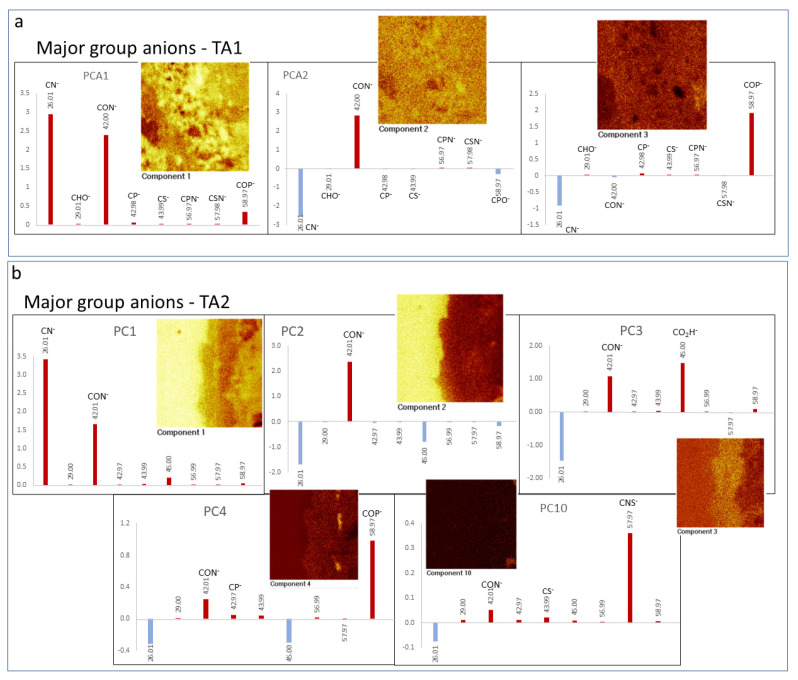
Principal component analysis (PCA) performed for the ToF-SIMS data of the major group ions like CN^−^, CON^−^, CP^−^, COP^−^, CS^−^, and CSN^−^ in both TA1 (**a**) and TA2 (**b**) areas. The PCA of TA1 considered 8 masses, while the number of masses were 9 for TA2. The ToF-SIMS images were obtained from a rasterized surface area of a 500 μm square.

The PCA of the spectral data of major ions such as CN^−^ (26.00), CON^−^ (42.00), CS^−^ (43.97), CNS^−^ (57.98), and COP^−^ (58.97) show that they have a high correlation with different microstructures found in TA1 and TA2 (Figure 9). In TA1, the PC2 image shows (Figure 9a) that CON^−^ (*m*/*z* 26.00) has a close relation with some of the nodular microelements of Gα (Figure 5). Furthermore, PC3 determines that COP^−^ (*m*/*z* 58.97) correlating with Gφ (Figure 5b), which is characterized by PO_n_^−^_(2≤n≤4)_ ions, is different adducts of phosphatic salts and these compounds are tentatively assigned to phospholipids (Figure 5b and Figure 9a). The PCA of the TA2 major ions show a high correlation with a wider number of microstructures. The PC2 shows that CON^−^ distribution correlates well with Gα, while CON^−^ and CO_2_H^−^ (*m*/*z* 45.00) in PC3 show a good correlation with Gβ(1) (Figure 6b and Figure 9b). PC4 shows that COP^−^ (*m*/*z* 58.07) and, to lesser extent, CON^−^ distribution correlates with TA2 Gφ, which is defined by the content in different inorganic and organic PO_3_-bearing ions like phospholipids. Finally, PC10 determines through the positive score of CNS^−^ (*m/z* 57.97) and, secondarily, CON^−^ follows Gβ(2), Gγ (e.g., NH_4_^+^) and Gο (e.g., HS^−^) (Figure 7 and Figure 9b), which have a great affinity with ions that correspond with amino acids.

#### 4.3.2. Amino Acid Fragments

The PCA of the TA1 cations identified as amino acid and peptide fragments results in five distinctive PCs (PC1, PC4, PC5, PC9, and PC16). The primary cation distribution (PC1) correlates the occurrence of the clasts embedded in the microbrecciated layer (Figure 2 and Figure 3a; Supplementary Figure S10; Supplementary Table S7). However, such cation arrangement shows a clear variability among other different PCs. For instance, PC1 correlates the distribution of the sulfate- and silica-bearing clastic elements, the other four PC4, PC5, PC9, and PC16 show a variability along with the different microbreccia clasts. The extreme cases are PC9 (defined through *m*/*z* 18.04, 54.04, and 115.05), and PC16 (*m*/*z* 125.12) exhibiting an inverse distribution (Supplementary Figure S10) in the sulfate-bearing clastic elements. In addition, PC4 (*m*/*z* 28.02, 54.04, and 56.06) and PC5 (*m*/*z* 56.06) also correlate with the distribution of the silica anions occurring in the microbrecciated and the fibrous-cryptocrystalline layers (Figure 2 and Figure 3a). Furthermore, a secondary cation distribution (PC2) correlates the distribution of the microlaminated layer with the distribution of *m*/*z* 42.04 (C_2_H_4_N^+^).

The PCA of the amino acid cation distribution in TA2 appears three distinctive patterns (Supplementary Figure S11), which follow Gα, Gβ, and Gγ (Figure 6). PC1 determined by *m*/*z* 42.04, 44.06, 56.06, and 73.06 shows a high correlation with Gα, which primarily tracks the glassy layer, and, secondarily, the cryptocrystalline and fibrous layer (Figure 2). The main PC1 pattern shows some changes that are defined by PC2 (*m*/*z* 56.06). PC3 defined mainly by *m*/*z* 42.04 and 44.04 correlates with Gβ(2) that is followed by the laminar microstructure. PC4 (*m*/*z* 44.06), PC13 (*m*/*z* 18.04, and 70.04), and PC15 (*m/z* 18.04, and 115.06) match the pattern Gγ that is outlined by the occurrence of nodular microstructures enriched in NH_4_^+^ and NO_2_^−^_._

#### 4.3.3. [M − H]^−^ FA Adducts

**Figure 10:**
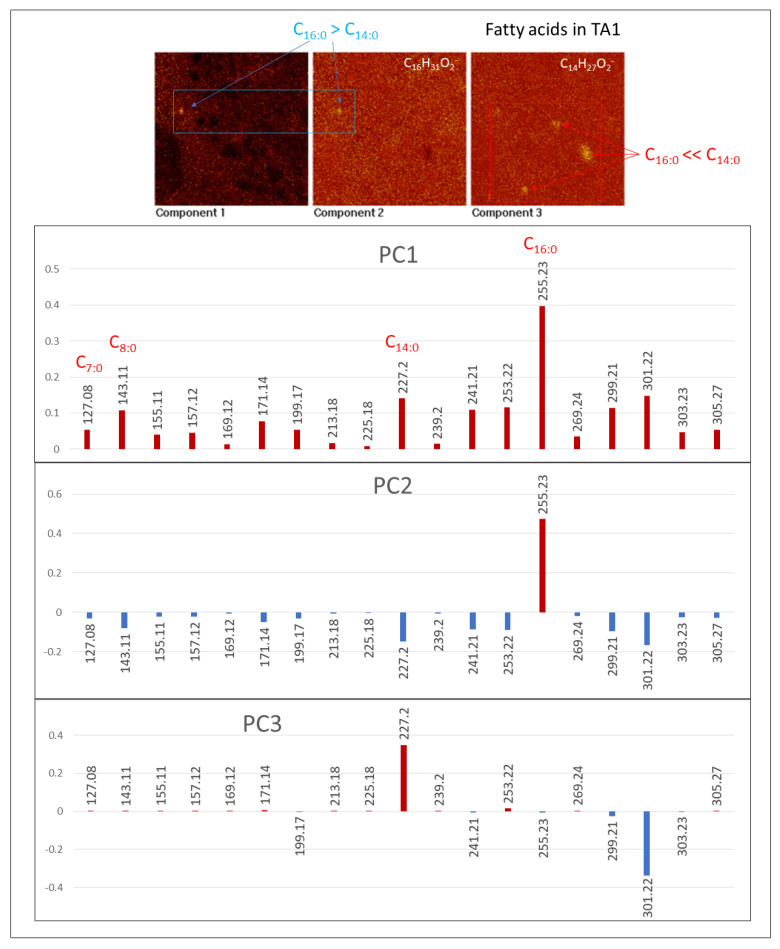
Principal component analysis (PCA) using 19 masses of FA [M − H]^−^ adducts from TA1. It can be observed a variation in the FA composition in different micronodular structures and microclasts. PC1 and PC2 show that C_16:0_ followed by C_14:0_ is the most abundant FA in TA1, particularly in the micronodular structure pointed by the blue arrows and square. However, C_14:0_ is the dominant FA in the sulfate-rich microclasts (red square and arrows) forming the microbrecciated layer (see Figure 2b). The ToF-SIMS images were obtained from a rasterized surface area of a 500 μm square.

The PCA of FAs in TA1 and TA2 produces distinctive images showing an unequivocal composition variability of some microstructures. In TA1, three PC images (PC1, PC2, and PC3) have revealed that the microstructure composition variability is mainly driven by *m*/*z* 227.20 and 255.23 corresponding to C_14:0_ and C_16:0_. PC1 image suggests that the main FA distribution in TA1 associates with Gα by following the microlaminated layer and the matrix with the brecciated microstructure layer (Figure 2, Figure 5b and Figure 10). PC2 comes from the C_16:0_ enrichment in a >20-micron size circular microstructure (Figure 10). In turn, PC3 is defined by C_14:0_, which shows a high intensity in the SO_3_-rich clastic microelements (Figure 10). PC3 is also linked to the circular microstructure recognized in PC1 and PC2, which is spotted by the high intensity of C_16:0_. Therefore, such a microstructure shows a FA variability limited by C_14:0_ and C_16:0_ (Figure 10).

**Figure 11:**
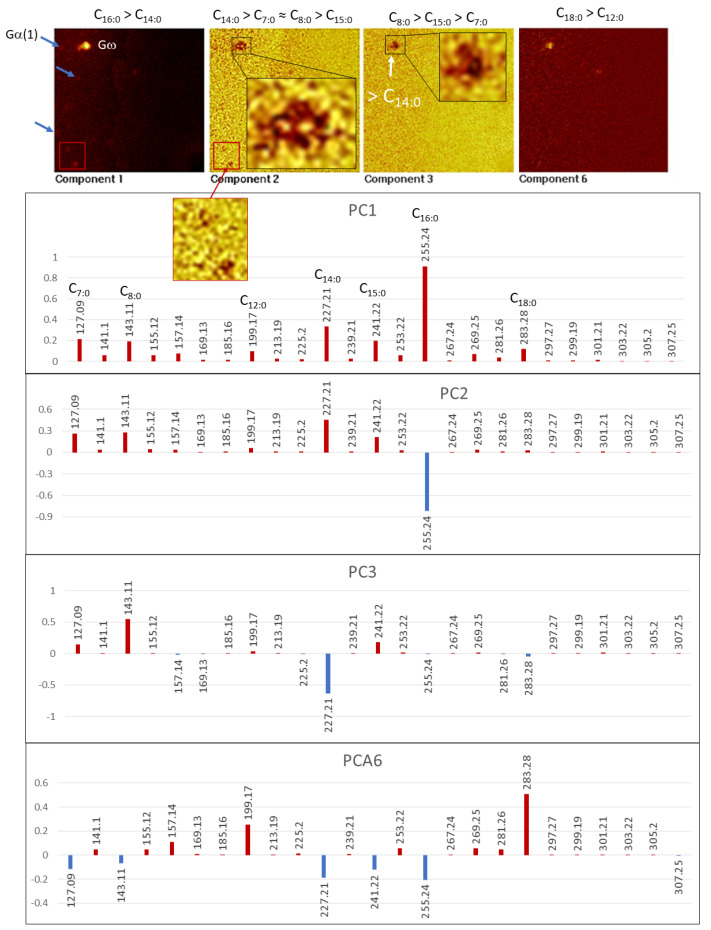
Principal component analysis (PCA) obtained through the ToF-SIMS FA data obtained in TA2. For such analysis, 25 masses identified as FA adducts were considered. As in TA1, the analysis shows (see PC1) that the primary FA in the sample is C_16:0,_ followed by C_14:0_, which are mainly found in Gω, and Gα(1). They are respectively associated with acylglyceride fragments and NO^−^_n(2≤n≤3)_ ions (blue arrows). Furthermore, such a distribution also suggests that C_14:0_ is the most abundant FA outside the Gω, and Gα(1) microstructures. PC2, PC3, and PC6 reveal a heterogeneous FA distribution inside the Gω microstructure suggesting a diverse internal composition. The red square points a couple of micronodules showing an internal variation as observed in the Gω microstructure. The ToF-SIMS images were obtained from a rasterized surface area of a 500 μm square.

The PCA for the FA adducts found in TA2 shows a composition variability driven by five compounds as C_7:0_, C_8:0_, C_14:0_, C_15:0_, and C_16:0_ (Figure 11). PC1 image is primarily constructed through the C_16:0_ intensity distribution that follows patterns Gω and Gα(1), which are defined through the acylglyceride adducts and NO_n_^−^_(2≤n≤3)_ ions, respectively. PC2 shows that Gω is split into different minute areas (<5 microns) enriched in C_14:0_ > C_7:0_ ≈ C_8:_0 > C_15:0_, which are covered by a low intensity (low correlation) zone likely represented by C_16:0_ (Figure 11).

The compositional variability of the Gω microstructure (Figure 6 and Figure 7) is also revealed by the low correlation shown by C_14:0_ in PC3 (Figure 11), suggesting that it has a complex internal configuration at a micron size scale. PC6 shows that some microstructures also have varying compositions in larger FA chains. In this regard, a distinctive circular microstructure (see PC6 in Figure 11) displays a high intensity in C_18:0_ and, secondarily, in C_12:0_, indicating a complex internal composition as well.

## 5. Discussion

The ToF-SIMS spectral and imagine analyses, which combine data from the distribution of inorganic and organic compounds with microstructures in the sample, have revealed three different information levels. These three levels include mineralization (inorganic composition), traces of microbial communities (biomolecular and organic composition), and metabolism (traces of inorganic metabolites). The integration of such information with the (paleo)environmental and geobiological complementary data [4,12,23] provides a plausible way to reveal the origin of the microbial traces resulting from different mineralization pathways. Such pathways were ultimately controlled by the long-term biooxidation process of the sulfide orebody.

### 5.1. Mineralization

The underground mineralization processes have played an essential role in the preservation of the ancient microbial activity. The main process has been driven by the iron and sulfur mobilization that has ended in forming the ferric oxysulfates laminar infillings accompanied secondarily by silica. The combination of the microstructure fabric with the inorganic composition (Figure 2, Figure 3 and Figure 4a,b) shows that three mineral groups are infilling the Carboniferous stockwork substrate [13]. The overabundance of the iron and sulfate ions supports that the main composition of the mineral matrix corresponds to sulfates and ferric oxysulfates and oxyhydroxides. The laminated microstructure and the microbrecciated microstructure matrix (Figure 2) have a high concentration in different Fe ions as Fe^+^, FeO^+^, Fe_2_O_2_^+^, FeO_2_H^−^, and FeSO_5_H^−^ (Figure 3). This supports that the matrix is composed of oxyhydroxides and oxysulfates (e.g., goethite, schwertmannite, and jarosite), which could have lately lost part of the SO_4_^−^ through diagenesis [4]. Furthermore, the occurrence of the glassy layer and the clastic microelements, which are mainly composed of SO_n_^−^_(2≤n≤5)_, accompanied by K^+^, and Na^+^ arranged as ovoidal or layered micron-sized morphologies (Figure 3), but depleted in Fe-, Ca-, Al-, and Mg-bearing ions (Figure 4). It supports the presence of acidic S-bearing compounds with enriched sulfur (e.g., native sulfur and an unidentified oxysulfate).

Several Si-bearing anions including SiO_2_^−^ and Si_2_O_4_H^−^, along with cations like Mg, Na, Ca and Al are found forming layers with fibrous and cryptocrystalline appearance (Figure 2 and Figure 3; Supplementary Figure S1). S-bearing inorganic ions and some organic ions also co-occur with the silica-rich TAs areas (Figure 3). Such distribution is also observed in the SEM-EDAX results (Supplementary Figure S1), where S and C have up to 15 wt% in the silica-rich layers. Silica ions are also found forming some of the clasts included in the brecciated microstructure (Figure 2 and Figure 3a). The silica distribution not only supports the presence of fine layers (<100 microns) composed of silica minerals, but also the occurrence of complex mineral intermixes formed by silica, sulfates, and iron oxides preserving the organic compounds (Figure 3; Supplementary Figure S1).

The alternation between the three different mineral endmembers suggests that the underground hydrochemistry and, therefore, the mineralization processes, resulted from paleoenvironmental changes controlled by pH and redox. We can envisage that the subsurface conditions fluctuated between (1) acidic and oxidizing (pH < 3, and Eh > 0.4 V); (2) acidic and slight reducing (pH < 3, and Eh < 0.4 V), and subacidic (pH > 4) to produce an alternation between the ferric oxysulfates/oxyhydroxides, sulfur and sulfates depleted in Fe, and silica-rich deposits [13,48]. The relation between layers and microstructures records a temporal sequence of processes that could start with the silica-rich layers with a pH decrease inside the orebody aquifer leading to the dissolution of the primary silica of the Peña de Hierro host rock. This could be followed with the precipitation of native sulfur and/or sulfate-rich deposits through a low pH, but under Eh conditions low enough to prevent the oxidation of Fe(II) to Fe(III). The precipitation of the microlaminar layer composed of iron oxysulfates and oxyhydroxides (Figure 2 and Figure 3, and Supplementary Figure S1) came after the Eh rise above an average of 0.4 V, where Fe(II) suffered a net oxidation to Fe(III). In this temporal succession, the brecciated microstructure was likely the last deposit to form as its skeleton is formed of subangular clasts with silica and sulfate composition, and its matrix is composed by ferric oxyhydroxides. The accumulation of the three different layers could be the response to a drop of the water level in the fractured aquifer or the inflow of more oxygenated solutions by some climatic or tectonic event. In both cases, the result would have been the formation of a terminal brecciated layer that covered the older alteration layers. Interestingly, the presence of phosphatic-rich crenulated microstructures (Figure 2, Figure 3 and Figure 4a,b) suggests that there were some additional episodes driven by microbial activity that postdated the formation of ferric oxysulfates and oxyhydroxides.

### 5.2. (Bio)mineralization and Microbial Metabolism

Following the discussion of mineralization, we can recognize from the ToF-SIMS image and spectral data three different mineralization processes that could have led by microbial activity. The formation of ferric oxysulfates and iron-depleted sulfur-rich layers have been widely reported to act as by-products from the biooxidation of the sulfide orebodies that are hosted in the Peña de Hierro basement [1,13,21]. As the oxygen supply is a limiting factor for the production of ferric iron through metabolic activity, Fe(III) will be the only accessible source for the mineralization in the vadose or shallow areas of the aquifer that have access to meteoric solutions [13]. Therefore, the distribution of iron-enriched versus iron-depleted sulfur-bearing minerals provides information regarding the environmental conditions where the mineralization is ongoing. In this geochemical context, the precipitation of the secondary sulfur-bearing minerals was a passive formation process governed by abiotic process. They have been formed from the precipitation of underground solutions that were oversaturated in different ions following the same pathways observed in the surface acidic system of Rio Tinto [49]. Although mineralization from ferric oxysulfates and oxyhydroxides was not driven by microbial processes, it has been a major agent for the preservation of biological compounds [14,15].

The association between inorganic and organic ions with distinctive microstructures suggests that they formed through specific biomineralizing processes (Supplementary Table S1). This is the case of the NO_n_^−^_(2≤n≤3)_-bearing circular and the PO_n_^−^_(2≤n≤4)_-rich crenulated microstructures (Figure 2 and Figure 4a–c) based on its relationship with the deposit layers, formed during transient episodes. They alternated with the main mineralization events that were involved in the production of most of the underground deposits. In this regard, the nitrogen-bearing microstructures have been found associated with NH_4_^+^, K^+^, Na^+^, Cl^−^, PO_4_^−^, C_4_H_3_O_5_^−^, C_12_H_25_SO_4_^−^, C_14_H_29_SO_4_^−^, and some FA adducts like palmitate (Figure 4, Figure 5, Figure 6, Figure 7, Figure 10 and Figure 11; Supplementary Table S1). The N-bearing circular microstructures apparently occur as microbores affecting the laminated, glassy, and brecciated microstructures (Figure 2 and Figure 4c). This structural arrangement is also found in the filamentous microstructures observed through the SEM-EDAX analysis (Supplementary Figure S1), which entrench on the mineral surface with a subparallel or oblique direction. The EDAX spectral analysis also shows that the filamentous elements are enriched in nitrogen as the N-bearing circular micronodules characterized through ToF-SIMS (Figure 4c; Supplementary Figure S1). If the filaments observed through SEM and the ToF-SIMS circular micronodules are the same microstructures, it comes out that they could have been formed by nitrogen oxidizers using the ferric iron or sulfate from the mineral matrix as electron acceptors [15,50,51]. Assuming that iron was the electron acceptor, the main two metabolic processes could have been the following [50]:

NH_4_^+^ + 6·Fe(OH)_3_ + 10·H^+^ → NO_2_^−^ + 6·Fe^2+^ + 16·H_2_O (6 e^−^) (6)

NO_2_^−^ + 3·Fe(OH)_3_ + 7·H^+^ → NO_3_^−^ + 3·Fe^2+^ + 8·H_2_O (3 e^−^) (7)

where the main equation including the full oxidation pathway will be:

NH_4_^+^ + 9·Fe(OH)_3_ + 17·H^+^ → NO_3_^−^ + 9·Fe^2+^ + 24·H_2_O (9 e^−^) (8)

Furthermore, the occurrence of the N-bearing microstructures (Figure 4c) embedded in the S-bearing layer that are depleted in Fe suggests that different substrates composed of like S^0^, S_2_O_3_^2−^ and SO_4_^2−^ acted as electron acceptor for the nitrogen molecular species. Such a process has been described as the following reaction [52]:

4·NH_4_^+^ + 3·SO_4_^2−^ → 4·NO_2_^−^ + 3·S^2−^ + 4·H_2_O + 8·H^+^ (9)

Although the nitrate composition of the mineralization is uncertain, the co-occurrence of the nitrate compounds with K and Na suggests that its component should be like niter (KNO_3_) and nitranite (NaNO_3_) [53].

**Figure 12:**
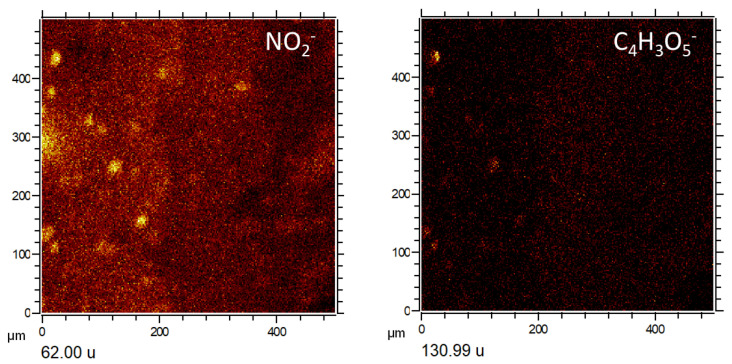
ToF-SIMS images from TA2 showing the occurrence of *m*/*z* 62.00 (NO_3_^−^) and 131.00 (C_4_H_3_O_5_^−^) that follow the same distribution.

The release of the electron acceptors from the mineral surface is done by using siderophores and organic acids [54,55]. However, the molecular preservation of siderophores is uncertain. The detection of *m*/*z* 131.00, which can tentatively be assigned to C_4_H_3_O_5_^−^, could be a trace of oxalic-like acids released by bacteria. Although this anion follows the same distribution as NO_n_^−^_(2≤n≤3)_ in TA2 (Figure 12), it does not meet the occurrence of the N-bearing ions in the other TA1 and TA3 areas. Furthermore, *m*/*z* 131.00 could also correspond to C_3_HNO_5_^−^, which might be an adduct combining organic and N-bearing inorganic compounds unrelated with the presence of oxalic compounds. The ToF-SIMS result does not prove the presence of microbial acids that mobilize ions from the mineral surface. However, the cores extracted during the drilling operations were performed by the IPBSL project [12] were analyzed for different anions like oxalate. As a result, it has illustrated the existence of oxalate with average rock content of 0.35 ppm along the 610 m depth in the borehole BH10. Therefore, it is likely that such organic acid has played a crucial role in the mobilization of different essential elements like C, N, S, and Fe by the Iberian Pyrite Belt underground microbial communities.

The combination of ToF-SIMS image and SEMS-EDAX data support that the NO_2_^−^/NO_3_^−^ circular micronodules (Figure 4c; Supplementary Figure S1) are found in two different mineral settings corresponding with the microlaminated and the glassy layers (Figure 2). As discussed above, SEM-EDAX analysis has detected the presence of N-rich long filaments settled and entrenched the microlaminar layer (Supplementary Figure S1). The N-bearing micronodules characterized by ToF-SIMS (Figure 4c) scattered inside the microlaminated layer, which suggests transversal or oblique sections of the same filaments observed under SEM (Supplementary Figure S1). If they are different traces of the identical microbial groups, their morphology and orientation demonstrate they were sequentially formed after the precipitation of a Fe oxysulfate lamina. This was done following some seasonal influx of ferric iron and sulfate that were precipitated by abiotic precipitation. The lamina formation was followed by colonization of bacteria-forming filament sheaths attacking the upper part of each mineral lamina releasing ions like Fe^3+^ and SO_4_^2−^ which could be potentially used as electron acceptors. A similar sequence of mineral precipitation and microbial attack could also have occurred during the formation of the glassy layer. The ToF-SIMS and SEM image evidences the presence of different sinuous microstructures that follow a subparallel to oblique convergent trajectories (Figure 2). They usually follow a perpendicular or transverse orientation to the glassy layer that is crossing through. N-bearing circular microstructures detected by the mapping capabilities of the ToF-SIMS are more abundant than in the microlaminated layer. They arrange along the fine and tenuous laminations that build up the glassy layer. The distribution of ions like NO_2_^−^/NO_3_^−^ and Na^+^, and C_16_H_31_O_2_^−^ (C_16:0_) (Figure 3, Figure 4, Figure 5, Figure 6, Figure 7 and Figure 11) meet the trajectory traced by the sinuous alignments. The compositional and structural configuration suggests they are traces of boring nitrogen oxidizers, which grew and drilled the interior of the mineral laminae from the surface after it was formed during a precipitation hiatus.

The microstructures enriched in different PO_n_^−^_(2≤n≤4)_-bearing anions could have been formed by the metabolic process of microbial life that has been already found in the Rio Tinto subsurface. It has been reported that bacterial strains of *Tessaracoccus* *lapidicaptus* (Gram-negative Actinobacteria) and *Acidiphilium* (Class Alphaproteobacteria) isolated from the Rio Tinto subsurface are phosphate biomineralizers [9,56]. The ToF-SIMS analysis has shown that the phosphatic compounds are formed in different microstructures, which suggests a different origin. PO_2_^−^ and PO_3_^−^ show a high concentration in the laminated microstructure, where they co-occur with the Fe- and S-bearing ions (Figure 2, Figure 3 and Figure 4a,c). However, the P-bearing anions have a maximal intensity in the patches with crenulated microstructure and tiny (<10-micron size) circular microelements (Figure 2, Figure 3 and Figure 4a,b), where it also co-occur with different adducts of phosphatic salts and lipid (Figure 5, Figure 6 and Figure 7; Supplementary Figure S8a,b). Other P-bearing anions like PO_4_^−^, and CH_4_PO_3_^−^ with a more complex distribution display a lower intensity in the crenulated and circular P-bearing microstructures.

The phosphate biomineralization could have resulted from the chemoheterotrophic metabolism that can eventually use Fe^3+^ or SO_4_^−^ as an electron acceptor [12], as it has been reported from Rio Tinto bacteria [56,57]. Chemoheterotrophic bacteria *T. lapidicaptus* and *Acidiphilium* sp. initiated the nucleation of phosphate and carbonate spheroidal nanocrystals in the external part of the bacteria wall and inside the extracellular polymeric substances (EPS) that grow to form larger carbonate structures (Supplementary Figure S12). The high availability of K, Na, Fe, and Ca likely favored a fast mineralization to form phosphatic compounds that co-precipitated with Fe-bearing carbonates like siderite (FeCO_3_) and ankerite (CaFe(CO_3_)_2_) [56,57].

In TA1, the disposition of the phosphate-rich crenulated microstructure in the whole set of laminated deposits suggests that it was formed during a transient episode after the microlamina accretion and before the formation of the brecciated layer. However, although the TA2 PO_3_-bearing discontinuous lamina lies between the cryptocrystalline-fibrous and laminated layers, its configuration is unclear. In this case, the phosphatic microstructure was likely formed after the acidic weathering of the silica-rich host rock and before the formation of the microlaminar layer, which ended the process of crack filling (Figure 2). Thus, we can only infer that the formation of the phosphatic biomineralized microstructures occurred during transitory episodes that alternated with the building and mineralization of thicker layers. It would likely represent an episode where bacteria had enough time to colonize empty mineral surfaces. In the case of TA1 phosphatic biomineralization, the internal fabric of finely crenulated laminations (Figure 2) suggests that it should have grown during several short time. The phosphate biomineralization mediated by metabolic activity would have happened with available ions in the subsurface solution, but whose concentration was not high enough to precipitate sulfates, ferric oxysulfates, or silica. The nucleation and precipitation of phosphatic compounds have been reported from chemoorganotrophic and aerobic bacteria [58] that metabolizes nitrogenated biomolecules available in the subsurface (e.g., peptides) with the production of HCO_3_^−^, NH_4_^+^ and PO_4_^3−^. As discussed above, those anions combined with available cations, in our case with K, Na and Ca precipitate different minerals in the cellular wall and inside the EPS mesh. Interestingly, the same metabolic process that releases PO_4_^3−^ by microbial degradation, is also responsible for the NH_4_^+^ production through ammonification, which is one of the N sources for the Rio Tinto underground microbial communities [15].

### 5.3. Microstructure and Molecular Associations (MMAs)

As it has been widely discussed, there is a strong correlation between the distribution of biomolecules and organic compounds with the occurrence of several microstructures found in the three target areas TA1 to TA3 (Figure 2, Figure 3, Figure 4, Figure 5, Figure 6, Figure 7, Figure 8, Figure 9, Figure 10 and Figure 11). The association between molecular compounds and microstructures (MMAs) is a conceptual procedure to integrate metabolic and biomolecular information obtained through the ToF-SIMS analysis. Such a methodological procedure seeks to reveal the microbial origin of the preserved subsurface microstructures of the Peña de Hierro basement. By doing this, we can find the following MMAs (Supplementary Table S1):

*MMA1.* It corresponds with PO_n(2≤n≤4)_^−^-bearing patchy aggregates and micron-sized nodular microstructures matching Gφ (Figure 5b and Figure 7), whose mineral composition consists of K-, Ca-, and Na-phosphatic salts. They record different lipidic compounds that can tentatively be characterized as different phospholipids (Figure 5b and Figure 7; Supplementary Figure S8; Supplementary Table S6). Furthermore, fragments of heterocyclic structures (e.g., polyketides) also match the distribution of the phosphatic patches in TA1 (Supplementary Figure S9b). As discussed above, it might be related with the activity of phosphate mineralizers like *Acidiphilium* and *Tessaracoccus* [9,56,58].

*MMA2.* Represented by one 50 micron-size ovoidal micronodule (Gω) containing PO_2_^−^ and PO_3_^−^ anions, FAs, acylglycerides, and alkanol chains (Figure 4c, Figure 6, Figure 7 and Figure 11; Supplementary Tables S5 and S6). They come also together with some S-bearing adducts like C_12_H_25_SO_4_^−^, and C_14_H_29_SO_4_^−^. Although the FA composition is dominated by C_16:0_ > C_14:0_, it shows an internal variation with FAs like C_14:0_ > C_15:0_ (Figure 11). Moreover, it is also associated with cations that could come from the fragmentation of a phosphocoline derivative and aminobacteriohopanols (Supplementary Figures S7 and S9b).

*MMA3.* Various nodules (<20 micro-sized) microstructures contain NO_n_^−^_(2≤n≤3)_ ions with a secondary occurrence of PO_4_^−^, CH_4_PO_3_^−^, and some FAs (C_16:0_ > C_14:0_). They currently occur in the microlaminated layer following the Gα pattern (Figure 4c, Figure 5b and Figure 7). Its formation has been associated with the activity of Anammox bacteria [50,51].

*MMA4.* NH_4_^+^-bearing nodular TA2 microstructures (Figure 4c, Figure 6 and Figure 9b) show a high mineralization with Fe-oxysulfates and oxyhydroxides. It exhibits a high correlation with the major ion CNS^−^ (Figure 9b). Other fragments like C_2_H_8_N^+^, C_3_H_8_N^+^, and C_4_H_10_N^+^ likely sourced from peptidic chains and few N-bearing organic adducts like C_16_H_36_N^+^ (Figure 6).

*MMA5.* Sulfate-rich iron-depleted layer and nodules with CN^−^, CON^−^, N-bearing adducts, and fragments attributed to amino acids and sphingolipids (Figure 4a,b and Figure 9, Figure 10 and Figure 11). MMA5 follows pattern Gα in both TA1 and TA2. However, in TA2, it is internally composed of two different laminas. One internal with NH_4_^+^-adducts like C_34_H_72_N^+^, C_36_H_76_N^+^, and C_38_H_80_N^+^, and the other external limiting with the microlaminated layer, which is tentatively composed of C_21_H_39_O^+^, C_21_H_41_O^+^, and C_25_H_46_N^+^ (Supplementary Figure S3).

### 5.4. Ancient Microbial Composition Recorded in the Rio Tinto Basement

The integration of the microstructure, mineral, metabolic, and molecular information resulted in different MMAs associations, which suggest the preservation of different underground microbial groups. As it has been stated, MMA1 will correspond with traces of chemoheterotrophic bacteria that have already found operating in the modern microbial community of the Rio Tinto basement [9,56,57]. They can oxidize organic matter and nucleate carbonatic and phosphatic minerals using Fe^3+^ and SO_4_^2−^ as electron acceptors. These microbes would have also been capable of synthesizing phosphatidylglycerols devoid of phosphocholine (Supplementary Figure S8; Supplementary Table S6) like *Tessaracoccus* [11]. The distribution of phosphatic patches in the subsurface (Figure 2 and Figure 4a,b) indicates the chemoheterotrophic activity started when the underground solutions had a net supply of ferric iron and sulfate. Therefore, it resulted from an environmental change to a higher redox. This would have occurred before the underground solutions increased enough and the ion concentration formed the ferric oxysulfate microlaminations. Interestingly, the presence of PO^3^-bearing nodular microstructures inside the ferric microlaminations (Figure 4a,b, Figure 5b and Figure 7) suggests that such chemoheterotrophic microbes would have operated during shorter timespans between the precipitation of two consecutive laminas.

MMA2 would represent a second microbial group with unknown metabolism. Given the molecular data, it shows an abundance in acylglycerides (e.g., DAGs) and FAs (Figure 6 and Figure 7), which could correspond to microbes with phosphorus-free membrane lipids. In this regard, Gram-positive bacteria and α-Proteobacteria synthesize membranes with a DAG backbone under conditions of phosphate limitation [59]. As MMA2 is also defined by the presence of PO_3_^−^, some fragments can be tentatively associated with a phosphocoline derivative (Supplementary Figure S7), which could come from traces of bacterial glycerophospholipidic membranes [60]. In this case, the absence of positive peaks of major adducts of phospholipids would denote a higher degradation of endolithic microorganisms. Consequently, a greater degradation of biological compounds should be expected as the endolithic structures are isolated from the main mineralizing underground solution.

The record of a third group of microbes would be represented by MMA3. It was formed by nitrogen oxidizers that used NH_4_^+^ and NO_2_^−^ as electron donors. As discussed by Fernández-Remolar et al. [15], the origin of nitrogen would have been two different sources that include NH_4_^+^, and NO_2_^−^ from protein ammonification and the host rock where it was sequestered through hydrothermalism [61]. The presence of PO_4_^−^, CH_4_PO_3_^−^, and FAs could be revealed as traces of highly degraded phospholipids of endolithic microorganisms (Figure 4c; Supplementary Figure S1) as shown in MMA2.

In TA2, the presence of NH_4_^+^-bearing nodular microstructures with CNS^−^, traces of peptides mineralized by ferric oxysulfates characterize a very distinctive MMA4 (Figure 4b, Figure 6, Figure 7 and Figure 9b; Supplementary Figure S1). The co-occurrence of both protein traces and ammonia evidence the nodular microstructures result from the mineralization of microbial ammonification of proteinic compounds. Furthermore, the ammonia production is also causally linked with the N cycle as this compound co-occurs with NO_2_^−^ (Figure 4c). There is no molecular information that can provide any insight into the microbes involved in ammonia production. Therefore, few can be known about the microbial origin of the NH4^+^-bearing nodules except that they were likely formed by decomposers that broke up the proteins to produce ammonia and amino acids [62].

The molecular composition of MMA5 is mainly represented by N-bearing organics, whose ions are likely related to sphingolipids [36]. However, different N-bearing positive and negative ions characterized as amino acid fragments in MMA4, are also found in MMA5. Consequently, such an association suggests either the co-occurrence of traces of peptidic chains with sphingolipids, or the production of some of the same ions by the sphingolipid fragmentation as they also have amino and carboxylic groups [60]. Although the molecular record provides little information about the microbial source, the presence of tentative sphingolipids can ascertain some clue about the origin for such association. In this regard, sphingolipid synthesis has been described in few bacteria groups (e.g., CFB Group, and δ-Proteobacteria), which build outer membranes devoid of lipopolysaccharides [59]. In turn, the occurrence of amino acid fragments suggests that the molecular composition could also resulted from the mineralization of EPS, which have been found in the subsurface [6,12].

The MMA5 is built from a microstructure that shows an internal layering composed of N-bearing large fragments (e.g., C_19_H_42_N^+^, C_34_H_72_N^+^, C_36_H_76_N^+^, and C_38_H_80_N^+^) (Supplementary Figure S3). They have been found associated with sphingolipid fragments in eukaryotic filamentous structures of the Upper Gossan deposits. MMA5 is limiting with the microlaminated layer through an 80-micron thick lamina outlined by cations characterized as C_21_H_39_O^+^, C_21_H_41_O^+^, and C_25_H_46_N^+^. Although this lamina has an uncertain origin, its presence suggests a record of a replacement in the microbial community. Such a microbial change following the environmental alternation is also recorded in the mineral composition (Figure 2, Figure 3b and Figure 4b). Interestingly, the occurrence of a second layer with C_19_H_42_N^+^, C_34_H_72_N^+^, C_36_H_76_N^+^, and C_38_H_80_N^+^ (Supplementary Figure S3) indicates the alternance of microbial populations in a subsurface environment with changing conditions.

### 5.5. Preservation of Biomolecules and Organic Compounds

The high ion concentration of the subsurface solutions and the microbial biomineralization have played an essential role in the preservation of ancient microbial communities that populated the underground habitats since the Cenozoic [14]. As it has been discussed, the leading preservation process has been the production of solutions that are highly concentrated in ferric and sulfate ions through the biooxidation of the sulfide orebodies [4,13]. Under these circumstances, the main preservation pathway has been the mineral coating and permineralization of biological structures through the formation of Fe^3+^ and SO_4_^2−^ inorganic polymers [49,63]. In the surface waters, it has been shown that the seasonal alternation between the rainy solutions in spring/fall and overconcentration through evaporation in summer produces the raw material to mineralize the different biological remains. In the subsurface, the sulfate and oxysulfate layers evidence the same process of seasonal mineralization (Figure 2; Supplementary Figure S1). The ToF-SIMS analysis does not provide evidence if cellular microstructures have been preserved through a mineralization. However, Fernández-Remolar and Knoll [49] have reported the preservation of filamentous and cocci-like microstructures by using light microscopy and SEM-EDAX (Supplementary Figure S1). Although the data resolution is not high enough to reveal the relation between mineralization and the original microbial structure, the observation of such information in larger biological structures supports that the organic matrix is internally replaced by crypto to microcrystalline iron oxysulfates/oxyhydroxides [49]. Consequently, it is also expected that the cell wall and cytoplasmatic interior of bacteria could be mineralized following a similar process. In this regard, Preston et al. [64] have reported the mineralization of the cellular wall of Rio Tinto filamentous bacteria serving as nucleation surface for iron oxide and sulfate mineralization.

The wall mineralization follows the same process observed in the biomineralization through phosphatic and carbonatic minerals that formed some of the microstructures we have discussed [9,58]. The cell mineralization has been explained through the bond of positively charged inorganic ions with the negative organic wall surface. This process happens under neutral to subalkaline conditions through bonding ferric ions with different functional organic groups forming the bacteria cell [65]. However, the current hyperacidic conditions should reverse the charge balance in the cellular wall. The ToF-SIMS analysis has shown the presence of different alkylsulfates (AKS) such as C_12_H_25_SO_4_^−^, C_13_H_29_SO_4_^−^, C_14_H_29_SO_4_^−^, C_14_H_29_SO_5_^−^, and C_16_H_33_SO_4_^−^ (Supplementary Figures S5b and S7) [14,15]. They are mainly sourced in the microlaminated layers composed of ferric oxysulfates and oxyhydroxides. While AKS could be produced by the simple combination of different SO_n_^−^_(3≤n≤4)_ ions with fragmented organic compounds, its formation could be also related with the adsorption of the sulfate groups with a protonated organic surface [15]. In this scenario, far from neutral or subalkaline, sulfate would become the inorganic bonding group to the cellular surface instead of ferric iron [65].

Furthermore, TA2 include two microlaminas containing N-bearing adducts like C_19_H_42_N^+^, C_34_H_72_N^+^, C_36_H_76_N^+^, and C_38_H_80_N^+^ inside the glassy and microlaminated sulfate-bearing layers (Figure 2 and Figure 3a,b; Supplementary Figure S3). Interestingly, Fernández-Remolar et al. [14] describe a close association between sulfate adducts, and fragments of N-bearing lipids with filamentous framework microstructures. The morphology and composition suggest that it can be the relict of 25 Ma biofilms that were mineralized by ferric oxysulfates. Indeed, C_19_H_42_N^+^, C_34_H_72_N^+^, C_36_H_76_N^+^, and C_38_H_80_N^+^ are found among the different N-bearing compounds that show the same distribution as the filamentous mesh microstructures. The presence of N-bearing compounds in the TA2 glassy layer (Supplementary Figure S3) points out that they could correspond with protein or sphingolipid fragments of >6 Ma EPS. Such microbial compounds found in active underground Rio Tinto biofilms [8] may have experienced a fast mineralization by sulfate as reported in the oldest Upper Gossan materials of Rio Tinto [14].

## 6. Conclusions

The molecular and image ToF-SIMS analyses of the underground deposits that formed > 6 Ma in the Rio Tinto subsurface (Figure 1) have provided essential information of the ancient microbial communities involved in the gossan formation. The combination of the inorganic and organic composition with the microstructure has revealed the main mineralization processes, metabolic activity and microbial communities that were involved in the preservation of organic compounds in the deepest areas of the Peña de Hierro gossan. By combining all the image, molecular, and elemental information, it is possible to recognize five layers (microlaminated, glassy, fibrous-cryptocrystalline, brecciated, and crenulated) that are composed by four different mineral groups like Fe-depleted sulfates, ferric oxysulfates and oxyhydroxides, silica-rich, and phosphatic salts (Figure 2, Figure 3 and Figure 4; Supplementary Table S1). Further, the ToF-SIMS analysis of the ion distribution of inorganic and organic compounds has resulted in the identification of 11 morphological groups Gα to Gο (Figure 5, Figure 6 and Figure 7; Supplementary Table S2).

The ToF-SIMS spectral analysis has identified the presence of diverse organic compounds that tentatively include hydrocarbons, PAHs, acylglycerides, sphingolipids, phospholipids, peptide fragments, hopanoids, traces of sterols, and various unknown compounds which can correspond with heterocyclic compounds like polyketides. The preserved FA record (Figure 8) supports that it was originated from bacterial communities. The molecular characterization was followed by a principal component analysis (PCA) of some fragments like major anions, FA, and peptidic/amino acid fragments. This was carried out for characterizing the molecular variation between and inside the different microstructures. The combination of the molecular composition with the various layers of information described above has led to the identification of five main microstructure and molecular associations (MMAs). Those MMA1 to MMA5 encode the microbial information that has been preserved in form of the co-associated molecular compounds and distinctive microstructures (Supplementary Table S1). Furthermore, the distribution of the different MMAs also suggests they were formed by the succession of different microbial populations in a changing paleoenvironmental condition varying from subacidic and anoxic to hyperacidic and oxidizing.

The presence of various microstructures recording different metabolic pathways in merely 500 micron-size squared area pictures a highly active and ample underground microbial community. Its heterogeneous distribution in the Rio Tinto basement evidence that the subsurface was temporally and spatially fragmented by environmental conditions in disparate microniches. However, such an ecological framework favored the presence of a diverse microbiota sustained in the biooxidation of sulfide orebodies as a primary source of energy. The biooxidation energy distributed along the subsurface microbial communities has built an intricated network of mater and energy transfer. It has been recorded as diverse microstructures showing that the microbial communities operating various cycles like C, N, P, N, S and Fe for millions of years. Furthermore, the high organic content in the analyzed sample estimated by the high concentration of organic compounds declares the biomass is abundant.

While the sulfide biooxidation is involved in maintaining stablility in the chemical conditions of the surface and subsurface of Rio Tinto habitats through the ferric iron buffer [63], the oxidation has also played an essential role in the mineralization and preservation of biological traces. Thereby, it has become the primary agent to record the biotic and molecular structures produced by the microbial communities that are inhabiting the acidic habitat since 25 Ma [14,15,49]. The Rio Tinto hyperacidic system shows that extreme conditions can also be associated with diverse microbial communities with a high preservation potential. However, the most intriguing fact is that the homeostatic control of the environment and the high preservation of biological traces are ultimately controlled by few microbial taxa that are fed on the oxidation of the sulfide orebodies [5,66].

## Data Availability

The data presented in this study are available on request from the corresponding author.

## Supplementary Materials

The following are available online at https://www.mdpi.com/article/10.3390/microorganisms9081592/s1, Figure S1. Composition of two SEM-EDAX images of sample BH8-2c. It shows ferruginous materials infilling a crack formed in the Peña de Hierro basement; Figure S2. Red, Green, and Blue (RGB) image merging HSO_4_^−^, FeO_2_H^−^, and SiO_2_^−^ + Si_2_H_4_O^−^ in TA3. It displays the distribution of the three main unit layers including the fibrous-cryptocrystalline (fb) enriched in SiO_2_, the microbrecciated (mb) containing microclasts with diverse composition, and the microlaminated (ma) with Fe-bearing oxysulfate/oxyhydroxide minerals ((SO_4_)FeOx); Figure S3. ToF-SIMS RGB image combination in TA2. It reveals the internal fabric in glassy (ms) and microlaminated (ma) layers consisting of different laminas; Figure S4. Occurrence of hydrocarbon fragments in sample BH8-24c; Figure S5. ToF-SIMS ion images and RGB (Red/Green/Blue) image; Figure S6. Mass spectrum (intensity units in cps) in the range of 450 to 600 Da. It shows different peaks of NH_4_^+^-adducts and dyacylglycerides found in TA2; Figure S7. ToF-SIMS cation images tentatively identified as a phosphocholine derivative fragments. They occur at a higher concentration in the Gω microstructure found in TA2; Figure S8. Mass spectra (intensity units in cps) showing the distribution of P-bearing compounds; Figure S9. ToF-SIMS images of adducts tentatively identified as (a) aminobacteriohopanol and (b) heterocyclic compounds; Figure S10. Principal component analysis (PCA) performed for the TA1 cation data identified as amino acid fragments; Figure S11. Principal component analysis (PCA) performed for the TA2 cation data identified as amino acid fragments; Figure S12. SEM-EDAX image showing different carbonatic microstructures. They likely resulted from the microbial biomineralization in the sample BH8-24c from the Peña de Hierro basement; Table S1. Association between distinctive microstructures with potential biological origin and characteristic ions. They have been used to characterize the association between microstructures and molecular compounds (MMAs). Table S2. Identification of the different morphological groups. It has been done through the molecular distribution in the underground ferruginous materials of Peña de Hierro using the ion mapping through the ToF-SIMS. Table S3. Cation list of hydrocarbon fragments and NH_4_^+^ adducts found in TA1 and TA2 of sample BH8-24c. Table S4. List of [M − H]^+^ and M^+^ of polycyclic aromatic hydrocarbons (PAHs) found in BH8-24c. Table S5. List of the FA [M − H]^−^ adducts identified in the target areas TA1, TA2, and TA3 of sample BH8-24c. Table S6. Different positive and negative ions of lipids obtained in TA1 and TA2 of sample BH8-24c. Table S7. List of positive and negative ions from the peptide and/or amino acid fragmentation in TA1 and TA2 of sample BH8-24c. Table S8. List of fragments attributed to sterols and hopanoids recognized in TA1 and TA2 of sample BH8-2c.
