# Supplementary material for: Preservation of Underground Microbial Diversity in Ancient Subsurface Deposits (>6 Ma) of the Rio Tinto Basement
Source: Microorganisms. 2021 Jul 27;9(8):1592. doi: 10.3390/microorganisms9081592 (PMC8400296; doi:10.3390/microorganisms9081592)
Supplement: Supplementary file 1 [file microorganisms-09-01592-s001.zip › microorganisms-1245648-supplementary.pdf]

Preservation of underground microbial diversity in ancient subsurface deposits (> 6 Ma) of the Rio Tinto basement

David C Fernández-Remolar[1,2,*], David Gómez -Ortiz[3], Per Malmberg[4], Ting Huang[1,2], Yan Shen[1,2], Angélica Anglés[,2], Ricardo Amils[5,6]

[1]State Key Laboratory of Lunar and Planetary Sciences, Macau University of Science and Technology, Macau, PR China; dcfremolar@must.edu.mo
[2]CNSA Macau Center for Space Exploration and Science, Macau, PR China
[3]ESCET-Área de Geología, Universidad Rey Juan Carlos, 28933 Móstoles, Madrid, Spain; david.gomez@urjc.es
[4]Chemistry and Chemical Engineering, Chalmers University of Technology, Kemivägen 10, SE-412 96 Gothenburg, Sweden; malmper@chalmers.se
[5]Centro de Biología Molecular Severo Ochoa (CSIC-UAM), Universidad Autónoma de Madrid, Madrid, Spain; ramils@cbm.csic.es
[6]Centro de Astrobiología (CSIC-INTA), 28850 Torrejón de Ardoz, Spain
* Correspondence: dcfremolar@must.edu.mo

Supplementary File

Contents: Supplementary Figures 1 to 12, and Supplementary Tables 1 to 8

Units in figures and tables: cps (counts per second), m/z (mass divided by charge number, when z = 1), error (ppm), M (molecular mass)

1. Supplementary Figures

Supplementary Figure 1. Composition of two SEM-EDAX images of sample BH8-2c showing ferruginous materials infilling a crack formed in the Peña de Hierro basement. (a) displays an internal layering with a complex mineral composition that includes silica, ferric oxysulfates, and oxyhydroxides matching some of the layers identified through the ToF-SIMS SEM microimages like the microlaminated (ma) and fibrous-cryptic (fb) layers (see Figure 2b). The spectra 1 and 2 collected from a couple of microlaminated layers (ma) agrees with a ferruginous oxysulfate and/or oxyhydroxide mineralogy as they have a higher concentration in Fe, O, and S. In contrast, spectrum 3 has been obtained from weathered host rock which microstructure has been characterized as fibrous-cryptocrystalline (fb). In this last case, the occurrence of Mg and K and high intensity in Si, and a lowering in S, suggests that it is composed of phyllosilicates and silica. (b) shows the presence of a fibrous-cryptocrystalline (fb) layer, which composition obtained by the spectrum 1 reveals a silica enrichment (9 wt%) and C (8.7 wt%) but a low Fe concentration (6.3 wt%). In turn, the spectrum 2 obtained in the microlaminated layer (ma) shows a substantial decrease in Si to 3.2 wt% but an intense increment in Fe to 29.0 wt% for Fe. Such an element concentration is consistent with a mineral composition formed by ferric oxyhydroxides. Both spectra 1 and 2 have a notable presence of N (2.5 to 3.6 wt%), which is likely associated with the occurrence of the $NO^{-}_{n(2 \leq n \leq 3)}$-bearing micronodular structures found in TA1 and TA2 (see Figure 4c).

Supplementary Figure 2. Red, Green, and Blue (RGB) image merging for HSO_4^-, FeO_2H^-, and SiO_2^- + $Si_2H_4O^-$ in TA3. It displays the distribution of the three main unit layers including the fibrous-cryptocrystalline (fb) enriched in SiO_2, the microbrecciated (mb) containing microclasts with diverse composition, and the microlaminated (ma) with Fe-bearing oxysulfate/oxyhydroxide minerals ((SO_4)FeOx).

Supplementary Figure 3. ToF-SIMS RGB image combination in TA2 revealing the internal fabric in glassy (ms) and microlaminated (ma) layers consisting of different laminas. The Red image comes from the distribution of $C_5H_{11}O_4^-$ that appears along the glassy layer (ms). In turn, the Green image resulting from the sum of $C_{21}H_{39}O^+$, $C_{21}H_{41}O^+$, and $C_{25}H_{44}O^+$ traces a sinuous lamina defining a boundary between the glassy (ms) and the microlaminated (ma) layers. The Blue image resulting from the sum of $C_{13}H_{30}N^+$, $C_{18}H_{40}N^+$, $C_{20}H_{44}N^+$, $C_{21}H_{46}N^+$, and $C_{22}H_{48}N^+$ reveals a couple of internal laminas in both glassy (ms) and microlaminated (ma) layers associated with the distribution of N-bearing fragments in TA2.

Supplementary Figure 4. (a) Abundance of saturated and monounsaturated $\leq C_8$ hydrocarbon fragments in TA1 and TA2. (b) ToF-SIMS image distribution of propyl and butyl fragments in TA1 and TA2. (c) A couple of microstructures consisting of the laminas described in Supplementary Figure 3 and the NH_4^+-micronodules defining Gg sourcing two different sets of N-bearing cations in TA2. They have been tentatively corresponded with N-bearing lipids (e.g., sphingolipids), and peptides.

Supplementary Figure 5. ToF-SIMS ion images and RGB (Red/Green/Blue) image overlay for a set of $[M-H]^-$ FA adducts (see Supplementary Table 5) in the three target areas TA1, TA2, and TA3. (a) Shows that in TA1 FAs have a varying distribution in the different nodular microstructures, where $C_{14:0}$ (*m/z* 227.20) is the main FA in the sulfate-rich microclasts of the microbrecciated layer (mb). (b) TA2 FAs are predominantly found in the Gw microstructure that is associated with acylglycerides, but also in tiny micronodular structures that come together with $NO^-_{n(2\leq n\leq 3)}$ ions. (c) In TA3, the majority of FAs are principally occurring in the mirobrecciated (mb) and microlaminated (ma) layers, excepting a circular micronodule (white arrow) that show a high intensity in $C_{8:0}$ and $C_{9:0}$ (*m/z* 143.11, and 157.12).

Supplementary Figure 6. Mass spectrum (intensity units in cps) in the range of 450 to 600 Da showing different peaks of NH_4^+-adducts and dyacylglycerides found in TA2.

Supplementary Figure 7. ToF-SIMS images of cations tentatively identified as fragments of a phosphocholine derivative occurring at a higher concentration in the Gw microstructure found in TA2.

Supplementary Figure 8. Mass spectra (intensity units in cps) showing the distribution of P-bearing compounds in TA1 (a) and TA2 (b) corresponding with phosphatic salt fragments and phospholipid adducts (Supplementary Table 6).

Supplementary Figure 9. ToF-SIMS images of adducts that have been tentatively identified as aminobacteriohopanol (a) and heterocyclic compounds (b).

Supplementary Figure 10. Principal component analysis (PCA) performed for the ToF-SIMS cation data (see Supplementary Table 7) that have been identified as amino acid fragments in TA1. For

this analysis, 32 cations have been considered. The results suggests that in TA1 the main source of amino acid fragments comes from the different sulfate-rich clasts embedded in the microbrecciated layer (mb). In turn, PC2 and PC5 show that some fragments (e.g., *m/z* 42.04, and 56.06) are secondarily originated in the microlaminated and silica-rich fibrous-cryptocrystalline layers.

Supplementary Figure 11. Principal component analysis (PCA) performed for the ToF-SIMS cation data (see Supplementary Table 7) that have been identified as amino acid fragments in TA2. To perform this analysis, 30 N-bearing cations have been considered. The PCA has revealed three different areas sourcing the amino acid fragments as the glassy layer (PC1 and PC2), the microlaminated layer (PC3), and the NH_4^+-bearing micronodules (PC4, PC13, and PC15).

Supplementary Figure 12. SEM-EDAX image showing the occurrence of different carbonatic microstructures resulting from the microbial biomineralization in the sample BH8-24c collected in the Peña de Hierro basement.

2. Supplementary Tables

Supplementary Table 1. Identification of the different morphological groups through the molecular distribution in the underground ferruginous materials of Peña de Hierro using the ion mapping through the ToF-SIMS.

Supplementary Table 2. Identification of the different morphological groups through the molecular distribution in the underground ferruginous materials of Peña de Hierro using the ion mapping through the ToF-SIMS.

Supplementary Table 3. Cation list of hydrocarbon fragments and $NH4^+$ adducts found in TA1 and TA2 of the sample BH8-24c collected in the Peña de Hierro weathered basement.

Supplementary Table 4. List of $[M-H]^+$ and M^+ of different polycyclic aromatic hydrocarbons (PAHs) found in the ferruginous underground materials of Peña de Hierro.

Supplementary Table 5. List of the FA $[M – H]^-$ adducts identified in the three target areas TA1, TA2, and TA3 of the BH8-24c Peña de Hierro sample by using ToF-SIMS.

Supplementary Table 6. Different positive and negative ions of lipids obtained through the ToFSIMS spectral analysis in TA1 and TA2 of sample BH8-24c.

Supplementary Table 7. List of positive and negative ions produced from the fragmentation of preserved peptidic and/or amino acids obtained in TA1 and TA2 by the ToF-SIMS spectral analysis of sample BH8-24c obtained in the underground ferruginous materials of Peña de Hierro.

Supplementary Table 8. List of fragments attributed to different sterols and hopanoids that have been recognized in TA1 and TA2 of sample BH8-2c through the ToF-SIMS spectral analysis.

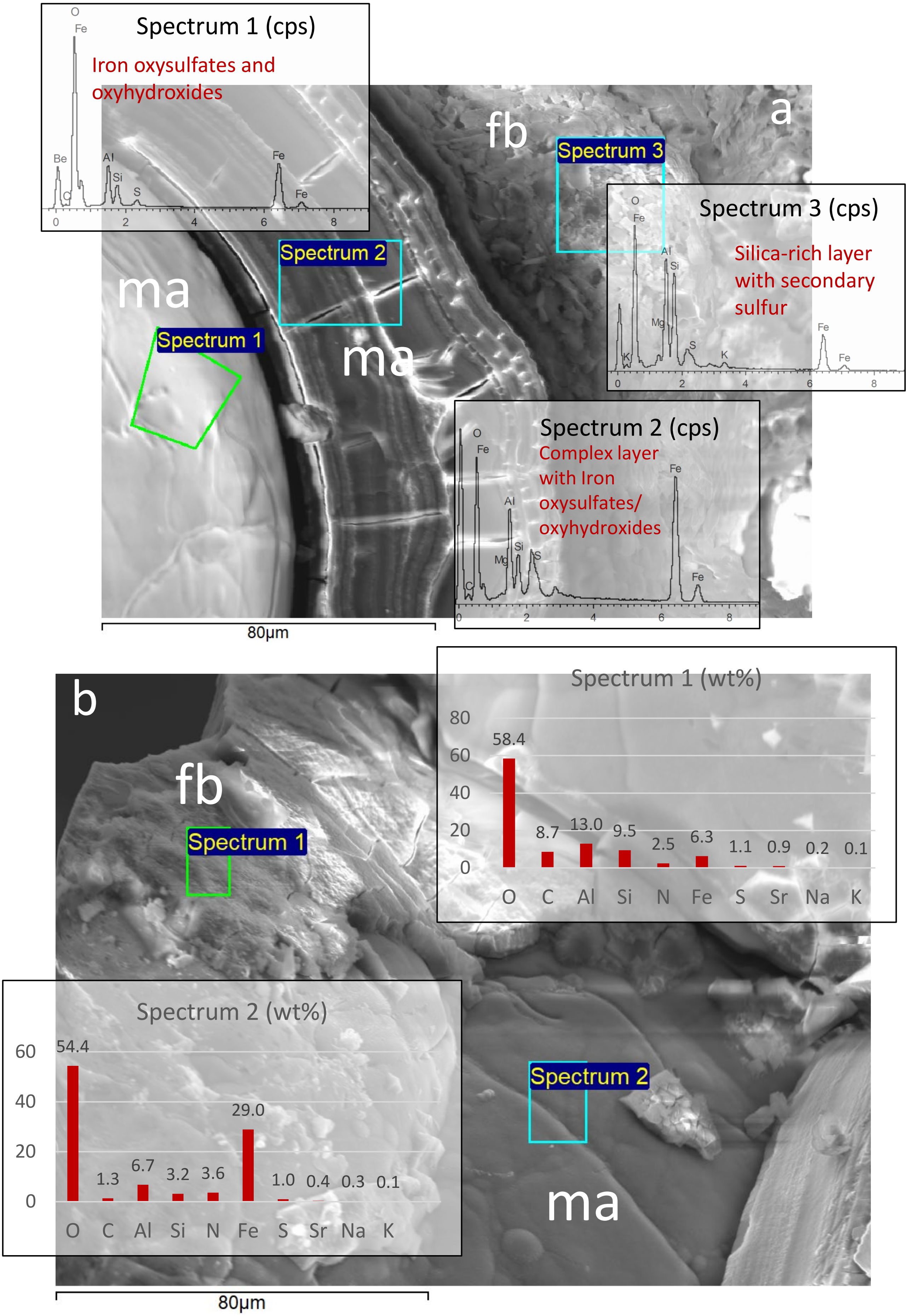
a
fb
ma
ma
Spectrum 1
Spectrum 2
Spectrum 3
Spectrum 1 (cps)
Iron oxysulfates and oxyhydroxides
Spectrum 3 (cps)
Silica-rich layer with secondary sulfur
Spectrum 2 (cps)
Complex layer with Iron oxysulfates/ oxyhydroxides
80µm
b
fb
ma
Spectrum 1
Spectrum 2
Spectrum 1 (wt%)
58.4 8.7 13.0 9.5 2.5 6.3 1.1 0.9 0.2 0.1
O C Al Si N Fe S Sr Na K
Spectrum 2 (wt%)
54.4 1.3 6.7 3.2 3.6 29.0 1.0 0.4 0.3 0.1
O C Al Si N Fe S Sr Na K
80µm

Supplementary Figure 1. Composition of two SEM-EDAX images of sample BH8-2c showing ferruginous materials infilling a crack formed in the Peña de Hierro basement. (a) displays an internal layering with a complex mineral composition that includes silica, ferric oxysulfates, and oxyhydroxides matching some of the layers identified through the ToF-SIMS SEM microimages like the microlaminated (ma) and fibrous-cryptic (fb) layers (see Figure 2b). The spectra 1 and 2 collected from a couple of microlaminated layers (ma) agrees with a ferruginous oxysulfate and/or oxyhydroxide mineralogy as they have a higher concentration in Fe, O, and S. In contrast, spectrum 3 has been obtained from weathered host rock which microstructure has been characterized as fibrous-cryptocrystalline (fb). In this last case, the occurrence of Mg and K and high intensity in Si, and a lowering in S, suggests that it is composed of phyllosilicates and silica. (b) shows the presence of a fibrous-cryptocrystalline (fb) layer, which composition obtained by the spectrum 1 reveals a silica enrichment (9 wt%) and C (8.7 wt%) but a low Fe concentration (6.3 wt%). In turn, the spectrum 2 obtained in the microlaminated layer (ma) shows a substantial decrease in Si to 3.2 wt% but an intense increment in Fe to 29.0 wt% for Fe. Such an element concentration is consistent with a mineral composition formed by ferric oxyhydroxides. Both spectra 1 and 2 have a notable presence of N (2.5 to 3.6 wt%), which is likely associated with the occurrence of the $NO^{-}_{n(2\leq n\leq 3)}$-bearing micronodular structures found in TA1 and TA2 (see Figure 4c).

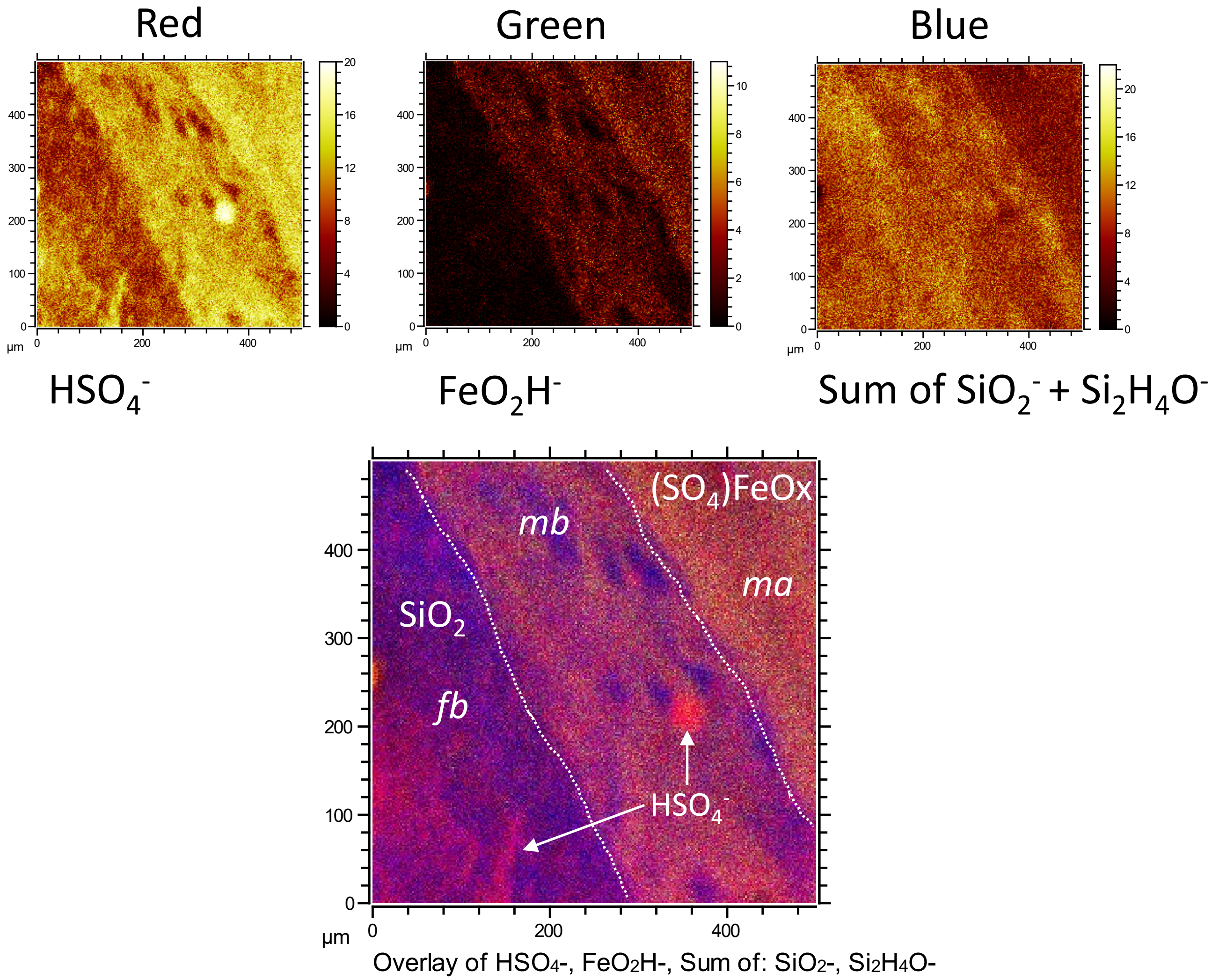

Supplementary Figure 2. Red, Green, and Blue (RGB) image merging for HSO_4^-, FeO_2H^-, and SiO_2^- + $Si_2H_4O^-$ in TA3. It displays the distribution of the three main unit layers including the fibrous-cryptocrystalline (fb) enriched in SiO_2, the microbrecciated (mb) containing microclasts with diverse composition, and the microlaminated (ma) with Fe-bearing oxysulfate/oxyhydroxide minerals ($(SO_4)FeOx$).

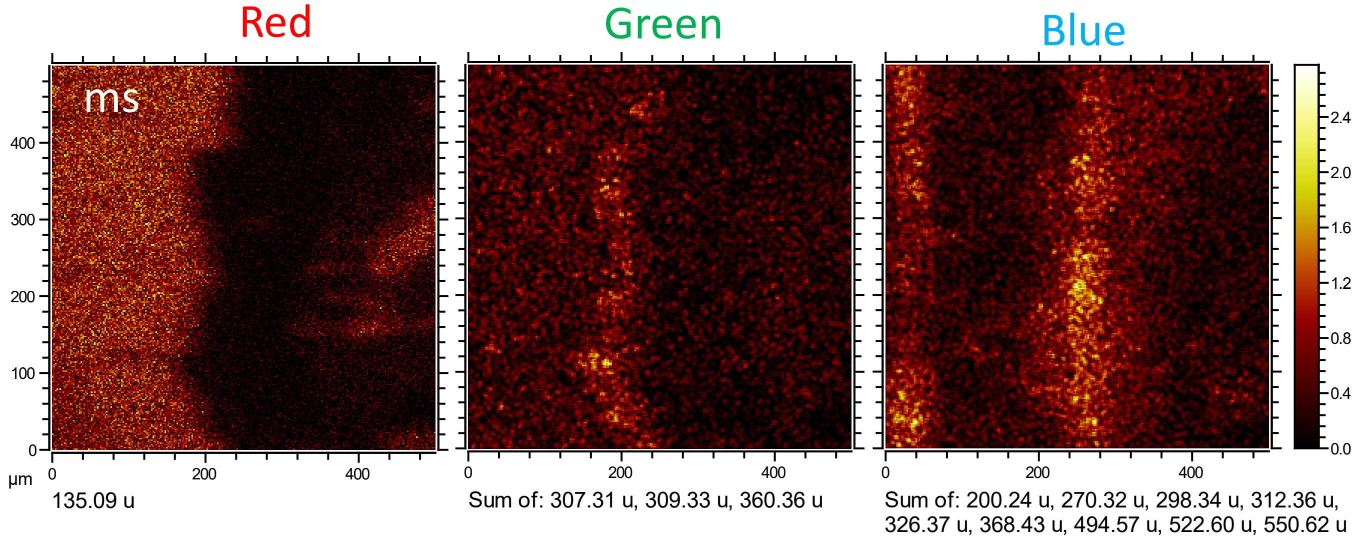

Image	m/z	Compound
Red	135.09	$C_5H_{11}O_4^-$
Green	307.31	$C_{21}H_{39}O^+$
	309.33	$C_{21}H_{41}O^+$
	360.36	$C_{25}H_{44}O^+$
Blue	200.24	$C_{13}H_{30}N^+$
	270.32	$C_{18}H_{40}N^+$
	298.34	$C_{20}H_{44}N^+$
	312.36	$C_{21}H_{46}N^+$
	326.37	$C_{22}H_{48}N^+$

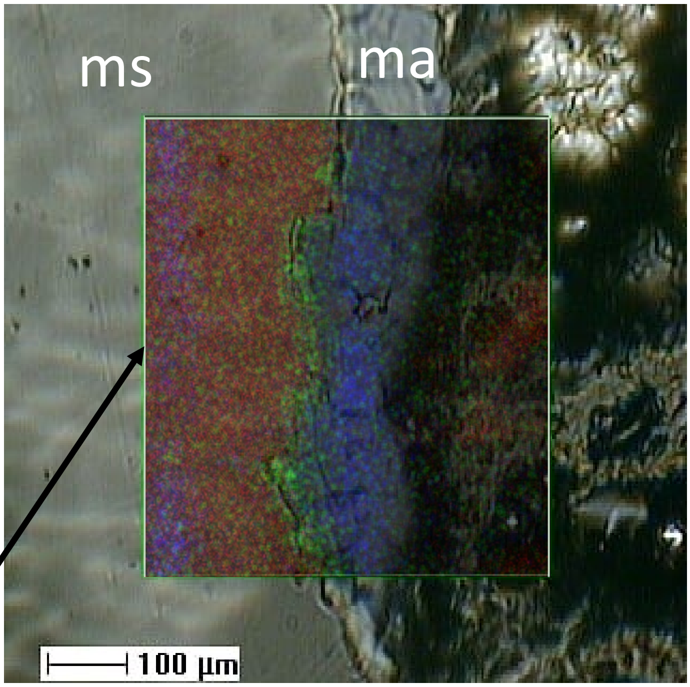

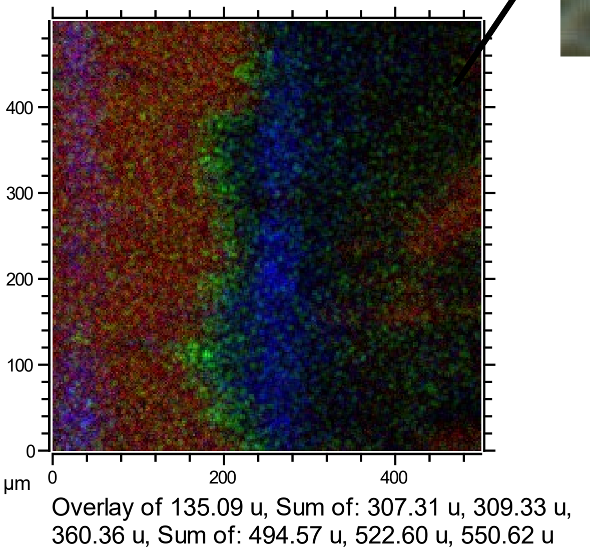

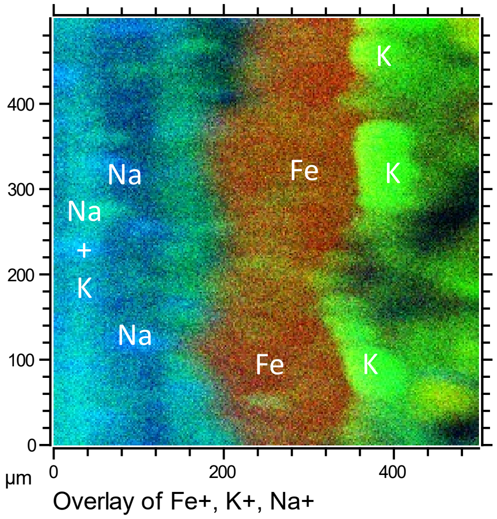

Supplementary Figure 3. ToF-SIMS RGB image combination in TA2 revealing the internal fabric in glassy (ms) and microlaminated (ma) layers consisting of different laminas. The Red image comes from the distribution of $C_5H_{11}O_4^-$ that appears along the glassy layer (ms). In turn, the Green image resulting from the sum of $C_{21}H_{39}O^+$, $C_{21}H_{41}O^+$, and $C_{25}H_{44}O^+$ traces a sinuous lamina defining a boundary between the glassy (ms) and the microlaminated (ma) layers. The Blue image resulting from the sum of $C_{13}H_{30}N^+$, $C_{18}H_{40}N^+$, $C_{20}H_{44}N^+$, $C_{21}H_{46}N^+$, and $C_{22}H_{48}N^+$ reveals a couple of internal laminas in both glassy (ms) and microlaminated (ma) layers associated with the distribution of N-bearing fragments in TA2.

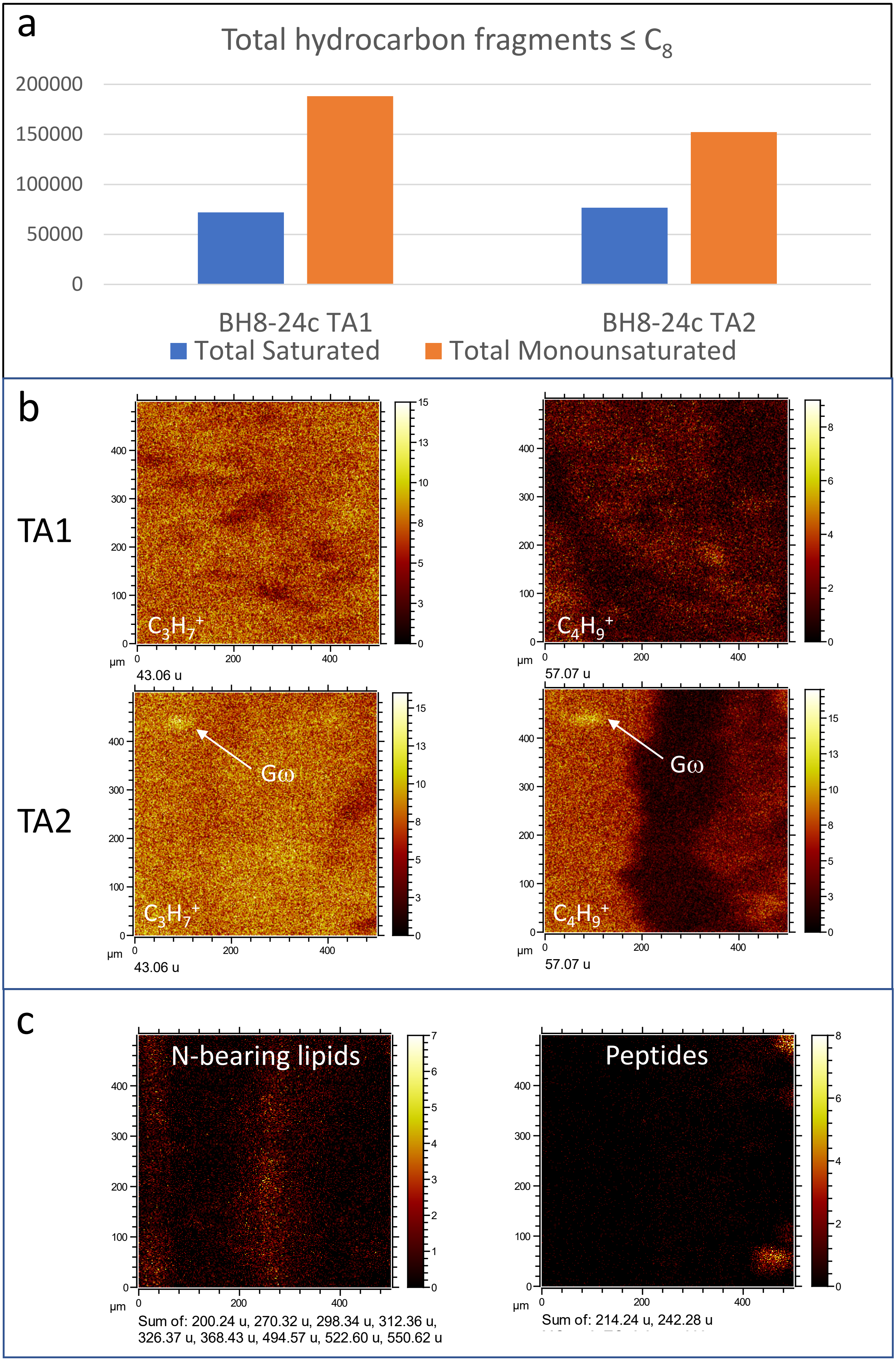

a
Total hydrocarbon fragments ≤ C8
200000
150000
100000
50000
0
BH8-24c TA1
BH8-24c TA2
Total Saturated
Total Monounsaturated
b
TA1
TA2
C3H7+
43.06 u
C4H9+
57.07 u
Gω
µm
c
N-bearing lipids
Sum of: 200.24 u, 270.32 u, 298.34 u, 312.36 u, 326.37 u, 368.43 u, 494.57 u, 522.60 u, 550.62 u
Peptides
Sum of: 214.24 u, 242.28 u

Supplementary Figure 4. (a) Abundance of saturated and monounsaturated ≤ C_8 hydrocarbon fragments in TA1 and TA2. (b) ToF-SIMS image distribution of propyl and butyl fragments in TA1 and TA2. (c) A couple of microstructures consisting of the laminas described in Supplementary Figure 3 and the NH_4^+-micronodules defining Gγ sourcing two different sets of N-bearing cations in TA2. They have been tentatively corresponded with N-bearing lipids (e.g., sphingolipids), and peptides.

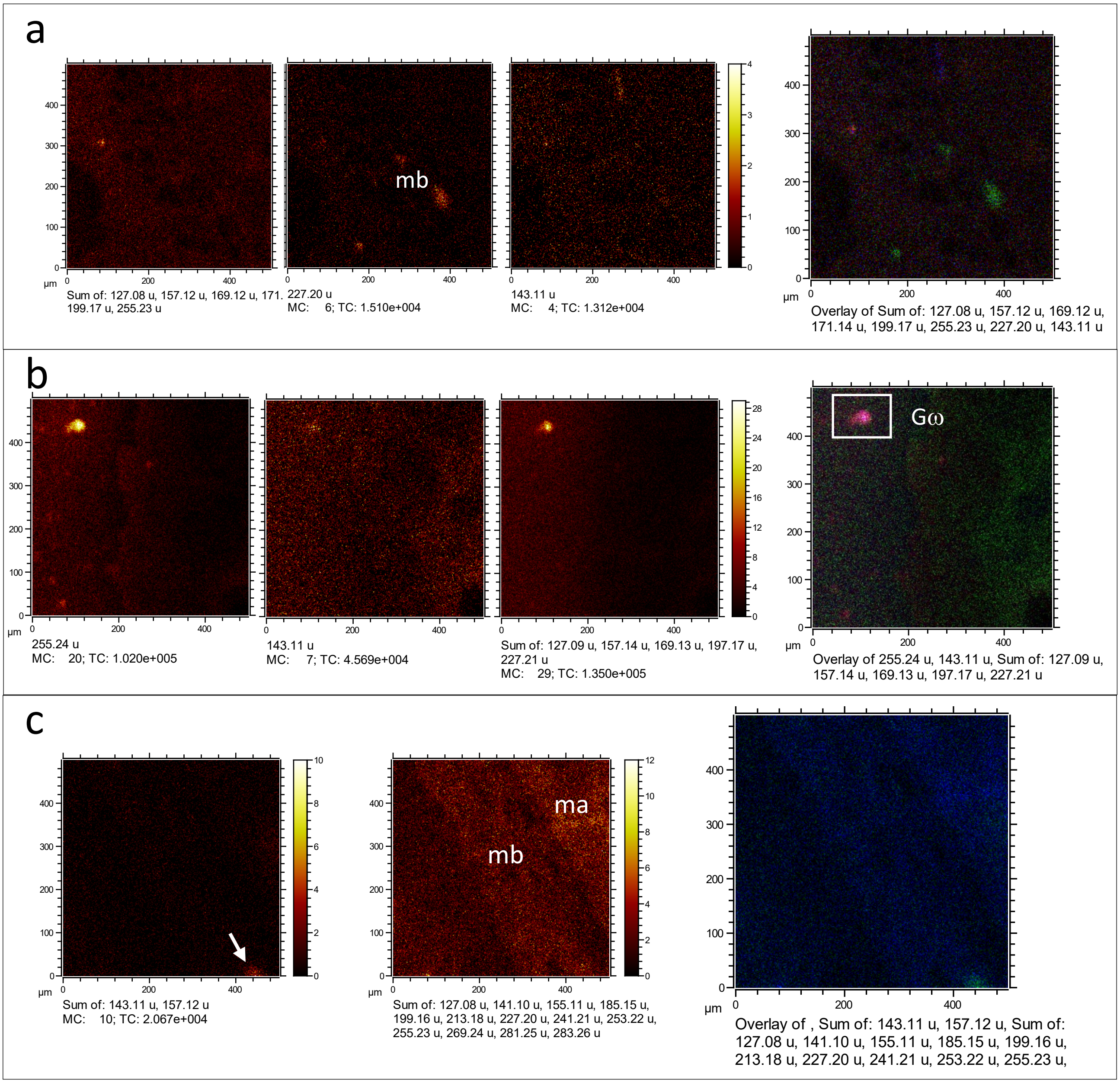

Supplementary Figure 5. ToF-SIMS ion images and RGB (Red/Green/Blue) image overlay for a set of $[M – H]^-$ FA adducts (see Supplementary Table 5) in the three target areas TA1, TA2, and TA3. (a) Shows that in TA1 FAs have a varying distribution in the different nodular microstructures, where $C_{14:0}$ (*m/z* 227.20) is the main FA in the sulfate-rich microclasts of the microbrecciated layer (mb). (b) TA2 FAs are predominantly found in the Gω microstructure that is associated with acylglycerides, but also in tiny micronodular structures that come together with $NO^-_{n(2\leq n\leq 3)}$ ions. (c) In TA3, the majority of FAs are principally occurring in the mirobrecciated (mb) and microlaminated (ma) layers, excepting a circular micronodule (white arrow) that show a high intensity in $C_{8:0}$ and $C_{9:0}$ (*m/z* 143.11, and 157.12).

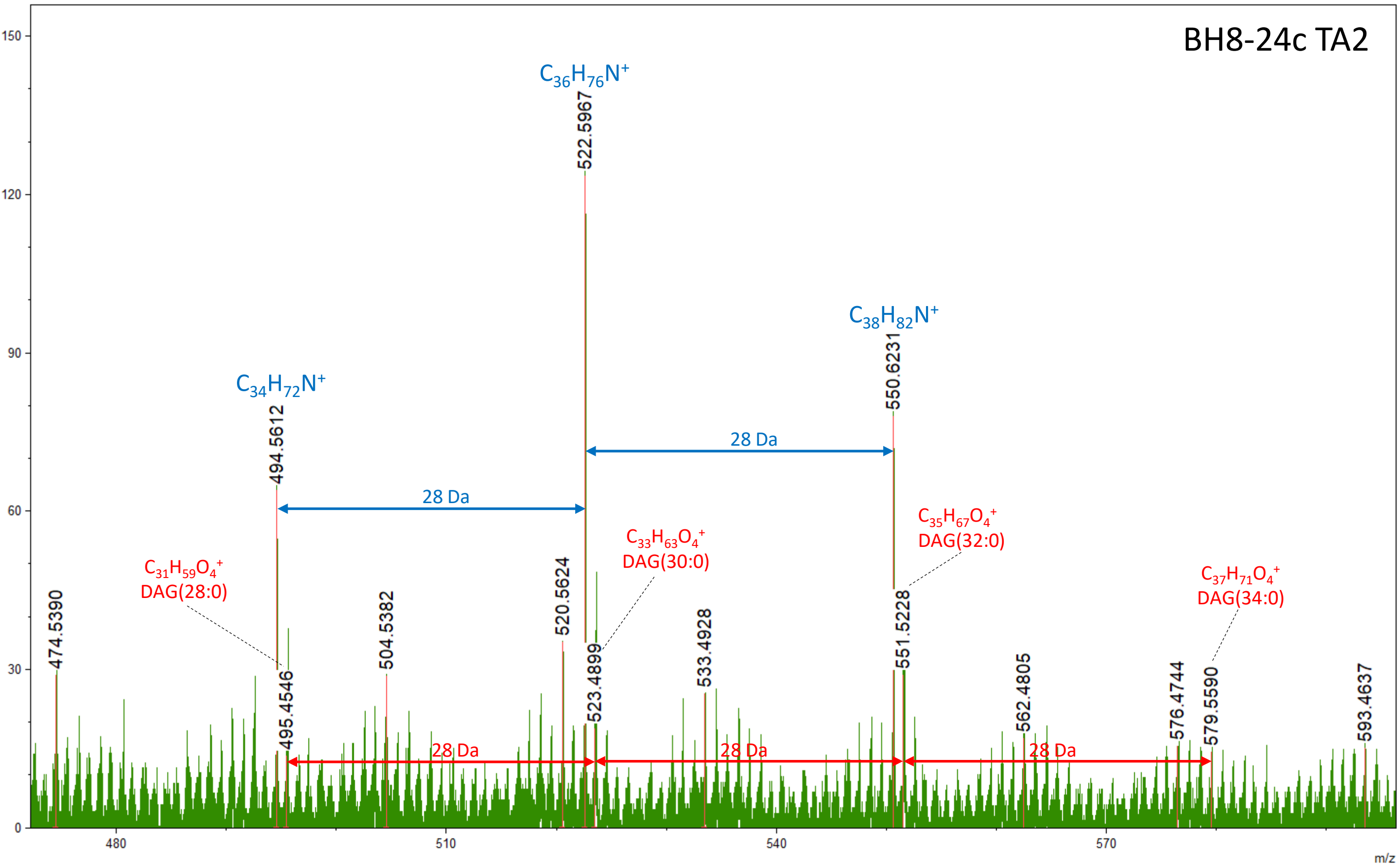

Supplementary Figure 6. Mass spectrum (intensity units in cps) in the range of 450 to 600 Da showing different peaks of NH_4^+-adducts and dyacylglycerides found in TA2.

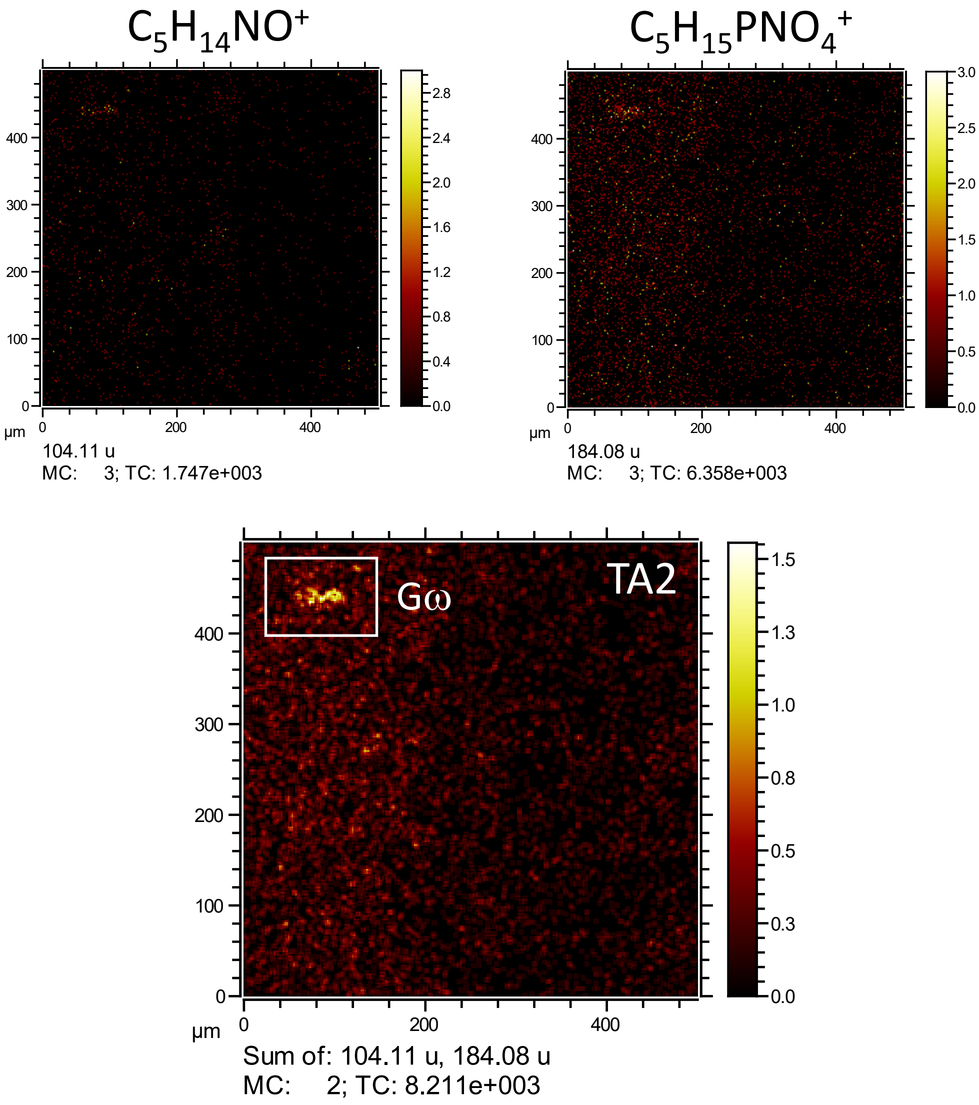

Supplementary Figure 7. ToF-SIMS images of cations tentatively identified as fragments of a phosphocholine derivative occurring at a higher concentration in the Gω microstructure found in TA2.

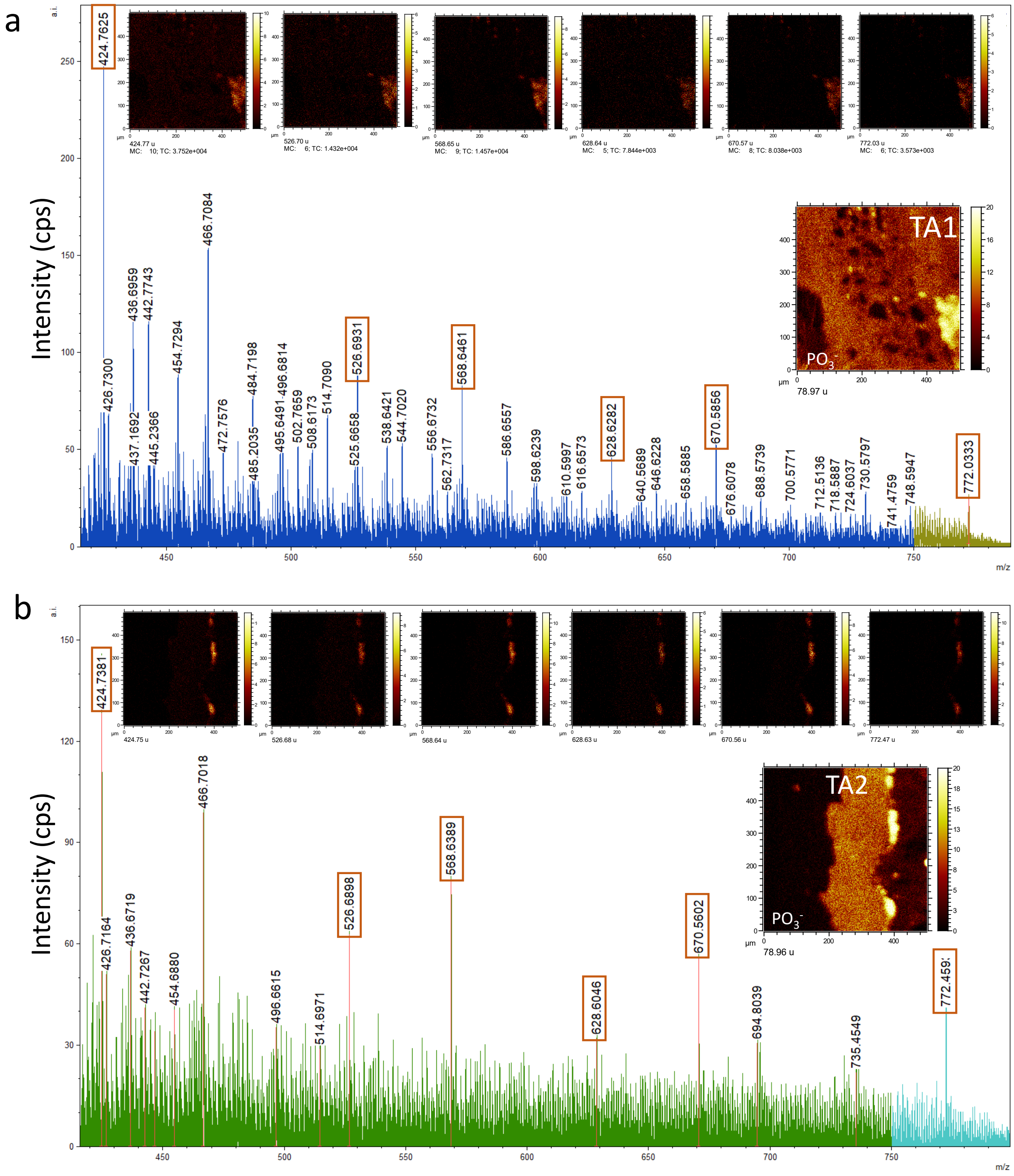

Supplementary Figure 8. Mass spectra (intensity units in cps) showing the distribution of P-bearing compounds in TA1 (a) and TA2 (b) corresponding with phosphatic salt fragments and phospholipid adducts (Supplementary Table 6).

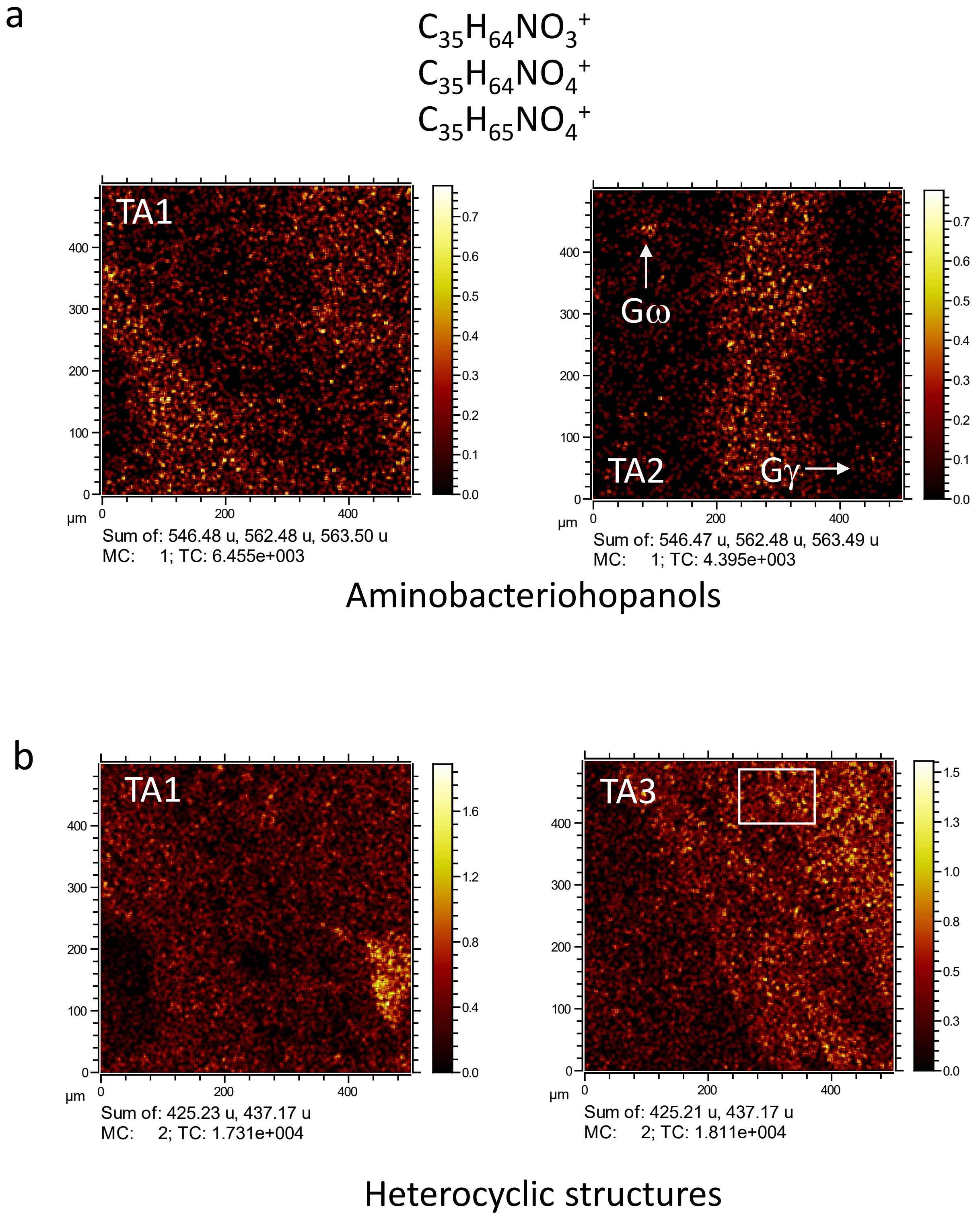

Supplementary Figure 9. ToF-SIMS images of adducts that have been tentatively identified as aminobacteriohopanol (a) and heterocyclic compounds (b).

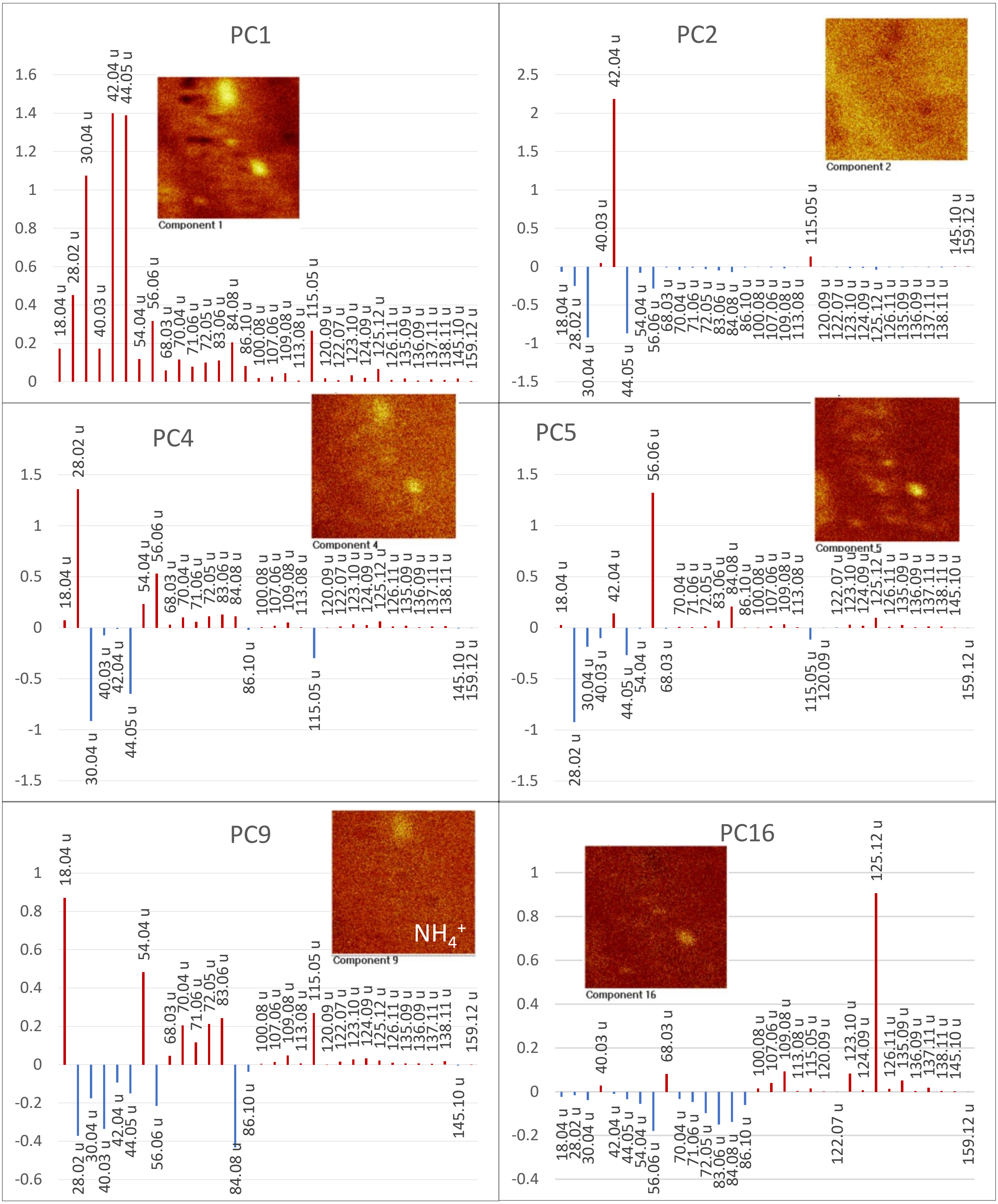

Supplementary Figure 10. Principal component analysis (PCA) performed for the ToF-SIMS cation data (see Supplementary Table 7) that have been identified as amino acid fragments in TA1. For this analysis, 32 cations have been considered. The results suggests that in TA1 the main source of amino acid fragments comes from the different sulfate-rich clasts embedded in the microbrecciated layer (mb). In turn, PC2 and PC5 show that some fragments (e.g., *m/z* 42.04, and 56.06) are secondarily originated in the microlaminated and silica-rich fibrous-cryptocrystalline layers.

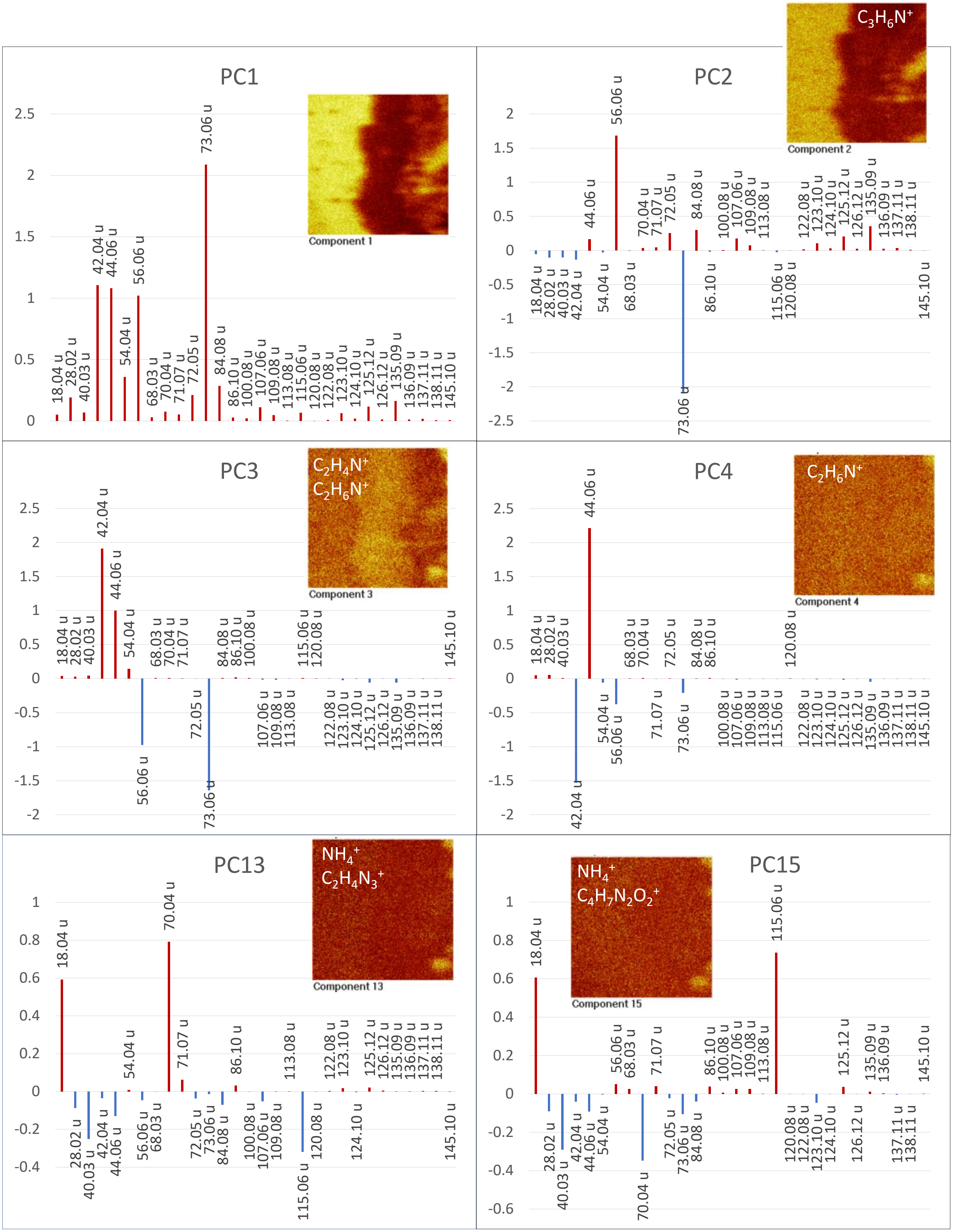

Supplementary Figure 11. Principal component analysis (PCA) performed for the ToF-SIMS cation data (see Supplementary Table 7) that have been identified as amino acid fragments in TA2. To perform this analysis, 30 N-bearing cations have been considered. The PCA has revealed three different areas sourcing the amino acid fragments as the glassy layer (PC1 and PC2), the microlaminated layer (PC3), and the NH_4^+-bearing micronodules (PC4, PC13, and PC15).

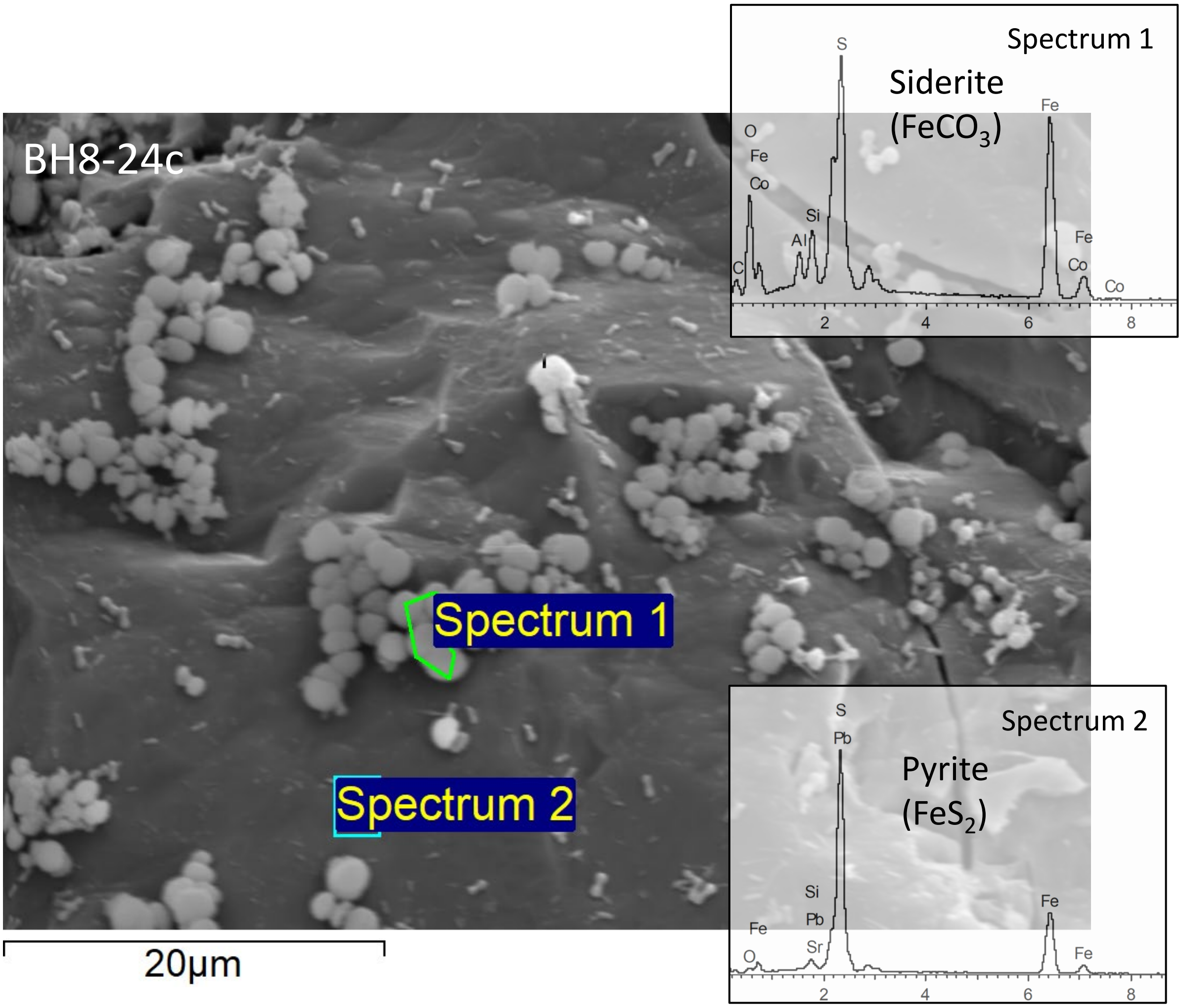

Supplementary Figure 12. SEM-EDAX image showing the occurrence of different carbonatic microstructures resulting from the microbial biomineralization in the sample BH8-24c collected in the Peña de Hierro basement.

Microstructure Molecular Association (MMA)	Microstructure	Morphological groups	Inorganic cations	Inorganic anions	Organic cations	Organic Major organic anions	Organic anions	Possible origin
MMA1	100 micron long crenulated microstructures with fine (< 1 -20 microns) laminas. It migh also appear as small (< 20 micron) micronodular units in the microlaminated layer	Gφ	K > Na	PO_2^-,PO_3^-, $NaPO_4H^-$, and larger inorganic fragments of phosphatic salts<br>Secondary SO_3^-, and SO_4^-	n/o	COP^- > CON^- > CN^-	$C_{38}H_{73}NO_6P^-$ (e.g., PE(O-16:0/17:1), and $C_{43}H_{67}NO_9P^-$ (e.g., PS(17:2/20:5)<br>Heterocyclic compounds like $C_{26}H_{33}O_5^-$, and $C_{16}H_{29}N_4O_8S^-$/$C_{24}H_{25}N_2O_6^-$	PO_4 microbial biomineralization (*Tessaracoccus* and *Acidiphillium*)
MMA2	< 20 micron-sized Micronodules inside the glassy layer	Gω	n/o	PO_2^-, and PO_3^-	Acylglyceride fragments and adducts (e.g., $C_{33}H_{63}O_4^+$ (DAG(30:0)), $C_{35}H_{67}O_4^+$ (DAG(32:0)), and $C_{37}H_{71}O_4^+$ (DAG(34:0))<br>Phosphocoline ($C_5H_{14}NO^+$, $C_5H_{15}PNO_4^+$) and aminohopanols (e.g., $C_{35}H_{64}NO_3^+$, $C_{35}H_{64}NO_4^+$, and $C_{35}H_{65}NO_4^+$) fragments	CNO^-, CN^-	FAs dominated by $C_{16:0}$ > $C_{14:0}$ showing internal variation where $C_{14:0}$ > $C_{15:0}$<br>$C_{12}H_{25}SO_4^-$, and $C_{14}H_{29}SO_4^-$<br>$C_3H_7O_3^-$, $C_4H_3O_5^-$	Degradation of endolithic microbes
MMA3	circular nodules (< 10 microns) in the glassy and microlaminated layeres	Gα(1)	K^+, Na^+	NO_2^- and NO_3^-<br>PO_2^-, and PO_3^-	n/o	CNO^-, CN^-	FAs ($C_{16:0}$ > $C_{14:0}$)<br>$CH_4PO_3^-$	Anammox bacteria
MMA4	60 micron sized nodular structures mineralized by ferric oxysulfates	Gο	NH_4^+ > NO_2^-<br>Fe^+, FeO^+, $Fe_2O_2^+$	FeO_2^-,$Fe_2H_5O_2^-$, $FeSO_5H^-$	C_2H_4N+, $C_2H_6N^+$, $C_2H_8N^+$, $C_2H_4N_3^+$, $C_3H_8N^+$, $C_4H_{10}N^+$, $C_4H_7N_2O_2^+$ (and more peptide fragments)	CSN^-	n/o	Protein decomposers (ammonification)
MMA5	Thick (> 200 microns) glassy layer	Gα(2)	Na^+ > K^+	SO^-, SO_2^-, SO_3^-, SO_4^-	$C_2H_4N^+$, $C_2H_6N^+$, $C_3H_6N^+$	CNO^-, CN^-	$C_{14}H_{13}NO^-$, $C_{15}H_{15}NO^-$, $C_{15}H_{17}NO^-$, and $C_{16}H_{17}NO^-$	Mineralization of Extracellular Polymeric Substances of biofilms by sulfate
		Gκ	K^+		$C_{19}H_{42}N^+$, $C_{34}H_{72}N^+$, $C_{36}H_{76}N^+$, and $C_{38}H_{80}N^+$			Sphingolipid bacterial synthesizers

Supplementary Table 1. Association between distinctive microstructures with potential biological origin and characteristic ions that have been used to characterize the association between microstructures and molecular compounds (MMAs).

Sample	Group	Area	Subgroups	m/z^+	m/z^-	Potential compounds	
						m/z^+	m/z^-
**BH8-24c**	Group α	TA1		42.04 ($C_2H_4N^+$), 44.05 ($C_2H_6N^+$), 54.04 ($C_3H_4N^+$), 68.03 ($C_2H_2N_3^+$), 70.04 ($C_2H_4N_3^+$), 71.06 ($C_3H_7N_2^+$), 72.05 ($C_2H_6N_3^+$), 83.06 ($C_4H_7N_2^+$), 107.06 ($C_6H_7N_2^+$/$C_7H_7O^+$), 109.08 ($C_6H_9N_2^+$), 113.08 ($C_6H_{11}NO^+$), 122.08 ($C_6H_8N_3^+$), 123.10 ($C_8H_{13}N^+$), 124.10 ($C_6H_{10}N_3^+$/$C_7H_{12}N_2^+$), 126.11 ($C_7H_{12}N_2^+$), and 138.11 ($C_7H_{12}N_3^+$/$C_8H_{14}N_2^+$)	Only some micronodules show a maximum intensity in SO_2^-/SO_3^- (m/z^- at 62.97/79.97), and S_xO_y-bearing negative ions like $C_5H_9SO_4^-$ (167.02), $C_{12}H_{23}SO_4^-$ (263.13), $C_{12}H_{25}SO_4^-$ (265.15), $C_{12}H_{26}SO_4^-$ (266.15), $C_{12}H_{27}SO_4^-$ (267.15), $C_{13}H_{27}SO_4^-$ (279.17), $C_{13}H_{28}SO_4^-$ (280.17), $C_{14}H_{29}SO_4^-$ (293.18), $C_{14}H_{30}SO_4^-$ (294.18), $C_{14}H_{31}SO_4^-$ (295.16), $C_{14}H_{29}SO_5^-$ (309.17), $C_{16}H_{33}SO_5^-$ (337.20), $C_{20}H_{33}SO_3^-$ (353.22) and $C_{17}H_{35}S_2O_4^-$ (367.22), while is highly depleted in Fe^+	Fragments of amino acids and peptides	Sulfate adducts formed from a organic surface mineralized by sulfate
					26.00 (CN^-), 90.01 ($C_4N_3^-$), 91.03, 92.03, 93.04, 117.44, 119.05, and 133.07 corresponding to $C_5H_3N_2^-$, $C_5H_4N_2^-$, $C_5H_5N_2^-$, $C_4H_9NO_3^-$, $C_8H_9N_2^-$, and $C_7H_7N_2O^-$		Potential fragments of amino acids or proteins occurring with termenoids bearing carboxylic radicals
					211.09, 225.11, 227.13, and 239.11 could correspond with $C_{14}H_{13}NO^-$, $C_{15}H_{15}NO^-$, $C_{15}H_{17}NO^-$, and $C_{16}H_{17}NO^-$		Degradation compounds of proteins
					227.20, and 323.20, are assigned to $C_{14}H_{27}O_2^-$, and $C_{22}H_{27}O_2^-$		Occurrence of myristic acid ($C_{14:0}$) associated to terpene-like cyclic with carboxilic groups
		TA2			NO_2^-, and NO_3^-		Formation of nitrates by Ammanox bacteria
			Gα(1)		79.97 ($PSNH_3^-$), 80.97 ($CSNNa^-$), 81.96 ($CHSNNa^-$), 95.97 ($SNO_3H_3^-$), 107.99 ($C_5H_2SN^-$), and 105.98 ($C_2H_4S_2N^-$); as well as different organic negative ions like 26.01 (CN^-), 41.01 (CHN_2^-), 44.02 (CH_2NO^-), 50.01 ($C_4H_2^-$), 51.03 ($C_4H_3^-$), 58.04 ($CH_4N_3^-$/$C_2H_4NO^-$), 59.01 ($C_2H_3O_2^-$), 60.01 ($C_2H_4O_2^-$/$CH_2NO_2^-$), 61.01 ($CH_3NO_2^-$), 62.02 ($CH_4NO_2^-$), 64.03 ($C_5H_4^-$), 65.01 ($C_3HN_2^-$), 65.05 ($C_5H_5^-$), 66.01 ($C_2N_3^-$/$C_4H_2O^-$), 83.03 ($C_4H_5NO^-$), 83.06 ($C_4H_7N_2^-$), 84.02 ($C_2H_2N_3O^-$/$C_4H_4O_2^-$), 84.06 ($C_5H_8O^-$/$C_3H_6N_3^-$), 86.03 ($C_3H_4NO_2^-$), 88.03 ($C_7H_4^-$), 89.05 ($C_4H_9O_2^-$), 99.03 ($C_3H_3N_2O_2^-$),100.02 ($C_3H_4N_2O_2^-$), 104.04 ($C_3H_6NO_3^-$), 105.04 ($C_3H_7NO_3^-$), 107.07 ($C_3H_9NO_3^-$), 108.04 ($C_5H_4N_2O^-$/$C_2H_6NO_4^-$), 130.99 ($C_4H_3O_5^-$), and 141.10 ($C_8H_{13}O_2^-$)		Potential amino acids and protein fragments co-occurring with terpenoids bearing carboxylic radicals
			Gα(2)		92.03 ($C_2H_6NO_3^-$), 93.05 ($C_2H_7NO_3^-$), 99.02 ($C_3H_3N_2O_2^-$), 111.03 ($C_5H_5NO_2^-$), 117.04 ($C_4H_7NO_3^-$), 119.05 ($C_4H_9NO_3^-$), 133.08 ($C_5H_{11}NO_3^-$), 135.06 ($C_5H_{11}O_4^-$), 143.06 ($C_9H_7N_2^-$/$C_6H_9NO_3^-$), 163.08 ($C_{10}H_{11}O_2^-$), 165.05 ($C_9H_9O_3^-$), 183.02 ($C_7H_{14}PO_4^-$), 211.10 ($C_{14}H_{13}NO^-$), 212.10 ($C_{14}H_{14}NO^-$), 221.09 ($C_{12}H_{13}O_4^-$), 223.04 ($C_{14}H_7O_3^-$), 227.12 ($C_{15}H_{17}NO^-$), 237.10 ($C_{16}H_{13}O_2^-$/$C_{13}H_{17}O_4^-$), 238.10 ($C_{16}H_{14}O_2^-$), 239.10 ($C_{16}H_{17}NO^-$), 272.11 ($C_{16}H_{21}NO_2S^-$/$C_{14}H_{14}N_3O_3^-$), 297.07 ($C_{13}H_{13}O_8^-$), 298.07 ($C_{13}H_{14}O_8^-$), 349.25 ($C_{21}H_{33}O_4^-$), and 377.28 ($C_{23}H_{37}O_4^-$)		
	Group β	TA1		the m/z^+ ions at 43.02, 45.04, 47.01, 57.04, 59.05, 60.02, 61.03, 69.04, 71.01, 73.03, 81.04, 85.03, 87.05, 97.03, 101.03, 150.12, and 202.24, matching $C_2H_3O^+$, $C_2H_5O^+$, $CH_3O_2^+$, $C_3H_5O^+$, $C_3H_7O^+$, $C_2H_4O_2^+$, $C_2H_5O_2^+$, $C_4H_5O^+$, $C_3H_3O_2^+$, $C_3H_5O_2^+$, $C_5H_5O^+$, $C_4H_5O_2^+$, $C_4H_5O_2^+$, $C_4H_7O_2^+$, $C_5H_5O_2^+$, $C_3H_5N_2O_2^+$/$C_8H_5^+$, $C_{10}H_{14}O^+$/$C_{10}H_{16}N^+$, and $C_{12}H_{30}N_2^+$/$C_{13}H_{32}N^+$	negative ion fragments at 45.00, 53.01, 55.02, 58.01, 59.02, 69.00, 69.04, 83.02, 84.02, 85.04, 86.01, 87.01, 99.02, 101.03, 111.02, 113.04, 116.03, and 125.04, which are assigned to CHO_2^-, C_3HO^-, $C_3H_3O_-$, $C_2H_2O_2^-$, $C_2H_3O_2$-, $C_3HO_2^-$, $C_4H_5O^-$, $C_4H_3O_2^-$, $C_4H_4O_2^-$, $C_4H_5O_2^-$, $C_3H_2O_3^-$, $C_3H_3O_3^-$, $C_4H_3O_3^-$, $C_4H_5O_3^-$, $C_5H_3O_3^-$, $C_5H_5O_3^-$, $C_8H_4O^-$/$C_5H_8O_3^-$, and $C_6H_5O_3^-$		Fragments of resilient heterocyclic compounds
				Several organic m/z^+ cations also show a higher intensity in the internal layer of A2 like 29.04 ($C_2H_5^+$), 31.02 (CH_3O^+), 41.00 (C_2HO^+), 43.02 ($C_2H_3O^+$), 45.04 ($C_2H_5O^+$), 53.04 ($C_4H_5^+$), 461.57 (unknown), 474. 53 ($C_{34}H_{66}^+$), and 492.56 ($C_{35}H_{72}^+$/$C_{34}H_{70}N^+$)			
				55.93 (Fe^+), 56.96 ($CaOH^+$), 64.97 (SO_2H^+), 99.94 ($CaCO_3^+$), 144.86 ($Fe_2O_2H^+$), 258.76 ($H_3O_4Ti_4^+$), 402.61 (S-Fe-unknown positive fragment), and 431.55 ($Fe_6O_6^+$)		Mineralization by acidic soutions	
		TA2	Gβ(1)	*m/z* $^+$ peaks a t 26.98, 31.02, 41.00, 56.96, and 112.98 corresponding to Al^+, CH_3O^+, C_2HO^+, $CaOH^+$, and HS_3O^+	*m/z* $^-$ fragments like 63.96, 79.96, 183.02, 265.15, 279.16, 293.18, 301.21, 309.17, 311.16, 312.21, 325.18, 337.20, 353.20, 381.22, and 397.22, which correspond to different S-bearing cations assigned to SO_2^-, SO_3^-, $C_8H_7SO_3^-$, $C_{12}H_{25}SO_4^-$, $C_{13}H_{27}SO_4^-$, $C_{14}H_{29}SO_4^-$, $C_{14}H_{29}SO_5^-$, $C_{17}H_{27}SO_3^-$, $C_{18}H_{32}SO_2^-$, $C_{18}H_{29}SO_3^-$, $C_{16}H_{33}SO_5^-$/$C_{19}H_{29}SO_3^-$, $C_{20}H_{33}SO_3^-$, $C_{18}H_{37}SO_6^-$, and $C_{18}H_{37}SO_7^-$.	Major positive cations of the mineral matrix	Sulfate adducts formed from a organic surface mineralized by sulfate
			Gβ(2)	set of inorganic positive cations like 55.93 (Fe^+), 56.96 ($CaOH^+$), 64.97 (SO_2H^+), 99.94 ($CaCO_3^+$), 144.86 ($Fe_2O_2H^+$), 258.76 ($H_3O_4Ti_4^+$), 402.61 (S-Fe-unknown positive fragment), and 431.55 ($Fe_6O_6^+$/$Fe_6SO_4^+$)	*m/z* $^-$ 88.93, 168.88, and 250.84, which fit well Fe- and S-bearing negative ions like FeO_2H^-, $FeSO_5H^-$, and $CH_7Fe_3O_4^-$, respectively. Some m/z^- organic fragments like 24.00, 41.00, 50.02, 51.02, 189.00, 203.01, 387.21, 411.24, 421.22, 465.61, 473.28, 479.33, 481.32, and 735.44.	Mineralization by acidic soutions	
				m/z $^+$ organic cations 29.04 ($C_2H_5^+$), 31.02 (CH_3O^+), 41.00 (C_2HO^+), 43.02 ($C_2H_3O^+$), 45.04 ($C_2H_5O^+$), 53.04 ($C_4H_5^+$), and 125.00 ($C_2H_6PO_4^+$). In addition, some unknown peaks at 461.58, 474.55, and 492.59	*m/z* $^-$ 24.00, 41.00, 50.02, 51.02, 189.00, 203.01, 387.21, 411.24, 421.22, 473.28, 479.33, 481.32, and 735.44 that correspond with C_2^-, C_2HO^-, CH_6S^-/$C_4H_2^-$, $C_4H_3^-$, $C_6H_5O_7^-$, $C_7H_7O_7^-$, $C_{23}H_{31}O_5^-$/$C_{26}H_{27}O_3^-$, $C_{23}H_{39}O_6^-$/ $C_{26}H_{35}O_4^-$, $C_{23}H_{33}O_7^-$, $C_{24}H_{41}O_9^-$/$C_{31}H_{37}O_4^-$, $C_{27}H_{45}O_7^-$/$C_{31}H_{45}O_4^-$, $C_{31}H_{45}O_5^-$ /$C_{27}H_{45}O_8^-$, and $C_{51}H_{59}O_4^{-}$/$C_{44}H_{63}O_9^-$/$C_{47}H_{59}O_7^-$.	Lipid fragments	Phenalenones/Silesterol-atrovenetin compounds, and larger heterocyclic structures
	Group γ	TA2		18.04, 58.07, 78.08, 161.10, 214.24, and 242.28 define several 80-micron long egg-shaped micronodules that are found at the edge of the internal layer (Fig. A2). Such positive ions match well NH_4^+, $C_3H_8N^+$, $C_4H_{10}N^+$, $C_6H_{15}N_3O_2^+$, $C_{14}H_{32}N^+$, and $C_{16}H_{36}N^+$		Potential amino acid fragments. $C_{14}H_{32}N^+$ and $C_{16}H_{36}N^+$ may correspond to NH_4^+-bearing adducts of hydrocarbon fragments like $C_{14}H_{28}$ and $C_{16}H_{32}$.	
	Group ε	TA1		unique positive fragment m/z^+ 70.07 ($C_5H_{10}^+$/$C_4H_8N^+$)		Unknown origin	
	Group δ	TA2		m/z^+ fragment 88.08 ($C_4H_{10}NO^+$)		Unknown origin	
	Group φ	TA1		high intensity in the positive ions at 22.99 (Na^+), 38.97 (K^+), 40.97 (H_2K^+), and 94.93 (K_2OH^+/P_2HS^+)	*m/z* $^-$ 62.97 (PO_2^-), 78.97 (PO_3^-), 118.95 ($NaPO_4H^-$), 138.94 ($CHO_4P_2^-$), 139.94 ($CH_2O_4P_2^-$), 155.93 ($CH_3O_3P_3^-$), 365.25 ($C_{18}H_{38}PO_5^-$/$C_{17}H_{34}PO_6^-$), 425.23 ($C_{19}H_{38}PO_8$/$C_{26}H_{33}O_5^-$), 437.17 ($C_{16}H_{29}N_4O_8S^-$/$C_{24}H_{25}N_2O_6^-$), 568.65 ($C_{35}H_{76}N_4O^-$/$C_{36}H_{76}N_2O_2^-$), 628.64 ($C_{42}H_{78}NO_2^-$), 670.57 ($C_{41}H_{77}NO_3K^-$/$C_{40}H_{80}NO_6^-$), and 730.56 ($C_{41}H_{81}NO_7P^-$)	Highly mineralized matrix in phosphate	phosphatic salt fragments, N- and P- bearing lipids including glycerophospholipids, and heterocyclic structures like polyketides
		TA2			*m/z* $^-$ 58.97,62.96, 78.96, 79.96, 93.98, 95.95, 118.94, 138.93, 178.91, and 180.92 corresponding with CPO^-, PO_2^-, SO_2^-, PO_3^-, SO_3^-, $CH_3PO_3^-$, SO_4^-, $HNaPO_4^-$, $HAlPO_5^-$, $HAlCaPO_5^-$, and $NaP_2O_6^-$. Such a pattern is also followed by larger negative fragments as 424.75, 568.65, 628.63, 670.56, and 772.47 that could correspond with $K_2P_5O_{12}^-$, $C_{37}H_{80}N_2O^-$, $C_{42}H_{78}NO_2^-$, $C_{38}H_{72}NO_8^-$ /$C_{38}H_{73}NO_6P^-$, and $C_{43}H_{67}NO_9P$		
	Group κ	TA1		large positive fragments at 494.57, 522.60, and 550.62 matching well NH_4^+-bearing adducts like $C_{34}H_{72}N^+$, $C_{36}H_{76}N^+$, and $C_{38}H_{80}N^+$		NH_4^+-n-alkene adducts sourced in sphingolipids and other large lipids	
	Group ω	TA2		This group is traced by positive fragments in the 79.02, 495.45, 523.49, 551.52, and 579.56, which fit well to a series of diacylglycerids including $C_5H_3O^+$, $C_{31}H_{59}O_4^+$ (DAG28:0), $C_{33}H_{63}O_4^+$ (DAG30:0), $C_{35}H_{67}O_4^+$(DAG32:0), and $C_{37}H_{71}O_4^+$ (DAG34:0)	The *m/z* $^-$ fragments occuring at 127.09, 155.12, 157.14, 169.13, 197.17, 199.17, 227.21, 241.22, 253.23, 255.24, 269.25, 281.26, and 283.28, corresponding to fatty acid fragments like $C_7H_{11}O_2^-$ ($C_{7:1}$), $C_9H_{15}O_2^-$($C_{9:1}$), $C_9H_{17}O_2^-$ ($C_{9:0}$), $C_{10}H_{17}O_2^-$ ($C_{10:1}$), $C_{12}H_{21}O_2^-$ ($C_{12:1}$), $C_{12}H_{23}O_2^-$ ($C_{12:0}$), $C_{14}H_{27}O_2^-$ ($C_{14:1}$), $C_{15}H_{29}O_2^-$ ($C_{15:0}$), $C_{16}H_{29}O_2^-$ ($C_{16:1}$), $C_{16}H_{31}O_2^-$ ($C_{16:0}$), $C_{17}H_{33}O_2^-$ ($C_{17:0}$), $C_{18}H_{33}O_2^-$ ($C_{18:1}$), and $C_{18}H_{35}O_2^-$ ($C_{18:0}$)	Acyldyacylglicerides	
	Group ο	TA1			Different negative ions occurring in TA1 and TA2 like 19.00 (F^-), 32.98 (HS^-), and 34.97 (Cl^-)		Various sources
		TA2					

Supplementary Table 2. Identification of the different morphological groups through the molecular distribution in the underground ferruginous materials of Peña de Hierro using the ion mapping through the ToF-SIMS.

Compound	Calculated m/z (Da)	BH8-24c-TA1		BH8-24c-TA2	
		Measured m/z (Da)	error (ppm)	Measured m/z (Da)	error (ppm)
$C_3H_5^+$	41.04	41.04	-77.97	41.03	365.50
$C_3H_7^+$	43.05	43.06	-155.61	43.06	-192.80
$C_4H_7^+$	55.05	55.06	-134.41	55.06	-128.97
$C_4H_9^+$	57.07	57.07	-22.78	57.07	-80.60
$C_5H_9^+$	69.07	69.07	-39.09	69.07	-36.20
$C_6H_{11}^+$	83.09	83.09	16.85	83.09	34.90
$C_6H_{13}^+$	85.10	85.10	-12.93	85.10	-27.03
$C_7H_{13}^+$	97.10	97.10	-25.75	97.10	15.45
$C_7H_{15}^+$	99.12	99.12	34.30	99.12	33.29
$C_{13}H_{30}N^+$	200.24	200.24	2.50	200.23	30.96
$C_{14}H_{32}N^+$	214.25	214.26	-36.40	214.24	28.00
$C_{16}H_{36}N^+$	242.28	242.29	-33.43	242.28	6.60
$C_{18}H_{40}N^+$	270.32	270.32	6.66	270.31	23.68
$C_{20}H_{44}N^+$	298.35	298.35	6.70	298.34	25.47
$C_{21}H_{46}N^+$	312.36	312.37	-23.69	312.36	-4.16
$C_{22}H_{48}N^+$	326.38	326.38	7.35	326.38	11.95
$C_{25}H_{54}N^+$	368.43	368.41	42.62	368.42	21.44
$C_{34}H_{72}N^+$	494.57	494.59	-30.94	494.56	16.18
$C_{36}H_{76}N^+$	522.60	522.60	0.96	522.60	6.31
$C_{37}H_{78}N^+$	536.61	536.60	19.94	536.59	40.07
$C_{38}H_{80}N^+$	550.63	550.63	7.81	550.62	12.17

Supplementary Table 3. Cation list of hydrocarbon fragments and NH4+ adducts found in TA1 and TA2 of the sample BH8-24c collected in the Peña de Hierro weathered basement.

Compound	Calculated m/z (Da)	BH8-24c-TA1		BH8-24c-TA2	
		Measured m/z (Da)	error (ppm)	Measured m/z (Da)	error (ppm)
$C_9H_7^+$	115.05	115.05	-40.85	115.06	-53.89
$C_{10}H_8^+$	128.06	128.06	-8.59	128.06	-19.52
$C_{11}H_9^+$	141.07	141.07	20.56	141.07	8.51
$C_{12}H_8^+$	152.06	152.06	20.39	152.06	8.55
$C_{13}H_9^+$	165.07	165.06	34.53	165.07	21.81
$C_{14}H_{10}^+$	178.08	178.07	56.15	178.07	49.98
$C_{15}H_9^+$	189.07	189.06	35.97	189.07	22.74
$C_{16}H_{10}^+$	202.08	202.07	56.91	202.07	38.60
$C_{17}H_{11}^+$	215.09	215.08	62.30	215.08	32.54
$C_{18}H_{10}^+$	226.08	-	-	226.07	31.85
$C_{19}H_{11}^+$	239.09	-	-	239.09	-9.20

Supplementary Table 4. List of [M-H]+ and M+ of different polycyclic aromatic hydrocarbons (PAHs) found in the ferruginous underground materials of Peña de Hierro.

Generic fatty acid	Calculated *m/z*	BH8-24c TA1			BH8-24c TA2			BH8-24c TA3		
		Observed *m/z*	error	intensity (cps)	Observed *m/z*	error	intensity (cps)	Observed *m/z*	error	intensity (cps)
Heptenoic $C_{7:1}$ ($C_7H_{12}O_2$)	127.08	127.08	-18.10	130.00	127.08	-11.80	480.00	127.08	-18.10	130.00
Heptenoic $C_{7:0}$ ($C_7H_{14}O_2$)	129.10	129.08	-132.46	70.00	129.10	8.52	250.00	129.08	-132.46	70.00
Octenoic $C_{8:1}$ ($C_8H_{14}O_2$)	141.09	141.09	24.10	84.00	141.10	70.88	355.00	141.09	24.10	98.00
Octenoic $C_{8:0}$ ($C_8H_{16}O_2$)	143.11	143.11	-22.36	175.00	143.10	-40.53	795.00	143.11	-34.94	164.00
Nonenoic $C_{9:1}$ ($C_9H_{16}O_2$)	155.11	155.11	7.74	56.00	155.12	64.47	235.00	155.11	1.93	65.00
Nonenoic $C_{9:0}$ ($C_9H_{18}O_2$)	157.12	157.13	32.46	110.00	157.13	91.01	390.00	157.12	-30.55	95.00
Decenoic $C_{10:1}$ ($C_{10}H_{18}O_2$)	169.12	169.13	48.49	51.00	169.13	84.56	210.00	169.13	46.71	40.00
Decanoic $C_{10:1}$ ($C_{10}H_{20}O_2$)	171.14	171.14	-0.58	135.00	171.14	-5.26	345.00	171.14	-24.54	120.00
Undecanoic acid $C_{11:0}$ ($C_{11}H_{22}O_2$)	185.15	185.16	50.77	43.00	185.17	83.72	120.00	-	-	45.00
Dodecanoic acid $C_{12:0}$ ($C_{12}H_{24}O_2$)	199.17	199.17	-22.98	80.00	199.16	-37.03	355.00	199.17	-16.07	90.00
Tridecanoic acid $C_{13:0}$ ($C_{13}H_{26}O_2$)	213.19	213.18	-35.25	56.00	213.18	-37.61	178.00	213.17	-71.77	50.00
Myristoleic acid $C_{14:1}$ ($C_{14}H_{26}O_2$)	225.19	225.18	-55.06	55.00	225.19	18.65	175.00	-	-	-
Myristic acid $C_{14:0}$ ($C_{14}H_{28}O_2$)	227.20	227.20	-7.92	130.00	227.19	-31.67	1000.00	227.20	-22.01	170.00
Pentadecenoic acid $C_{15:1}$ ($C_{15}H_{28}O_2$)	239.20	239.20	-5.41	43.00	239.21	41.81	200.00	239.20	1.67	65.00
Pentadecanoic/methyl myristic acid $C_{15:0}$ ($C_{15}H_{30}O_2$)	241.22	241.21	-21.14	145.00	241.23	55.55	542.00	241.22	-19.07	140.00
Palmitoleic acid $C_{16:1}$ ($C_{16}H_{30}O_2$)	253.22	253.21	-24.09	130.00	253.23	52.92	250.00	253.22	-8.69	130.00
Palmitic acid $C_{16:0}$ ($C_{16}H_{32}O_2$)	255.23	255.23	10.58	275.00	255.25	94.82	1600.00	255.23	18.81	260.00
Methyl palmytic/Heptadecenoic acid $C_{17:1}$ ($C_{17}H_{32}O_2$)	267.23	267.25	72.97	20.00	267.21	-66.61	120.00	267.23	7.11	47.00
Heptadecanoic acid $C_{17:0}$ ($C_{17}H_{34}O_2$)	269.25	269.25	-17.83	43.00	269.27	69.82	285.00	269.24	-30.08	62.00
Oleic acid $C_{18:1}$ ($C_{18}H_{34}O_2$)	281.25	281.25	2.13	70.00	281.24	-48.36	195.00	281.25	-12.09	92.00
Stearic acid $C_{18:0}$ ($C_{18}H_{36}O_2$)	283.26	283.25	-44.84	32.00	283.27	38.83	415.00	283.27	26.12	60.00
Nonadecyl acid/methyl stearate; nonadecanoic acid $C_{19:0}$ ($C_{19}H_{38}O_2$)	297.28	-	-	-	297.27	-22.20	70.00	-	-	-
Lignoceric/tetracosanoic acid $C_{24:0}$ ($C_{24}H_{48}O_2$)	367.37	-	-	-	367.37	-3.54	55.00	-	-	-
Pentacosanoic acid $C_{25:0}$ ($C_{25}H_{50}O_2$)	381.37	-	-	-	381.39	59.78	50.00	-	-	-
Cerotic acid/ Lauryl myristate $C_{26:0}$ ($C_{26}H_{52}O_2$)	395.39	-	-	-	395.40	12.65	34.00	-	-	-
Heptacosanoic acid $C_{27:0}$ ($C_{27}H_{54}O_2$)	409.40	-	-	-	409.42	43.97	22.00	-	-	-

Supplementary Table 5. List of the FA [M – H]- adducts identified in the three target areas TA1, TA2, and TA3 of the BH8-24c Peña de Hierro sample by using ToF-SIMS.

Compound	Calculated *m/z*	BH8-24c TA1		BH8-24c TA2	
		Observed *m/z*	error	Observed *m/z*	error
		Acylglycerols			
$C_{31}H_{59}O_4^+$	495.44	-	-	495.45	-29.47
$C_{33}H_{63}O_4^+$	523.47	-	-	523.49	-38.02
$C_{35}H_{67}O_4^+$	551.50	551.53	-54.40	551.52	-41.34
$C_{37}H_{71}O_4^+$	579.54	-	-	579.56	-32.78
		Phosphocholine derivative			
$C_5H_{14}NO^+$	104.11	104.11	6.72	104.11	-20.17
$C_5H_{15}PNO_4^+$	184.07	184.08	-36.40	184.07	-15.21
		Sphingolipids			
$C_{18}H_{36}NO^+$	282.28	282.29	-32.24	282.29	-38.26
$C_{18}H_{38}NO^+$	284.30	284.30	3.17	284.32	-83.71
$C_{20}H_{38}NO^+$	308.30	308.29	29.19	308.31	-38.60
$C_{20}H_{40}NO^+$	310.31	310.32	-33.84	310.33	-61.87
$C_{37}H_{74}NO_2^+$	564.57	564.55	36.31	564.58	-25.33
$C_{37}H_{72}NO_4^+$	594.55	594.54	14.97	594.50	76.36
$C_{41}H_{82}NO_5^+$	668.62	668.62	6.58	668.61	21.54
		Phospholipids			
$C_{38}H_{73}NO_6P^-$	670.52	670.59	-97.83	670.56	-59.95
$C_{41}H_{82}NO_7P^-$	730.58	730.58	5.61	-	-
$C_{41}H_{71}O_{10}P^-$	735.46	-	-	735.45	6.93
$C_{43}H_{67}NO_9P^-$	772.46	-	-	772.45	12.95

Supplementary Table 6. Different positive and negative ions of lipids obtained through the ToF-SIMS spectral analysis in TA1 and TA2 of sample BH8-24c.

Compound	Calculated *m/z*	BH8-24c TA1		BH8-24c TA2	
		Observed *m/z*	error	Observed *m/z*	error
Major ions					
HS^-	32.98	32.98	-30.32	32.98	51.55
CON^-	42.00	42.00	-111.90	42.01	-154.76
CNS^-	57.98	57.98	12.07	57.98	-56.92
Amino acid/peptide fragments (Gα)					
CH_2N^+	28.02	28.03	-288.72	28.03	-306.92
CH_4N^+	30.03	30.03	-136.53	30.04	-206.46
$C_2H_4N^+$	42.03	42.04	-249.82	42.04	-256.96
$C_2H_6N^+$	44.05	44.05	-111.24	44.05	-68.10
$C_3H_4N^+$	54.03	54.04	-223.95	54.04	-216.55
$C_3H_6N^+$	56.05	56.06	-96.34	56.06	-137.38
$C_2H_2N_3^+$	68.02	68.03	-94.09	68.03	-107.32
$C_3H_7N_2^+$	71.06	71.06	42.22	71.07	-91.47
$C_2H_6N_3^+$	72.06	72.05	149.88	72.05	123.51
$C_3H_7NO^+$	73.05	73.05	2.74	-	-
$C_2H_7N_3^+/C_4H_9O^+$	73.07	73.06	17.79	73.06	102.65
$C_4H_7N_2^+$	83.06	83.06	7.22	83.06	-55.38
$C_5H_{10}N^+$	84.08	84.08	36.87	84.08	44.01
$C_5H_{12}N^+$	86.10	86.10	16.26	86.10	31.36
$C_4H_{10}N_3^+$	100.09	100.08	72.93	100.08	80.93
$C_6H_7N_2^+$	107.06	107.06	26.15	107.06	21.48
$C_6H_9N_2^+$	109.08	109.08	11.92	109.08	-26.59
$C_6H_{11}NO^+$	113.08	113.08	21.22	113.08	-3.54
$C_4H_7N_2O_2^+$	115.05	115.05	-40.85	115.06	-48.85
$C_8H_{10}N^+$	120.08	120.09	-72.37	120.09	-44.97
$C_6H_8N_3^+$	122.07	122.07	-36.86	122.08	-87.65
$C_8H_{13}N^+$	123.10	123.10	13.81	123.10	1.62
$C_7H_{12}N_2^+$	124.10	124.09	66.08	124.10	-11.28
$C_7H_{13}N_2^+$	125.11	125.11	-35.97	125.12	-58.35
$C_8H_{11}N_2^+$	135.09	135.09	2.96	135.09	-14.06
$C_8H_{12}N_2^+$	136.10	136.09	88.91	136.09	45.55
$C_8H_{13}N_2^+$	137.11	137.11	-9.48	137.12	-42.30
$C_7H_{12}N_3^+$	138.10	138.11	-41.27	138.11	-98.48
$C_6H_{13}N_2O_2^+$	145.10	145.10	-12.41	145.10	-22.74
$C_8H_9N_3^+/C_5H_{11}N_2O_3^+$	147.08	147.08	15.64	147.08	31.96
$C_7H_{15}N_2O_2^+$	159.11	159.12	-50.91	159.11	-30.17
Amino acid/peptide fragments (Gγ)					
NH_4^+	18.03	18.04	-343.87	18.04	-310.59
$C_3H_8N^+$	58.07	58.07	29.28	58.07	37.89
$C_4H_{10}N^+$	72.08	72.08	-45.78	72.08	-27.75
$C_9H_{11}N_3^+$	161.10	161.10	1.86	161.10	-9.93
Amino acid/peptide fragments (Gα, and Gβ)					
C_4H^-	49.01	49.01	14.28	49.01	-93.86
$C_3HN_2^-$	65.01	65.01	35.23	65.01	-15.38
$C_4N_3^-$	90.01	90.01	52.22	90.01	-8.89
$C_5H_3N_2^-$	91.03	91.03	43.94	91.03	7.69
$C_5H_4N_2^-$	92.04	92.03	130.38	92.04	22.82
$C_5H_5N_2^-$	93.05	93.04	95.65	93.05	19.34
$C_3H_3N_2O_2^-$	99.02	99.02	49.48	99.02	-30.30
$C_4H_7NO_3^-$	117.04	117.04	22.21	117.05	-46.99
$C_4H_9NO_3^-$	119.06	119.05	46.20	119.06	-11.76
$C_8H_9N_2^-$	133.08	133.07	70.63	133.08	15.03
$C_7H_7N_2O^-$	135.06	135.06	31.10	135.06	11.11
$C_9H_7N_2^-$	143.06	143.05	46.13	143.06	-18.17
$C_5H_{11}N_2O_3S^-$	179.05	179.04	44.68	179.05	-7.82
$C_{13}H_{27}N_2O_3S^-$	291.17	291.16	25.76	291.17	-15.80

Supplementary Table 7. List of positive and negative ions produced from the fragmentation of preserved peptidic and/or amino acids obtained in TA1 and TA2 by the ToF-SIMS spectral analysis of sample BH8-24c obtained in the underground ferruginous materials of Peña de Hierro.

Compound	Calculated *m/z*	BH8-24c TA1		BH8-24c TA2	
		Observed *m/z*	error	Observed *m/z*	error
		Bacteriohopanepolyols			
$C_{13}H_{21}^{+}$	177.16	177.17	-39.51	-	-
$C_{15}H_{23}O^{+}$	219.17	219.18	-43.35	219.18	-50.65
$C_{27}H_{50}N^{+}$	388.39	388.40	-22.14	388.39	-3.35
$C_{36}H_{65}O_{4}^{+}$	561.49	561.49	-8.01	561.49	-1.07
		Aminobacteriohopanol			
$C_{35}H_{64}NO_{3}^{+}$	546.49	546.46	62.40	546.48	26.72
$C_{35}H_{64}NO_{4}^{+}$	562.48	562.49	-10.84	562.48	-0.36

Supplementary Table 8. List of fragments attributed to different sterols and hopanoids that have been recognized in TA1 and TA2 of sample BH8-2c through the ToF-SIMS spectral analysis.
